# Activity and Biocatalytic Potential of an Indolylamide Generating Thioesterase

**DOI:** 10.1021/acs.orglett.4c03648

**Published:** 2024-10-21

**Authors:** Weimao Zhong, Zachary L. Budimir, Lucas O. Johnson, Elizabeth I. Parkinson, Vinayak Agarwal

**Affiliations:** † School of Chemistry and Biochemistry, Georgia Institute of Technology, Atlanta, Georgia 30332, United States; ‡ James Tarpo Jr. and Margaret Tarpo Department of Chemistry, Purdue University, West Lafayette, Indiana 47907, United States; § Borch Department of Medicinal Chemistry and Molecular Pharmacology, Purdue University, West Lafayette, Indiana 47907, United States; ∥ School of Biological Sciences, Georgia Institute of Technology, Atlanta, Georgia 30332, United States

## Abstract

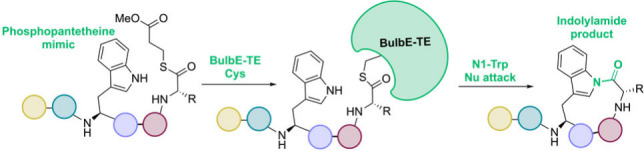

The chemical synthesis of *N*-acyl indoles is hindered by the poor nucleophilicity of indolic nitrogen, necessitating the use of strongly basic reaction conditions that encumber elaboration of highly functionalized scaffolds. Herein, we describe the total chemoenzymatic synthesis of the bulbiferamide natural products by the biochemical activity reconstitution of a nonribosomal peptide synthetase assembly line-derived (NRPS-derived) thioesterase that neatly installs the macrocyclizing indolylamide. The enzyme represents a starting point for biocatalytic access to macrocyclic indolylamide peptides and natural products.

Peptidic natural products furnished by nonribosomal peptide synthetases (NRPSs) are frequently endowed with desirable pharmacological activities. Among these molecules, an often-observed structural feature is macrocyclization. Macrolactams such as cyclosporine, macrolactones such as daptomycin, and peptides macrocyclized by amino acid side chain couplings, such as vancomycin, are examples wherein macrocyclization lends rigidity, proteolytic stability, membrane permeability, and target-engaging conformations to these peptidic natural products.^1^

For NRPS-derived peptides, the macrocyclization catalyst is usually the terminal thioesterase (TE) domain which also offloads the peptide from the NRPS assembly line. The peptide is transesterified from the phosphopantheine thiol of the carrier protein (CP) to generate an acyl intermediate. The TE domain can then use exogenous nucleophiles to release the peptide chain (in red, Figure 1A). Alternatively, the TE can employ intramolecular nucleophiles, such as the N-terminal amine or nucleophilic amino acid side chains to generate macrocyclic products (in blue, Figure 1A).^2,3^

**Figure 1:**
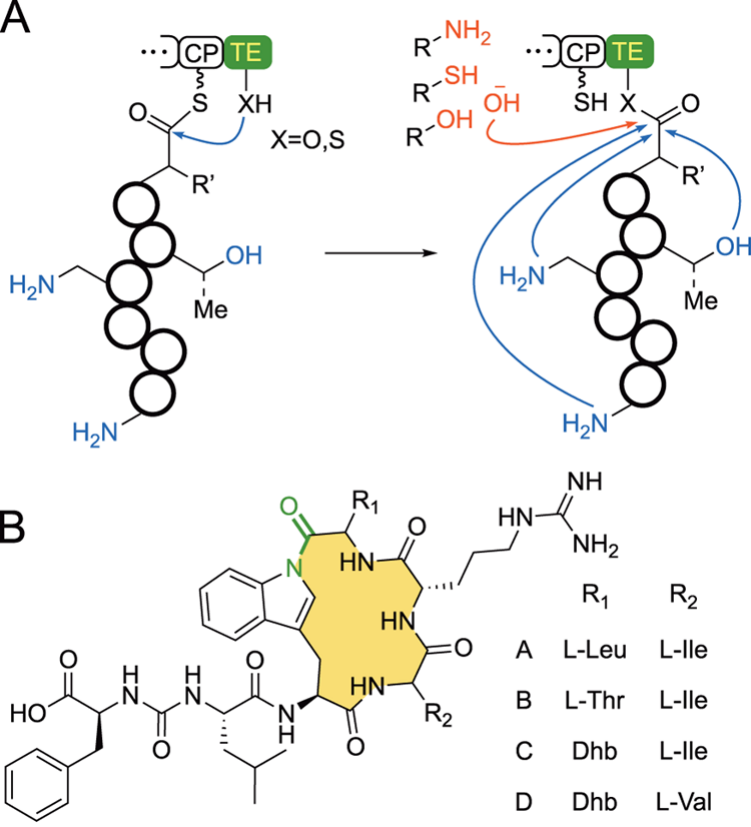
(A) Typical activity of NRPS TE domains wherein they employ inter- or intramolecular nucleophiles to offload the peptide chain. (B) Bulbiferamides A–D; Dhb: dehydrobutyrine. The site of cyclization is highlighted in green.

The discovery of the bulbiferamides, ureidopeptides produced by marine *Microbulbifer* bacteria, led to the observation of a 15-atom macrocycle afforded by amide bond formation with the N-1 position of the tryptophan side chain indole (Figure 1B).^4,5^ Indolylamides are well represented among fungal NRPS-derived alkaloids.^6−9^ In fungi, terminal condensation (C_T_), rather than TE domains, have been implicated in the formation of the acyl indole bond.^10,11^

The production of the bulbiferamides has been attributed to the *bulb* BGCs detected within the *Microbulbifer* spp. genomes. Consistent with bacterial NRPS assembly line architecture, a TE domain at the C-terminus of the BulbE NRPS, henceforth referred to as the BulbE-TE, is positioned at the terminus of the Bulb NRPS assembly line (Figure S1). Thus, the BulbE-TE could conceivably release the peptide from the NRPS assembly line via indolylamide cyclization.^4^

The use of a tryptophan indole side chain nitrogen as a nucleophile for peptide macrocyclization by TEs is unprecedented. Therefore, it was unclear if the BulbE-TE domain was indeed responsible for the formation of the indolylamide macrolactam in bulbiferamides, necessitating experimental validation. This validation could additionally provide a new biocatalyst for a synthetically challenging class of macrocyclizations.

To verify the proposed route for bulbiferamide macrocycle formation, the nucleotide sequence encoding the BulbE-TE domain from *Microbulbifer* sp. MLAF003 was expressed in *Escherichia coli* and the recombinant enzyme purified (Figure S2). Next, we synthesized the peptidic substrates **1** and **2** (Scheme 1), as dictated by the bulbiferamide biosynthetic logic (Figure S1). Here, a linear hexapeptide substrate with a ureido linkage between the l-Phe^1^ and l-Leu^2^ residues must be thioesterified to an upstream CP phosphopantetheine appendage.

**Scheme 1:**
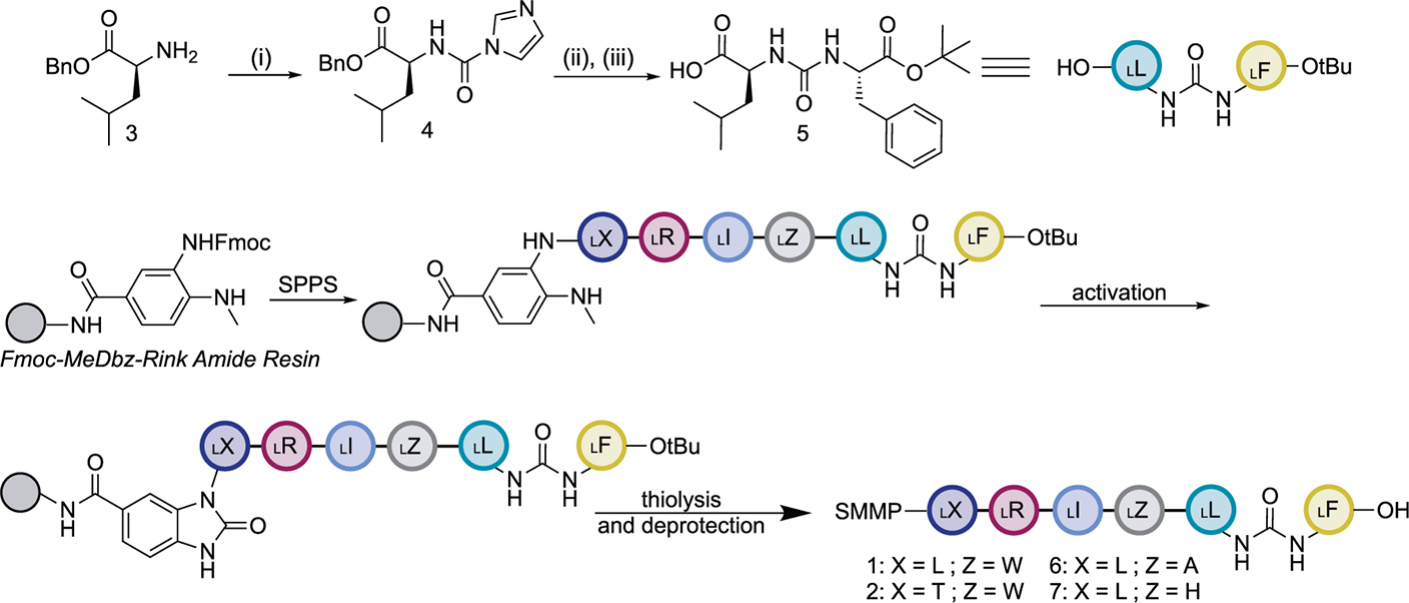
Synthesis of BulbE-TE Substrate Mimics (i) 1.1 equiv. carbonyldiimidazole (CDI), 0.04 equiv. 4-dimethylaminopyridine (DMAP), 3 equiv. triethylamine (TEA), at RT in CH_2_Cl_2_. (ii) 1.2 equiv. l-phenylalanine *tert*-butyl ester hydrochloride, 2.5 equiv. TEA, at RT in CH_2_Cl_2_. 83% yield over two steps. (iii) H_2_, Pd–C (10 mol %), at RT in MeOH. 63% yield. SPPS: solid phase peptide synthesis; SMMP: methyl 3-mercaptoproprionate. Exact conditions can be found in the Supporting Information.

Previous syntheses of peptides featuring an N-terminal urea dipeptide have primarily focused on a class of closely related aldehyde protease inhibitors: GE 20372 and (S)-α- and (R)-β-MAPI (MAPI: Microbial Alkaline Protease Inhibitors).^12−14^ Using a similar approach, the ureidodipeptide **5** was generated off-resin via activation of l-leucine benzyl ester (**3**) with carbonyldiimidazole (CDI) to afford **4**. The crude **4** was then coupled with l-phenylalanine methyl ester in the presence of triethylamine (Figure S3). Removal of the benzyl ester via hydrogenation afforded **5** in suitable purity for solid-phase peptide synthesis (SPPS) (Figure S4). The SPPS of the ureidohexapeptides was accomplished utilizing a safety catch strategy.^15^ The MeDbz linker, which was created by Dawson and co-workers, enabled on-resin activation of the C-terminal amino acid followed by cleavage with a nucleophilic thiol.^16^ This strategy avoids epimerization due to oxazolone formation. Cleavage of the peptides using methyl 3-mercaptoproprionate (SMMP) furnished **1** and **2**; the SMMP moiety serves as a surrogate for the CP phosphopantetheine (Figures S5–S6).^15^

Incubation of **1** and **2** with purified BulbE-TE resulted in production of the natural products bulbiferamides A and B, respectively. The respective thioester hydrolysis side products were also observed (Figures 2 and S7–S8). The retention times and mass spectrometric fragmentation patterns of the macrocyclized products were identical to the bulbiferamide natural product standards (Figure S9). Macrocyclization of **1** proceeded with kinetic parameters *k*_cat_ 0.16 ± 0.02 min^–1^ and *K*_M_ 100 ± 29 μM (Figure S10). No macrocyclized product formation was observed in the absence of the enzyme, or when the active site catalytic Cys was replaced with Ser or Ala (*vide infra*, Figure S11). Taken together, these data establish a chemoenzymatic route to access bulbiferamide natural products while unveiling a novel macrocyclic indolylamide forming activity for TEs.

**Figure 2:**
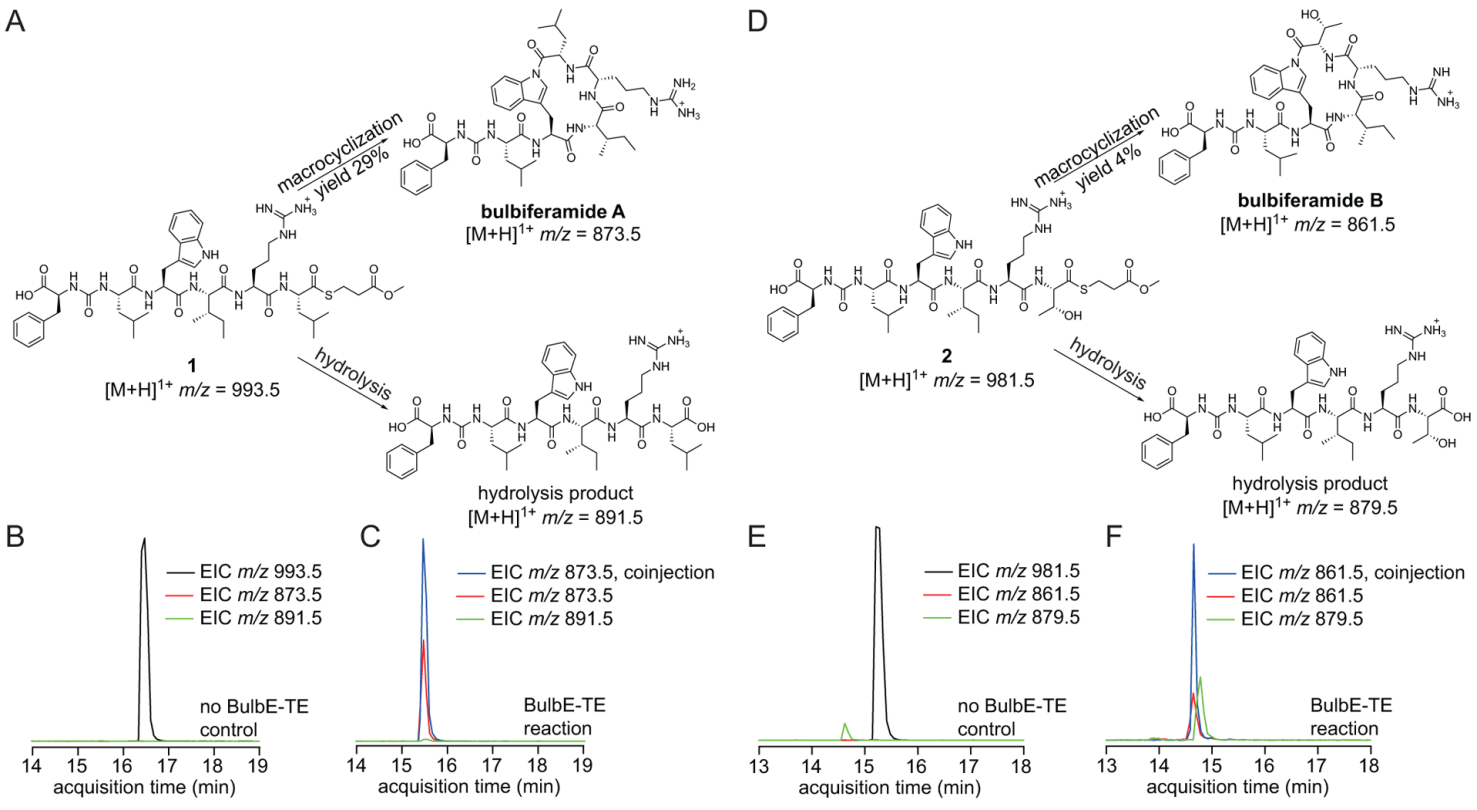
*In vitro* enzymatic activity of BulbE-TE. (A) Macrocyclized and thioester hydrolysis products for substrate **1**. (B) Extracted ion chromatograms (EICs) of **1**, hydrolyzed, and macrocyclized products in the reaction where the BulbE-TE was omitted. (C) EICs of **1**, hydrolyzed, and macrocyclized products in the reaction in the presence of BulbE-TE. The “coinjection” EIC refers to a spiking experiment in which bulbiferamide A was added to the quenched enzymatic reaction to confirm coelution with the macrocyclized enzymatic product. (D) Macrocyclized and thioester hydrolysis products for substrate **2**. (E) EICs demonstrating the presence of **2**, hydrolyzed, and macrocyclized products in the reaction where the BulbE-TE was omitted. (F) EICs of **2**, hydrolyzed, and macrocyclized products in the reaction in the presence of BulbE-TE. As above, the “coinjection” EIC refers to a spiking experiment in which bulbiferamide B was added to the quenched enzymatic reaction to confirm coelution with the macrocyclized enzymatic product.

While the activity of BulbE-TE was thusly validated, the product yields were modest (Table S1). Other marine peptide macrocyclases have likewise been demonstrated to possess reduced catalytic activities.^17,18^ Of note, the yield of bulbiferamide B starting from **2** was lower than that of bulbiferamide A production from **1** (Figure 2). The bacterium *Microbulbifer* sp. MLAF003 does not produce bulbiferamide B—this natural product was isolated from a different strain—*Microbulbifer* sp. VAAF005 which contains a similar *bulb* BGC.^4^ The thioesterase domain from *Microbulbifer* sp. VAAF005 was cloned and expressed. Incubation of **2** with *Microbulbifer* sp. VAAF005-derived BulbE-TE resulted in a near 4-fold increase in bulbiferamide B yield, pointing to the fine-tuning of the TE active site for the different substrates (Figures S12–S14 and Table S1). Replacement of the substrate Trp residue with Ala, corresponding to the thiotemplated ureidopeptide substrate **6**, expectedly did not yield any macrocyclic products (Figures S15–S16). Replacement of Trp with the more nucleophilic His in ureidopeptide substrate **7** also did not yield any macrocyclic products (Figures S17–S18). This is likely due to poor enzymatic recognition of the His containing substrate in the TE active site.

The active site of the BulbE-TE domain was rationalized to possess the Cys961/Asp988/His1097 catalytic triad (amino acid numbering per the *Microbulbifer* sp. MLAF003 BGC). The BulbE-TE-catalyzed transformation is thus expected to proceed via transthioesterification of the substrate peptide to the Cys961-Sγ, followed by resolution of the acyl thioester by the substrate Trp side chain indole via the formation of a tetrahedral thioketal intermediate.^19^ Catalytic Cys residues in TE active sites are suggestive of challenging transformations.^2^ As mentioned above, Ser could not replace Cys in the BulbE-TE active site in line with similar observations for the obafluorin and sulfazecin biosynthetic TE domains—ObiF-TE and SulM-TE—which generate strained 4-atom lactone and lactam products, respectively.^20,21^ Of note, SulM-TE employs an unusual sulfamated amine as the lactam-forming nucleophile. For bulbiferamide biosynthesis, the nucleophilicity of the indole-N is similarly compromised. In light of these observations, the choice of Cys as the preferred active site nucleophile can be rationalized as the acyl-thioester intermediate is much more activated than an acyl-oxoester intermediate for aminolysis.^22,23^ Contrary to aminolysis, oxoesters and thioesters have similar reactivity toward the hydrolysis.^22^ Indeed, while the BulbE-TE Cys961 → Ser mutant does not generate detectable macrocyclized products, it does generate the thioester hydrolysis product in much greater abundance than the wild type enzyme or the Cys961 → Ala mutant (Figure S19).

The AlphaFold3-generated model of the BulbE-TE demonstrates a canonical α/β hydrolase fold with the catalytic Cys and His residues positioned on loops at the end of the β5 and β8 strands of the central β-sheet (Figure S20). Unlike the other Cys-containing NRPS TEs ObiF-TE and the SulM-TE, the catalytic Asp residue in the BulbE-TE is positioned on the loop at the end of the β6 strand, and not the β7 strand.^24,25^

Next, we explored the biocatalytic potential of the BulbE-TE. The physiological product furnished by the BulbE-TE is a 15-atom macrolactam ring (Figure 1B). Expanding or contracting the indolylamide macrocycle, queried using ureidopeptide thioesters **8** and **9** as substrates, respectively, was not successful as only hydrolyzed products were observed in each case (Figure 3, Figures S21–S24).

**Figure 3:**
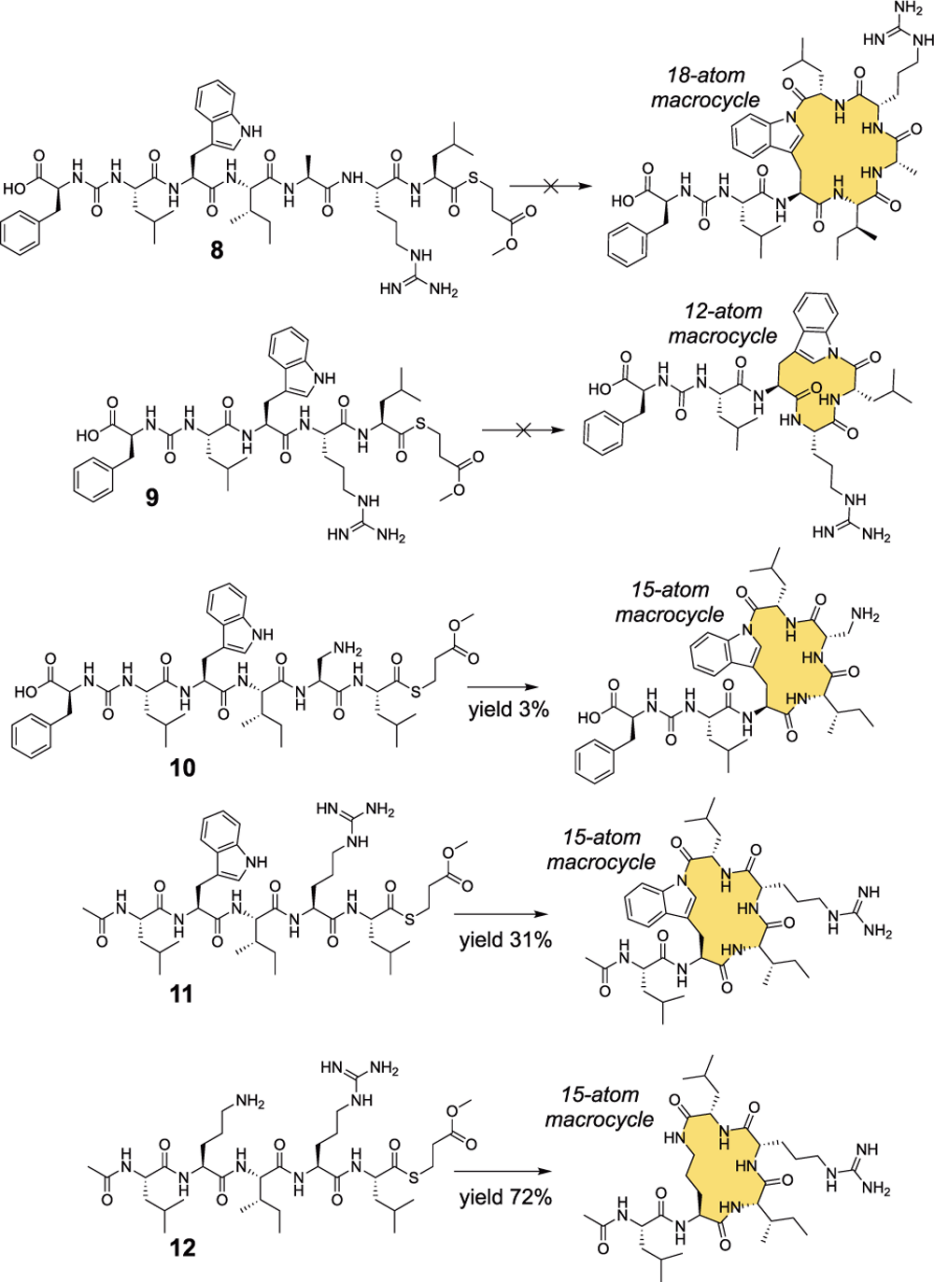
*In vitro* enzymatic biocatalytic potential of BulbE-TE. Substrates **8** and **9** did not yield macrocyclized products. Substrates **10**–**12** did yield macrocyclized peptides demonstrating that the Arg side chain and the ureidopeptide linkage were not required for substrate recognition by the BulbE-TE.

The bulbiferamides demonstrate the invariant presence of the Arg residue as a constituent of the macrolactam ring. Ureidopeptide thioester **10**, wherein the Arg residue was replaced with 1,3-diaminopropionic acid (Dap), was accepted as substrate by the BulbE-TE furnishing the appropriately cyclized macrocyclic product in 3% yield (Figures 3 and S25–S27). However, the thioester hydrolysis product dominated the macrocyclized product (Table S1). The other invariant structural feature in bulbiferamides, the ureido coupling between Phe1 and Leu2 residues, was dispensable with molecule **11** serving as a viable substrate for BulbE-TE, leading to macrocyclic product formation in yields comparable to substrate **1** (Figures 3 and S28–S30). This implies that the ureido group was not required for substrate recognition. Taken together with the fact that other indolylamide-forming enzymes require a CP-loaded substrate, this highlights the ability of the BulbE-TE to serve as a more general biocatalyst. Replacement of the poor indole-N nucleophile in **11** with an ornithine-derived primary amine in molecule **12** yielded an enhanced product yield (Figures 3 and S31–S34, Table S1).

The macrocyclization of **12** mimics the biosynthesis of cyanobacterial ureidopeptidic natural products that are macrocyclized via amide bond formation with Lys side chain primary amines.^26^ Unlike cyclization of **1** and **2**, **12** yielded a macrocyclized product even in the absence of the enzyme which likely alludes to the preorganization of the substrate for intramolecular thioester displacement by a much stronger primary amine nucleophile (Figure S33, Table S1). Decreasing the reaction pH—from 7.5 to 6.0—abolished the noncatalytic product formation and the overall product yield also decreased pointing to the reactivity of the macrocyclizing nucleophile being a primary determinant (Figure S35, Table S1). Increasing the reaction pH—from 7.5 to 9.0—led to thioester hydrolysis being the dominating reaction outcome (Figure S36, Table S1).

The ability of BulbE-TE to acylate the relatively non-nucleophilic tryptophan nitrogen is exciting. Most synthetic strategies for acylation of tryptophan require protection of other nucleophilic residues and cannot happen in nucleophilic solvents such as water.^27,28^ Additionally, they typically require the use of strong, often stoichiometric bases, limiting the functional group tolerance of the reactions. The total synthesis of the fungal macrocyclic indolylamide natural product psychrophilin E has been achieved; the timing for the installation of the indolylamide in the chemical synthesis and in the biosynthetic route is entirely opposite.^29^ While indolylamide installation is the first step in chemical synthesis of psychrophilin E, it is the very last transformation in bulbiferamide biosynthesis. Taken together, BulbE-TE facilitates a synthetically challenging peptide macrocyclization to a 15-membered ring that has not been previously achievable. Future efforts for enzyme evolution are likely to further expand the substrate scope, thus providing a highly useful biocatalyst.

## Data Availability

The data underlying this study are available in the published article and its Supporting Information.

## Supporting Information Available

Comprehensive description of materials and methods used in this study, synthetic schemes, compound characterization data, ^1^H & ^13^C NMR spectra, and descriptions of enzyme reaction outcomes (PDF)

## References

[ref1] Brudy C.; Walz C.; Spiske M.; Dreizler J. K.; Hausch F. The Missing Link(er): A Roadmap to Macrocyclization in Drug Discovery. J. Med. Chem. 2024, 67, 14768–14785. 10.1021/acs.jmedchem.4c01163. 39171975

[ref2] Little R. F.; Hertweck C. Chain Release Mechanisms in Polyketide and Non-ribosomal Peptide Biosynthesis. Nat. Prod. Rep. 2022, 39, 163–205. 10.1039/D1NP00035G. 34622896

[ref3] Horsman M. E.; Hari T. P. A.; Boddy C. N. Polyketide Synthase and Non-ribosomal Peptide Synthetase Thioesterase Selectivity: Logic Gate or a Victim of Fate?. Nat. Prod. Rep. 2016, 33, 183–202. 10.1039/C4NP00148F. 25642666

[ref4] Zhong W.; Deutsch J. M.; Yi D.; Abrahamse N. H.; Mohanty I.; Moore S. G.; McShan A. C.; Garg N.; Agarwal V. Discovery and Biosynthesis of Ureidopeptide Natural Products Macrocyclized via Indole *N*-acylation in Marine *Microbulbifer* spp. Bacteria. ChemBioChem. 2023, 24, e202300190 10.1002/cbic.202300190. 37092875

[ref5] Lu S.; Zhang Z.; Sharma A. R.; Nakajima-Shimada J.; Harunari E.; Oku N.; Trianto A.; Igarashi Y. Bulbiferamide, an Antitrypanosomal Hexapeptide Cyclized via an *N*-acylindole Linkage from a Marine Obligate *Microbulbifer*. J. Nat. Prod. 2023, 86, 1081–1086. 10.1021/acs.jnatprod.2c01083. 36843290

[ref6] Jiao R. H.; Xu S.; Liu J. Y.; Ge H. M.; Ding H.; Xu C.; Zhu H. L.; Tan R. X. Chaetominine, a Cytotoxic Alkaloid Produced by Endophytic *Chaetomium* sp. IFB-E015. Org. Lett. 2006, 8, 5709–5712. 10.1021/ol062257t. 17134253

[ref7] Wong S. M.; Musza L. L.; Kydd G. C.; Kullnig R.; Gillum A. M.; Cooper R. Fiscalins: New Substance P Inhibitors Produced by the Fungus *Neosartorya fischeri*. Taxonomy, Fermentation, Structures, and Biological Properties. J. Antibiot. 1993, 46, 545–553. 10.7164/antibiotics.46.545. 7684734

[ref8] Costa J. H.; Bazioli J. M.; Barbosa L. D.; dos Santos Junior P. L. T.; Reis F. C. G.; Klimeck T.; Crnkovic C. M.; Berlinck R. G. S.; Sussulini A.; Rodrigues M. L.; Fill T. P. Phytotoxic Tryptoquialanines Produced *in vivo* by *Penicillium digitatum* Are Exported in Extracellular Vesicles. mBio 2021, 12, e03393–20. 10.1128/mBio.03393-20. 33563828 PMC7885104

[ref9] Dalsgaard P. W.; Larsen T. O.; Frydenvang K.; Christophersen C. Psychrophilin A and Cycloaspeptide D, Novel Cyclic Peptides from the Psychrotolerant Fungus *Penicillium ribeum*. J. Nat. Prod. 2004, 67, 878–881. 10.1021/np0303714. 15165155

[ref10] Zhao M.; Lin H.-C.; Tang Y. Biosynthesis of the α-Nitro-containing Cyclic Tripeptide Psychrophilin. J. Antibiot. 2016, 69, 571–573. 10.1038/ja.2016.33. PMC4963283 26956794

[ref11] Walsh C. T.; Haynes S. W.; Ames B. D.; Gao X.; Tang Y. Short Pathways to Complexity Generation: Fungal Peptidyl Alkaloid Multicyclic Scaffolds from Anthranilate Building Blocks. ACS Chem. Biol. 2013, 8, 1366–1382. 10.1021/cb4001684. 23659680 PMC3796173

[ref12] Page P.; Bradley M.; Walters I.; Teague S. Solid-phase Synthesis of Tyrosine Peptide Aldehydes. Analogues of (*S*)-MAPI. J. Org. Chem. 1999, 64, 794–799. 10.1021/jo981546v. 11674148

[ref13] Stefanelli S.; Cavaletti L.; Sarubbi E.; Ragg E.; Colombo L.; Selva E. GE20372 Factor A and B. New HIV-1 Protease Inhibitors, Produced by *Streptomyces* sp. ATCC 55925. J. Antibiot. 1995, 48, 332–334. 10.7164/antibiotics.48.332. 7775273

[ref14] Stella S.; Saddler G.; Sarubbi E.; Colombo L.; Stefanelli S.; Denaro M.; Selva E. Isolation of Alpha-MAPI from Fermentation Broths During a Screening Program for HIV-1 Protease Inhibitors. J. Antibiot. 1991, 44, 1019–1022. 10.7164/antibiotics.44.1019. 1938609

[ref15] Budimir Z. L.; Patel R. S.; Eggly A.; Evans C. N.; Rondon-Cordero H. M.; Adams J. J.; Das C.; Parkinson E. I. Biocatalytic Cyclization of Small Macrolactams by a Penicillin-binding Protein-type Thioesterase. Nat. Chem. Biol. 2024, 20, 120–128. 10.1038/s41589-023-01495-z. 38062262 PMC10999230

[ref16] Blanco-Canosa J. B.; Nardone B.; Albericio F.; Dawson P. E. Chemical Protein Synthesis Using a Second-generation *N*-acylurea Linker for the Preparation of Peptide-thioester Precursors. J. Am. Chem. Soc. 2015, 137, 7197–7209. 10.1021/jacs.5b03504. 25978693

[ref17] Tianero M. D.; Pierce E.; Raghuraman S.; Sardar D.; McIntosh J. A.; Heemstra J. R.; Schonrock Z.; Covington B. C.; Maschek J. A.; Cox J. E.; Bachmann B. O.; Olivera B. M.; Ruffner D. E.; Schmidt E. W. Metabolic Model for Diversity-generating Biosynthesis. Proc. Natl. Acad. Sci. U. S. A. 2016, 113, 1772–1777. 10.1073/pnas.1525438113. 26831074 PMC4763782

[ref18] McIntosh J. A.; Robertson C. R.; Agarwal V.; Nair S. K.; Bulaj G. W.; Schmidt E. W. Circular Logic: Nonribosomal Peptide-like Macrocyclization with a Ribosomal Peptide Catalyst. J. Am. Chem. Soc. 2010, 132, 15499–15501. 10.1021/ja1067806. 20961047 PMC2975588

[ref19] Patel K. D.; MacDonald M. R.; Ahmed S. F.; Singh J.; Gulick A. M. Structural Advances toward Understanding the Catalytic Activity and Conformational Dynamics of Modular Nonribosomal Peptide Synthetases. Nat. Prod. Rep. 2023, 40, 1550–1582. 10.1039/D3NP00003F. 37114973 PMC10510592

[ref20] Schaffer J. E.; Reck M. R.; Prasad N. K.; Wencewicz T. A. β-Lactone Formation During Product Release from a Nonribosomal Peptide Synthetase. Nat. Chem. Biol. 2017, 13, 737–744. 10.1038/nchembio.2374. 28504677

[ref21] Oliver R. A.; Li R.; Townsend C. A. Monobactam Formation in Sulfazecin by a Nonribosomal Peptide Synthetase Thioesterase. Nat. Chem. Biol. 2018, 14, 5–7. 10.1038/nchembio.2526. 29155429 PMC5726899

[ref22] Yang W.; Drueckhammer D. G. Understanding the Relative Acyl-transfer Reactivity of Oxoesters and Thioesters: Computational Analysis of Transition State Delocalization Effects. J. Am. Chem. Soc. 2001, 123, 11004–11009. 10.1021/ja010726a. 11686705

[ref23] Weeks A. M.; Wells J. A. Subtiligase-catalyzed Peptide Ligation. Chem. Rev. 2020, 120, 3127–3160. 10.1021/acs.chemrev.9b00372. 31663725

[ref24] Kreitler D. F.; Gemmell E. M.; Schaffer J. E.; Wencewicz T. A.; Gulick A. M. The Structural Basis of *N*-acyl-α-amino-β-lactone Formation Catalyzed by a Nonribosomal Peptide Synthetase. Nat. Commun. 2019, 10, 3432 10.1038/s41467-019-11383-7. 31366889 PMC6668435

[ref25] Patel K. D.; Oliver R. A.; Lichstrahl M. S.; Li R.; Townsend C. A.; Gulick A. M. The Structure of the Monobactam-producing Thioesterase Domain of SulM Forms a Unique Complex with the Upstream Carrier Protein Domain. J. Biol. Chem. 2024, 300, 107489 10.1016/j.jbc.2024.107489. 38908753 PMC11298585

[ref26] Shishido T. K.; Jokela J.; Fewer D. P.; Wahlsten M.; Fiore M. F.; Sivonen K. Simultaneous Production of Anabaenopeptins and Namalides by the Cyanobacterium *Nostoc* sp. CENA543. ACS Chem. Biol. 2017, 12, 2746–2755. 10.1021/acschembio.7b00570. 28933529

[ref27] Heller S. T.; Schultz E. E.; Sarpong R. Chemoselective *N*-acylation of Indoles and Oxazolidinones with Carbonylazoles. Angew. Chem., Int. Ed. 2012, 51, 8304–8308. 10.1002/anie.201203976. 22786619

[ref28] Umehara A.; Shimizu S.; Sasaki M. Synthesis of Bulky *N*-acyl Heterocycles by DMAPO/Boc_2_O-mediated One-pot Direct *N*-acylation of Less Nucleophilic *N*-heterocycles with α-Fully Substituted Carboxylic Acids. Adv. Synth. Catal. 2023, 365, 2367–2376. 10.1002/adsc.202300487.

[ref29] Ngen S. T. Y.; Kaur H.; Hume P. A.; Furkert D. P.; Brimble M. A. Synthesis of Psychrophilin E. J. Org. Chem. 2016, 81, 7635–7643. 10.1021/acs.joc.6b01369. 27442351

