# Supplementary material for: Activity and Biocatalytic Potential of an Indolylamide Generating Thioesterase
Source: Org Lett. 2024 Oct 21;26(43):9378–82. doi: 10.1021/acs.orglett.4c03648 (PMC11536411; doi:10.1021/acs.orglett.4c03648)
Supplement: Supplementary file 1 — ol4c03648_si_001.pdf [file ol4c03648_si_001.pdf]

SUPPLEMENTARY INFORMATION FOR:

Activity and biocatalytic potential of an indolylamide generating thioesterase

Weimao Zhong,[1,†] Zachary L. Budimir,[2,†] Lucas O. Johnson,[2] Elizabeth I. Parkinson,[2,3,]* Vinayak Agarwal[1,4,]*

[1] School of Chemistry and Biochemistry, Georgia Institute of Technology, Atlanta, GA 30332, USA

[2] James Tarpo Jr. and Margaret Tarpo Department of Chemistry, Purdue University, West Lafayette, IN 47907, USA

[3] Borch Department of Medicinal Chemistry and Molecular Pharmacology, Purdue University, West Lafayette, IN 47907, USA

[4] School of Biological Sciences, Georgia Institute of Technology, Atlanta, GA 30332, USA

[†] Equal contribution first authors

* Correspondence: eparkins@purdue.edu and vagarwal@gatech.edu

Table of Contents

SUPPLEMENTARY MATERIALS AND METHODS 3

General materials and instrumentation 3

Cloning, expression and purification of BulbE-TE domains from *Microbulbifer* sp. MLAF003 and *Microbulbifer* sp. VAAF005 3

Amino acid sequences of the BulbE-TE and BulbE-TE2 4

Enzymatic assays 4

Kinetic characterization of BulbE-TE activity 5

Synthesis of Urea Dipeptide 5

Conventional peptide thioester synthesis at the NDbz "safety-catch" resin 8

Peptide Thioesters Used in This Study 10

SUPPLEMENTARY TABLES 24

Table S1: Enzymatic conversion yields for cyclic and hydrolysis products by the two BulbE-TE enzymes, C961S BulbE-TE, and no enzyme control 24

SUPPLEMENTARY FIGURES 25

SUPPLEMENTARY REFERENCES 62

Supplementary Materials and Methods

General materials and instrumentation

All chemicals, solvents, and media components were obtained commercially from Sigma-Aldrich, Fisher Scientific, and VWR, and used without further purification. Phusion high-fidelity DNA polymerase and Gibson assembly Master Mix were purchased from New England Biolabs. PrimeSTAR DNA polymerase Master Mix was purchased from Takaro Bio. Mass spectra were recorded on an Agilent 6530C high resolution time of flight (ToF) mass spectrometer with an electrospray ionization (ESI) source coupled to an Agilent 1260 high-performance liquid chromatography system equipped with a diode array detector. NMR data were recorded on a Bruker Avance-III-800 with a QCI cryoprobe or a Bruker NEO500 with a BBFO cryoprobe.

Cloning, expression and purification of BulbE-TE domains from *Microbulbifer* sp. MLAF003 and *Microbulbifer* sp. VAAF005

On the basis of primary protein sequence similarity, the domain boundaries for BulbE-TE domain were mapped to *Microbulbifer* sp. MLAF003 BulbE residues 866–1135. The DNA fragment encoding TE domain was amplified from the genomic DNA of *Microbulbifer* sp. MLAF003 with Phusion high fidelity DNA polymerase. The amplified DNA fragment was inserted into the pET28(+) vector using Gibson assembly to furnish an N-His_6 tag fused construct. The sequence of the plasmid was confirmed by nanopore sequencing. The plasmid was then transformed into *Escherichia coli* BL21Gold(DE3) for protein expression.

Overnight culture was inoculated into 1 L terrific broth supplemented with 50 µg/mL kanamycin. The cells were grown at 30 ˚C until OD_{600} reached 0.4–0.5. The incubation temperature was then reduced to 18 ˚C. At an OD_{600} of 0.7–0.8, protein expression was induced by the addition of 0.1 mM IPTG. Induced cultures were allowed to grow at 18 ˚C for an addition of 18 h before being harvested by centrifugation. Cell pellets were resuspended in 50 mL binding buffer containing 20 mM Tris-HCl (pH 8.0), 500 mM NaCl, and lysed by homogenization. The lysate was clarified by centrifugation (40,000 × g, 45 min), and then applied to a 5 mL HisTrap HP column. The column was washed with 10 column volumes of wash buffer (20 mM Tris-HCl (pH 8.0), 500 mM NaCl, 30 mM imidazole), and eluted using ÄKTAprime plus FPLC system with a linear gradient to 100% of elution buffer (20 mM Tris-HCl (pH 8.0), 250 mM imidazole, 500 mM NaCl) over 6 column volumes. Purity of eluent protein fractions was checked by SDS-PAGE. The fractions containing desired proteins were pooled and dialyzed in 2 L binding buffer overnight. Freshly prepared proteins were used for assays. The BulbE-TE from *Microbulbifer* sp. VAAF005 strain

(referred to as BulbE-TE2 in some of the figures below) was prepared in an identical manner. The sequences for the two BulbE-TE domains are listed below.

Amino acid sequences of the BulbE-TE and BulbE-TE2

BulbE-TE from *Microbulbifer* sp. MLAF003

MENEIVDFLKDYNEFRKFMTFNGDSNKTGLFLIPAAAGPETFIPLVEKLDIDRPVQLLENIQVYSG
RQIRLNHLIDYYLAVIRKKQPGGPYFLGGYCEGAMVSLGIAQKLEALGEQVEMLFLIDPVVITIEQ
TMIDTIKQDSRLLECGRFEAEMVDTFLFYAEYVKSLHPYGGPAIFFEGSSVSDEATPTQTLALIND
YIDIQGVFKKGFSTPKNGFEDLLLNCDYISIKAKHERVMIEDETLNTIAMAINRKLSTGQQTYLAP
EAQTTEM

BulbE-TE2 from *Microbulbifer* sp. VAAF005

MDEIVNFLTGYNEFNKLMTFNEASNKTGLFLIPAAAGPETFTPLVEKLDINRPVHLLENIQVYSGR
QIRLNYLIDYYFAVIRKKQASGPYLLGGYCEGAMVSLGIAQKLIALGERVELLFLIDPVVITIEQNL
IDTIKQDPRLPKCGRFEAEMVDTFLFYAEYVKSLQPYSGPVVFFEGSSVSDEATPTQILAVINDYV
DIQELFNKGFSTPKNGFESLLLNCDYIAIDAKHERIMIEDETLNTMANVINLKFSSSYDNYLETETH
S

Enzymatic assays

Enzymatic assays were performed at 30 ˚C for 12 h, in a total volumn of 100 μL containing 20 mM Tris-Cl (pH 7.5), 100 mM NaCl, 1 mM DTT, 100 μM substrates, 1 μM wild type or mutated BulbE-TE enzymes. Additionally, substrate **12** was also tested in buffers at pH 6.0 and 9.0. Reactions were quenched by addition of equal volume of MeOH supplemented with 2% (v/v) formic acid (FA). Negative control reactions omitted the enzyme. Quenched aliquots were centrifuged at 18,000×g for 30 min at room temperature before analysis by LCMS. Chromatographic separations were performed using an Agilent Poroshell 120 EC-C_{18} reverse phase HPLC column (100 × 4.6 mm, 5.0 μm) at a flow rate of 0.3 mL/min. The mobile phase was composed of H_2O (A) and MeCN (B) both supplemented with 0.1% (v/v) FA. A flow rate of 0.3 mL/min was used with the following gradient: 0–3 min: 5% B, 3–15 min: linear gradient to 100% B, 15–18 min: 100% B, 18–20 min: linear gradient to 5% B, 20–22 min: 5% B. Data were acquired in the positive ionization mode with *m/z* 100–3000 Da.

Kinetic characterization of BulbE-TE activity

The time-course experiments for BulbE-TE (from *Microbulbifer* sp. MLAF003) kinetics were performed at 30 ˚C for 12 h in a total volume of 200 µL containing 100 µM substrate **1**, 1 µM BulbE-TE enzyme, 20 mM Tris-Cl (pH 7.5), 100 mM NaCl, and 1 mM DTT. At 1, 5, 10, 30, 60, 120, 240, 480, 960 min, a 20 µL aliquot of enzyme assay was withdrawn, quenched by the addition of 20 µL MeOH with 2% formic acid and analyzed by HPLC to determine the linear response time for the discontinuous assay. Assays with different concentrations (10, 25, 50, 100, 200, 500 µM) of substrate **1** were then conducted at 30 ˚C for 10 min. The initial velocity of product formation was calculated. The resulting curve was fit using OriginPro 2018 (9.5) to extract K_M and k_{cat}.

Synthesis of Urea Dipeptide

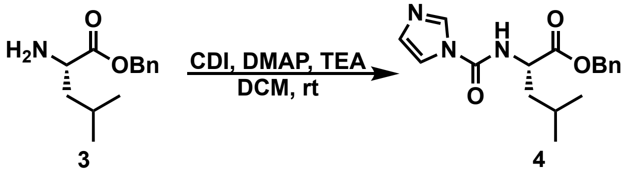

In a flame-dried round-bottom flask, L-leucine benzyl ester p-toluenesulfonate **3** (5 g, 12.7 mmol, 1 equiv.) was dissolved in dry CH_2Cl_2 (50 mL) and cooled to 0 °C. Triethylamine (TEA, 5.2 mL, 38 mmol, 3 equiv.) and 4-dimethylaminopyridine (DMAP, 61 mg, 0.51 mmol, 0.04 equiv.) were added. After stirring for 5 min, carbonyldiimidazole (CDI, 2.3 g, 14 mmol, 1.1 equiv.) was added, and the mixture was stirred overnight while warming to room temperature. The reaction was then diluted with an additional 50 mL of CH_2Cl_2. The organic layer was washed with sodium bicarbonate (2 × 50 mL), dH_2O (1 × 50 mL), and brine (3 × 50 mL). It was then dried over sodium sulfate and concentrated in vacuo to afford **4** as a light yellow oil. The reaction product (3.6 g, 11.4 mmol) was verified by proton NMR and used crude in the next reaction.

^{1}H NMR (500 MHz, DMSO-d_6): δ = 8.80 (d, J = 7.6, 1H), 8.27 (d, J = 1.2, 1H), 7.71 (q, J = 1.4, 1H), 7.45–7.29 (m, 5H), 7.03 (s, 1H), 5.16 (s, 2H), 4.42 (ddd, J = 10.9, 7.6, 4.5, 1H), 1.81–1.55 (m, 3H), 0.89 (m, 6H).

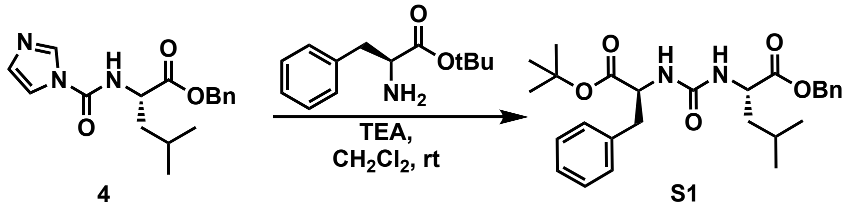

In a flame-dried round-bottom flask, L-phenylalanine tert-butyl ester hydrochloride (3.5 g, 13.7 mmol, 1.2 equiv.) was suspended in dry CH_2Cl_2 (40 mL). Compound **4** (3.6 g, 11.4 mmol) was dissolved in 10 mL of dry CH_2Cl_2 and added to the flask. The mixture was cooled to 0 °C, and TEA (3.9 mL, 28.5 mmol, 2.5 equiv.) was added. The reaction mixture was stirred overnight while warming to room temperature. Upon completion, the reaction was diluted with an additional 50 mL of CH_2Cl_2. The organic layer was washed with sodium bicarbonate (2 × 50 mL), dH_2O (1 × 50 mL), and brine (3 × 50 mL). It was then dried over sodium sulfate, concentrated in vacuo, and purified by flash chromatography (30% EtOAc/Hexanes) to give the diester **S1** as a clear, viscous oil (4.4 g, 9.5 mmol, 83%). Rf = 0.50 (30% EtOAc/Hexanes).

^{1}H NMR (800 MHz, DMSO-d_6): δ = 7.39–7.30 (m, 5H), 7.27 (t, J = 7.5, 2H), 7.24–7.16 (m, 3H), 6.58 (d, J = 8.2, 1H), 6.19 (d, J = 8.1, 1H), 5.11 (d, J = 3.1, 2H), 4.28 (q, J = 7.0, 1H), 4.21 (td, J = 8.5, 6.0, 1H), 2.90 (d, J = 6.7, 2H), 1.62 (m, 1H), 1.50–1.42 (m, 2H), 1.32 (s, 9H), 0.86 (m, 6H).

^{13}C NMR (201 MHz, DMSO-d_6): δ = 173.7, 171.7, 157.29, 137.4, 136.4, 129.7, 128.8, 128.5, 128.4, 128.19, 126.9, 80.9, 79.6, 66.1, 54.9, 51.4, 41.1, 38.2, 27.9, 24.6, 23.1, 21.9.

Mass spec: HRMS (ESI-TOF) *m/z*: $[M + H]^+$ Calcd for $C_{27}H_{37}N_2O_5$ 469.2697; Found 469.2640.

UPLC Trace: Obtained using mobile phases of H_2O + 0.1% formic acid (A) and acetonitrile + 0.1% formic acid (B). Samples were eluted using a gradient mode with mobile phase B ranging from 5% to 95% over 5 min at a flow rate of 0.5 mL/min. The column was equilibrated with 5% mobile phase B for 1 min before and 2 min after the gradient. Monitoring at wavelength of 254 nm.

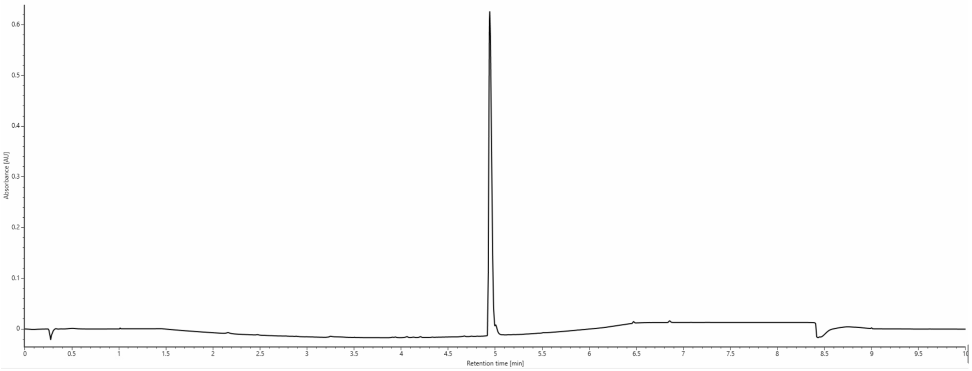

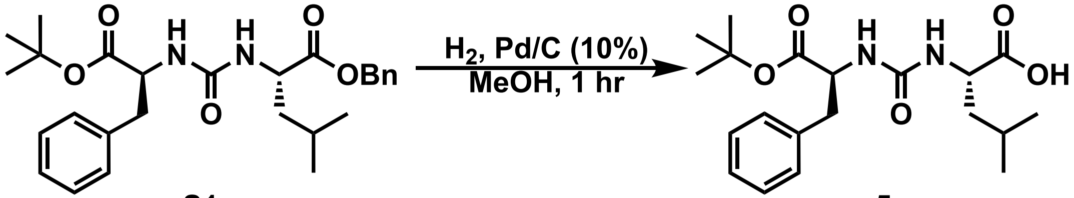

S1 5

The protected urea diester **S1** (5.3 g, 11.3 mmol) was dissolved in methanol and sparged with N_2 (2 × 10 min) and 110 mg Pd-C (10 mol%) was added. The solution was then sparged with H_2 (10 min) and left to hydrogenate for 1 h. The reaction mixture was then filtered over celite and concentrated in vacuo to give **5** as a crystalline white solid (2.7 g, 7.1 mmol, 63%) which was deemed sufficiently pure to use for SPPS without additional purificaiton.

^{1}H NMR (800 MHz, DMSO-*d*$_6$): δ = 7.24 (m, 5H), 6.41 (s, 1H), 6.19 (s, 1H), 4.26 (s, 1H), 4.08 (s, 1H), 2.89 (s, 2H), 1.64 (s, 1H), 1.32 (s, 11H), 0.89 (s, 6H).

^{13}C NMR (201 MHz, DMSO-*d*$_6$): δ = 175.5, 171.8, 157.4, 137.4, 129.7, 128.5, 126.9, 80.8, 55.0, 51.2, 41.6, 38.1, 27.9, 24.6, 23.3, 21.9.

Mass spec: HRMS (ESI-TOF) *m/z*: $[M + H]^+$ Calcd for $C_{20}H_{31}N_2O_5$ 379.2227; Found 379.2170.

UPLC Trace: Obtained using mobile phases of H_2O + 0.1% formic acid (A) and acetonitrile + 0.1% formic acid (B). Samples were eluted using a gradient mode with mobile phase B ranging from 5% to 95% over 5 min at a flow rate of 0.5 mL/min. The column was equilibrated with 5% mobile phase B for 1 min before and 2 min after the gradient. Monitoring at wavelength of 254 nm.

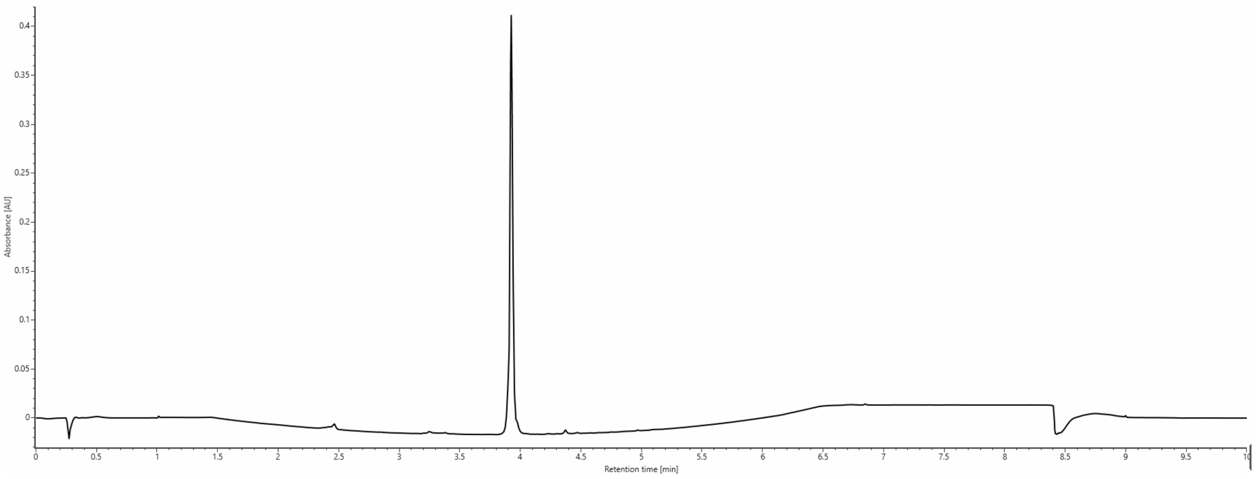

Conventional peptide thioester synthesis at the NDbz "safety-catch" resin

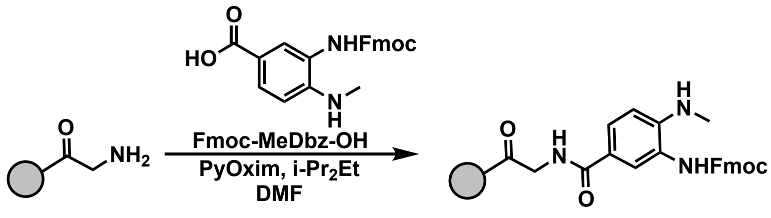

S2 S3

Attachment of Fmoc-MeDbz-OH: A 5 mL fritted polypropylene syringe (Torviq) containing 0.1 mmol of Fmoc-Gly Rink amide resin (0.592 mmol/g loading) **S2** (Chem-Impex, MFCD00801253) was washed with DMF (3 × 5 mL) and allowed to swell for 15 min. A solution of Fmoc-MeDbz-OH (synthesized according to a previously published route)[1] (0.3 mmol), Pyoxim (0.5 mmol), and diisopropylethylamine (DIPEA, 0.7 mmol) in 3 mL of DMF was then added to the resin and allowed to shake for 1 h. The resin was filtered and washed with CH_2Cl_2 (3 × 5 mL) and DMF (3 × 5 mL). Loading efficiency was assumed to be 100% based on a negative ninhydrin test, and the resin was subsequently used in solid-phase peptide synthesis.

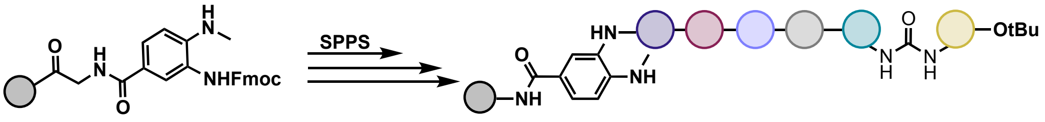

Solid-phase peptide synthesis (SPPS): All manual SPPS and cleavage steps were carried out using 5 mL fritted polypropylene syringes (Torviq) as reaction vessels. Pre-loaded Fmoc-MeDbz resin (0.1 mmol) was swelled in DMF for 15 min, drained, and treated with piperidine-DMF (1:4, 4 mL, 1 × 15 min). The resin was then filtered and washed with DMF (2 × 3 mL) and CH_2Cl_2 (2 × 3 mL). In a separate flask, Pyoxim (0.5 mmol) was added to a solution of Fmoc-AA-OH (0.5 mmol) and diisopropylethylamine (0.7

mmol) in DMF (3 mL). The resulting solution was added to the resin, and the mixture was agitated for 1 h. The resin was then filtered and washed with DMF (3 × 2 mL) and CH_2Cl_2 (3 × 2 mL). The Kaiser ninhydrin test was performed to determine reaction completion. Deprotection and coupling cycles were repeated until the desired peptide sequence was complete. The final protected urea dipeptide **5** (0.3 mmol) was attached following the same procedure as above with a lower equivalents of peptide used.

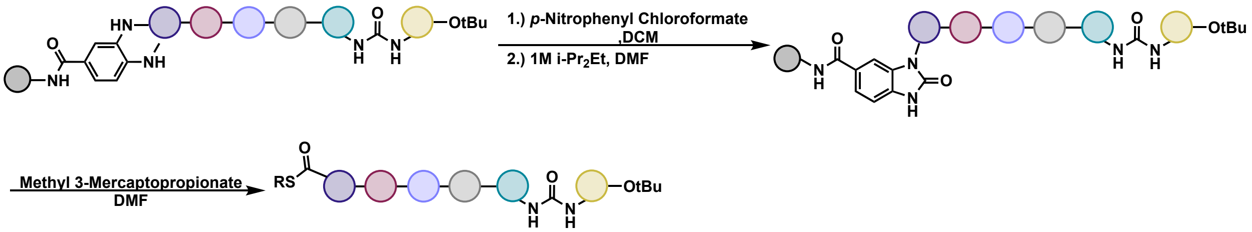

Activation and thiolysis: The resin was washed with CH_2Cl_2 (3 × 5 mL) and allowed to swell for 15 min. The resin was then treated with 1 mL of 0.5 M 4-nitrophenyl chloroformate (TCI America) in CH_2Cl_2 and shaken for 2 h. The resin was subsequently washed with CH_2Cl_2 (3 × 5 mL) and DMF (3 × 5 mL). A solution of 3 mL of 0.5 M DIPEA in DMF was added to the resin and allowed to react for 15 min. The resin was then filtered, washed with DMF (1 × 5 mL), and exposed to 3 mL fresh 0.5 M DIPEA in DMF solution, repeating this process until the solution no longer turned yellow (typically 4 times). The resin was then swollen in 3 mL DMF for 15 min, filtered, and treated with a 50:50 solution of thiol (2.5 mmol) in DMF, and shaken for 24 h. The resin was then filtered and washed with DMF (3 × 1 mL). The combined filtrate and washes were collected in a 20 mL scintillation vial and subjected to rotary evaporation and lyophilization.

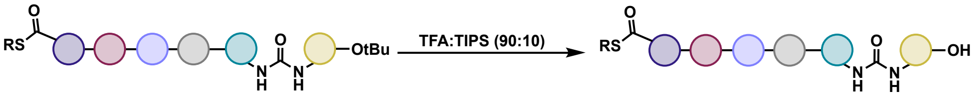

Global deprotection of peptide thioesters: The crude peptide was subjected to global deprotection by treating it with a mixture of 5 mL trifluoroacetic acid (TFA) and triisopropylsilane (TIPS) in a ratio of 90:10 for 1.5 h. The TFA solution was then removed by a stream of air, and the peptide was precipitated by addition of diethyl ether. The resulting precipitate was collected by centrifugation, and the supernatant was discarded. The peptide was then lyophilized to obtain a dry powder. The crude peptide was purified via reverse-phase semi-preparative HPLC using mobile phases of H_2O with 0.05% TFA (A) and acetonitrile with 0.05% TFA (B). Samples were eluted using a gradient mode with mobile phase B ranging from 0% to 70% over 22 min at a flow rate of 20 mL/min. The purified product was then characterized by ultra-performance liquid chromatography-mass spectrometry (UPLC-MS) to ensure a purity >95% (UV 214 nm).

Peptide Thioesters Used in This Study

In this study, UPLC and/or analytical HPLC were utilized to assess the purity of the peptide prior to assay or purification by monitoring absorbance at 214 nm. The analytical HPLC analysis was performed on a Luna Omega 5 µm Polar C_{18} 100 Å 150 × 4.6 mm (Phenomenex) column, while the UPLC analysis was carried out on a CORTECS T3 Column, 120 Å, 1.6 µm, 2.1 mm × 50 mm (Waters) column. For purification, semi-preparative HPLC was employed using a Luna Omega 5 µm Polar C_{18} 100 Å 150 × 21.2 mm (Phenomenex) column. The specific gradient and flow rate for each peptide can be found in their respective sections.

Synthesis of 1

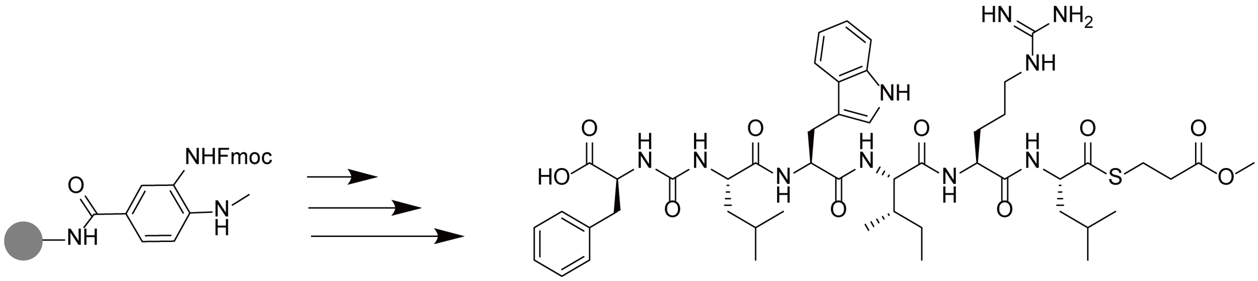

S3 **1**

Peptide **1** was synthesized using the NDbz "safety-catch" resin. The synthesis was initiated from Fmoc-MeDbz-OH **S3** loaded resin (0.10 mmol), which was then coupled with the following amino acids: Fmoc-L-Leu-OH, Fmoc-L-Arg(Pbf)-OH, Fmoc-L-Ile-OH, Fmoc-L-Trp(Boc)-OH, and **5**. Methyl 3-mercaptopropionate was used as the cleaving thiol. The crude peptide was purified via reverse-phase semi-preparative HPLC using mobile phases of acetonitrile + 0.05% TFA (B) and H_2O + 0.05% TFA (A). Samples were eluted using a gradient mode with mobile phase B ranging from 0% to 70% over 22 min at a flow rate of 20 mL/min. The column was equilibrated with 0% mobile phase B for 5 min after and 1 min before the gradient to yield the TFA salt of **1** as an off-white solid (45 mg, 43% yield).

^{1}H NMR (800 MHz, DMSO-d_6): δ = 10.76 (d, J = 2.5, 1H), 8.52 (d, J = 7.8, 1H), 8.13–8.00 (m, 2H), 7.73 (d, J = 8.5, 1H), 7.64 (d, J = 6.0, 1H), 7.54 (d, J = 7.9, 1H), 7.31 (d, J = 8.1, 1H), 7.26 (t, J = 7.5, 2H), 7.22–7.14 (m, 3H), 7.11 (d, J = 2.4, 1H), 7.08–7.02 (m, 1H), 6.95 (t, J = 7.2, 1H), 6.35 (d, J = 8.0, 1H), 6.20 (d, J = 8.1, 1H), 4.59 (td, J = 8.0, 5.1, 1H), 4.43–4.29 (m, 3H), 4.26–4.20 (m, 1H), 4.14–4.07 (m, 1H), 3.61 (s, 3H), 3.13 (tt, J = 12.5, 5.9, 3H), 2.98 (dtd, J = 13.5, 7.6, 4.1, 4H), 2.56 (t, J = 7.1, 2H), 1.81–1.73 (m, 1H),

1.73–1.61 (m, 2H), 1.63–1.45 (m, 6H), 1.43–1.29 (m, 2H), 1.25 (ddd, J = 14.3, 9.9, 5.5, 1H), 1.05 (ddt, J = 16.0, 8.9, 5.1, 1H), 0.92–0.66 (m, 19H).

^{13}C NMR (201 MHz, DMSO-d_6): δ = 206.9, 201.7, 174.1, 173.4, 172.1, 172.0, 171.5, 171.0, 157.5, 157.2, 137.7, 136.4, 129.7, 128.6, 127.8, 126.8, 123.7, 121.2, 118.7, 118.6, 111.6, 110.4, 57.8, 57.1, 54.3, 53.5, 52.4, 52.0, 52.0, 42.5, 40.8, 37.9, 37.4, 33.8, 31.1, 29.2, 27.4, 25.5, 24.5, 24.4, 24.4, 23.7, 23.5, 23.3, 22.2, 21.2, 15.5, 11.5.

Mass spec: HRMS (ESI-TOF) *m/z*: $[M + H]^+$ Calcd for $C_{49}H_{73}N_{10}O_{10}S$ 993.5226; Found 993.5132.

UPLC Trace Obtained using mobile phases of H_2O + 0.1% formic acid (A) and acetonitrile + 0.1% formic acid (B). Samples were eluted using a gradient mode with mobile phase B ranging from 0% to 70% over 6 min at a flow rate of 0.5 mL/min. The column was equilibrated with 0% mobile phase B for 1 min before and 2 min after the gradient. The peptide purity was determined to be 98% monitoring at wavelength of 214 nm.

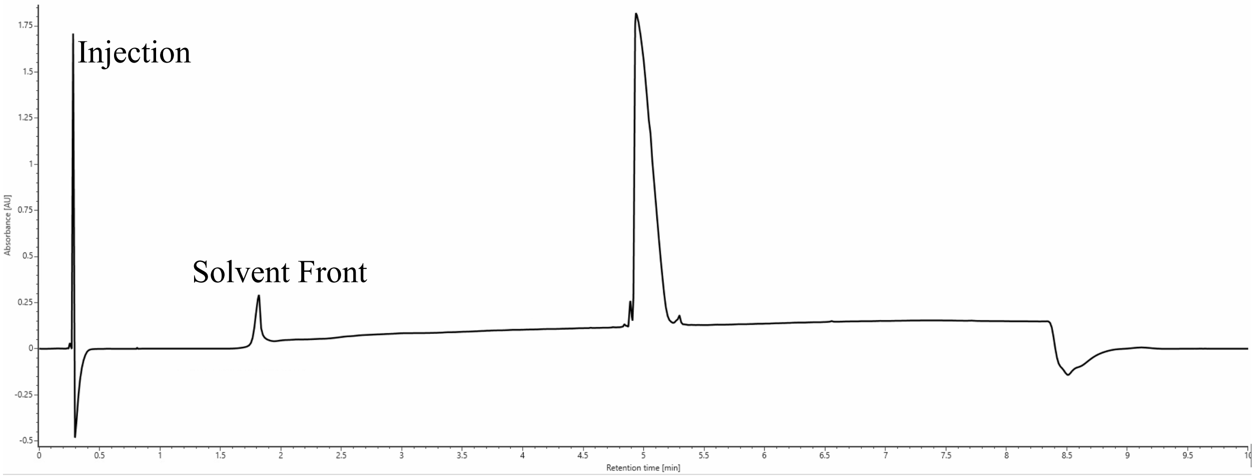

Synthesis of 2

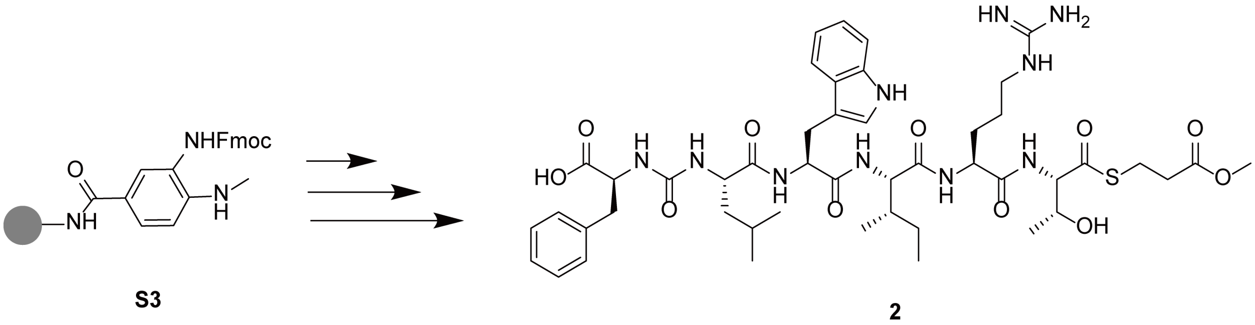

Peptide **2** was synthesized using the NDbz "safety-catch" resin. The synthesis was initiated from Fmoc-MeDbz-OH **S3** loaded resin (0.10 mmol), which was coupled to the following amino acids: Fmoc-L-Thr(tBu)-OH, Fmoc-L-Arg(Pbf)-OH, Fmoc-L-Ile-OH, Fmoc-L-Trp(Boc)-OH, and **5**. Methyl 3-mercaptopropionate was used as the cleaving thiol. The crude peptide was purified via reverse-phase semi-preparative HPLC using mobile phases of acetonitrile + 0.05% TFA (B) and H_2O + 0.05% TFA (A). Samples were eluted using a gradient mode with mobile phase B ranging from 0% to 70% over 22 min at a flow rate of 20 mL/min. The column was equilibrated with 0% mobile phase B for 5 min after and 1 min before the gradient to yield the TFA salt of **2** an off-white solid (16 mg, 15% yield).

^{1}H NMR (800 MHz, DMSO-d_6): δ = 10.76 (d, J = 2.4, 1H), 8.23 (d, J = 8.6, 1H), 8.11 (d, J = 7.8, 1H), 8.05 (d, J = 7.9, 1H), 7.77 (d, J = 8.6, 1H), 7.53 (d, J = 8.0, 1H), 7.30 (t, J = 6.1, 1H), 7.21 (s, 2H), 7.18 (dd, J = 14.5, 7.3, 4H), 7.10 (d, J = 2.3, 2H), 7.04 (t, J = 7.5, 2H), 6.94 (q, J = 7.2, 2H), 6.34 (d, J = 8.1, 1H), 6.18 (d, J = 8.1, 1H), 5.08 (d, J = 4.9, 1H), 4.56 (m, 2H), 4.38–3.98 (m, 5H), 3.61 (d, J = 11.3, 4H), 3.36 (s, 8H), 3.19–3.03 (m, 4H), 2.75–3.03 (m, 7H), 1.85–1.73 (m, 1H), 1.70 (dtd, J = 10.1, 6.9, 3.4, 1H), 1.63–1.45 (m, 5H), 1.24 (ddd, J = 14.3, 9.8, 5.4, 1H), 1.12–0.95 (m, 4H), 0.79 (m, 14H).

^{13}C NMR (201 MHz, DMSO-d_6): δ = 200.5, 174.2, 173.4, 172.7, 172.0, 171.5, 171.2, 157.5, 157.2, 137.7, 136.4, 129.7, 128.6, 127.8, 126.8, 123.7, 121.2, 118.8, 118.6, 111.6, 110.4, 66.5, 64.8, 57.2, 54.4, 53.4, 52.3, 51.9, 42.5, 40.8, 37.9, 37.3, 33.8, 33.6, 33.0, 29.2, 27.4, 25.4, 24.5, 24.4, 23.8, 23.5, 22.2, 20.5, 15.6, 11.5.

Mass spec: HRMS (ESI-TOF) *m/z*: $[M + H]^+$ Calcd for $C_{47}H_{69}N_{10}O_{11}S$ 981.4863; Found 981.4830.

UPLC Trace Obtained using mobile phases of H_2O + 0.1% formic acid (A) and acetonitrile + 0.1% formic acid (B). Samples were eluted using a gradient mode with mobile phase B ranging from 0% to 70% over 4 min at a flow rate of 0.5 mL/min. The column was equilibrated with 0% mobile phase B for 1 min before and 2 min after the gradient. The peptide purity was determined to be >99% monitoring at wavelength of 214 nm.

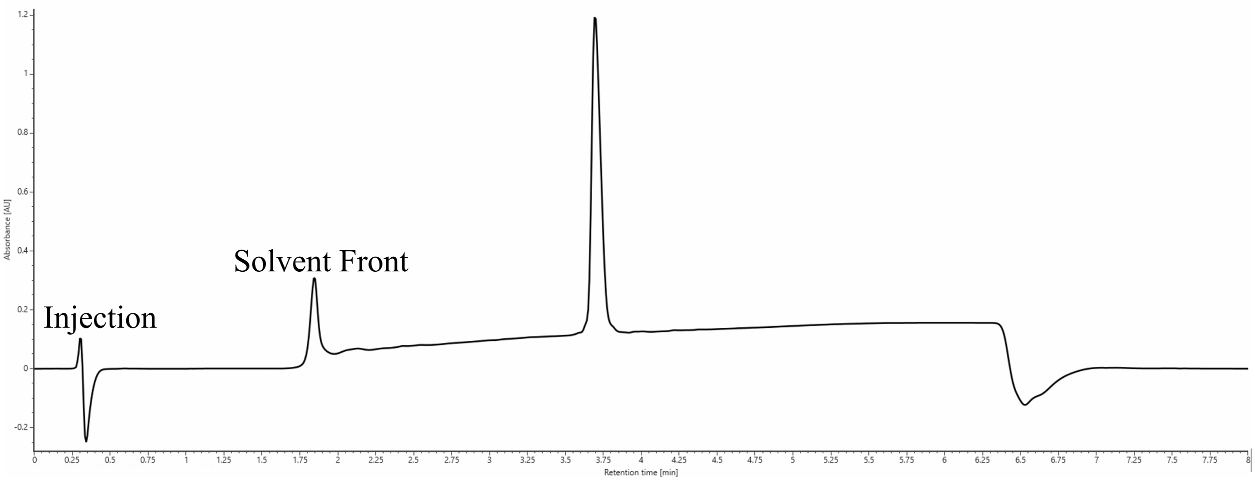

Injection
Solvent Front
Absorbance [AU]
Retention time [min]

Synthesis of 6

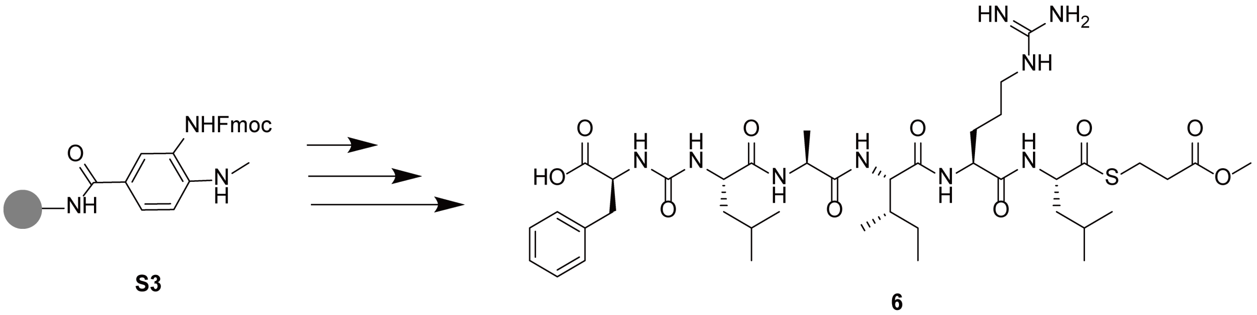

Peptide **6** was synthesized using the NDbz "safety-catch" resin. The synthesis was initiated from Fmoc-MeDbz-OH **S3** loaded resin (0.10 mmol), which was then coupled with the following amino acids: Fmoc-L-Leu-OH, Fmoc-L-Arg(Pbf)-OH, Fmoc-L-Ile-OH, Fmoc-L-Ala-OH, and **5**. Methyl 3-mercaptopropionate was used as the cleaving thiol. The crude peptide was purified via reverse-phase semi-preparative HPLC using mobile phases of acetonitrile + 0.05% TFA (B) and H_2O + 0.05% TFA (A). Samples were eluted using a gradient mode with mobile phase B ranging from 0% to 70% over 22 min at a flow rate of 20 mL/min. The column was equilibrated with 0% mobile phase B for 5 min after and 1 min before the gradient to yield the TFA salt of **6** an off-white solid (36 mg, 36% yield).

^{1}H NMR (800 MHz, DMSO-d_6): δ = 8.51 (s, 1H), 8.08 (d, J = 6.6, 2H), 7.74 (s, 1H), 7.63 (s, 1H), 7.27 (s, 3H), 7.20 (s, 4H), 6.36 (s, 1H), 6.21 (s, 1H), 4.46–4.25 (m, 5H), 4.19 (s, 1H), 4.12 (s, 1H), 3.61 (s, 4H), 3.10 (s, 2H), 2.98 (s, 4H), 2.87 (s, 1H), 2.56 (s, 3H), 1.81–1.43 (m, 11H), 1.34 (m, 4H), 1.17 (s, 4H), 1.05 (s, 1H), 0.96–0.73 (m, 23H).

^{13}C NMR (201 MHz, DMSO-d_6): δ = 201.8, 174.4, 173.4, 172.4, 172.1, 172.0, 171.2, 157.7, 157.3, 138.0, 129.7, 128.6, 126.8, 57.9, 57.1, 54.6, 52.4, 52.0, 51.9, 48.6, 42.5, 40.9, 38.0, 37.3, 33.9, 29.3, 25.6, 24.6, 24.5, 24.4, 23.7, 23.6, 23.4, 22.3, 21.3, 18.0, 15.7, 11.6.

Mass spec: HRMS (ESI-TOF) *m/z*: $[M + H]^+$ Calcd for $C_{41}H_{68}N_9O_{10}S$ 878.4804; Found 878.4862

UPLC Trace Obtained using mobile phases of H_2O + 0.1% formic acid (A) and acetonitrile + 0.1% formic acid (B). Samples were eluted using a gradient mode with mobile phase B ranging from 0% to 70% over 6 min at a flow rate of 0.5 mL/min. The column was equilibrated with 0% mobile phase B for 1 min before and 2 min after the gradient. The peptide purity was determined to be 95%.

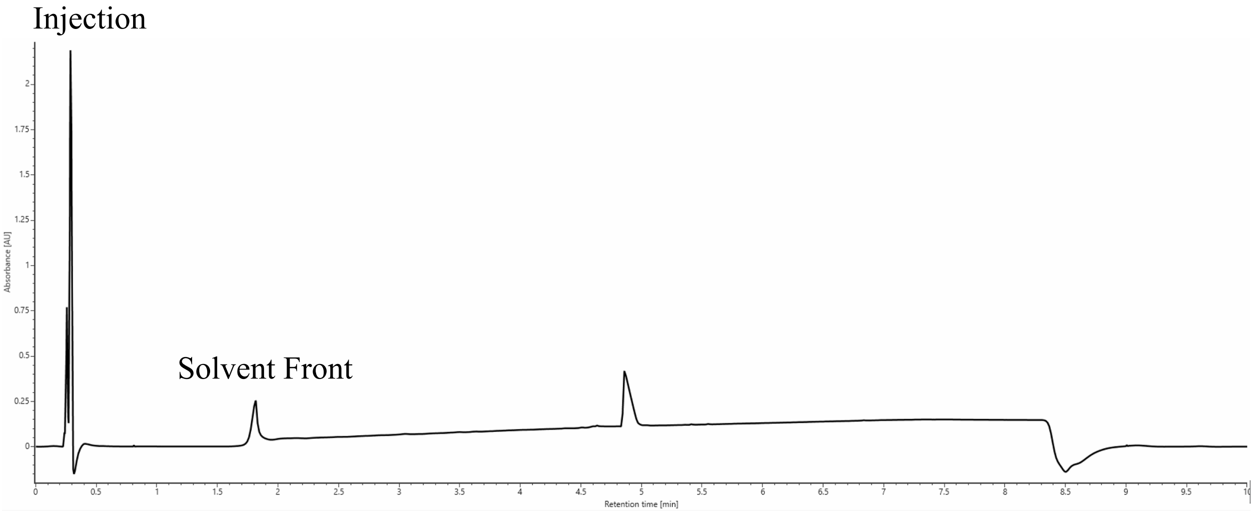

Synthesis of 7

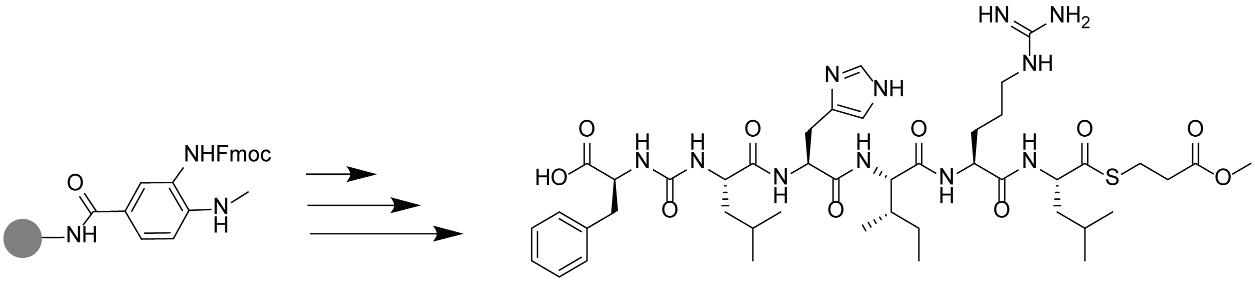

Peptide **7** was synthesized using the NDbz "safety-catch" resin. The synthesis was initiated from Fmoc-MeDbz-OH **S3** loaded resin (0.10 mmol), which was then coupled with the following amino acids: Fmoc-L-Leu-OH, Fmoc-L-Arg(Pbf)-OH, Fmoc-L-Ile-OH, Fmoc-L-His(Trt)-OH, and **5**. Methyl 3-mercaptopropionate was used as the cleaving thiol. The crude peptide was purified via reverse-phase semi-preparative HPLC using mobile phases of acetonitrile + 0.05% TFA (B) and H_2O + 0.05% TFA (A). Samples were eluted using a gradient mode with mobile phase B ranging from 0% to 70% over 22 min at a flow rate of 20 mL/min. The column was equilibrated with 0% mobile phase B for 5 min after and 1 min before the gradient to yield the TFA salt of **7** as an off-white solid (28 mg, 26% yield).

^{1}H NMR (800 MHz, DMSO-d_6): δ = 8.51 (s, 1H), 8.34 (s, 1H), 8.21–8.15 (m, 1H), 7.76 (t, J = 5.8, 1H), 7.66 (d, J = 8.3, 1H), 7.27 (t, J = 7.5, 3H), 7.19 (dt, J = 13.7, 6.6, 5H), 6.45 (d, J = 7.4, 1H), 6.26 (d, J = 8.1, 1H), 4.57 (q, J = 7.3, 1H), 4.37 (ddd, J = 11.4, 7.7, 4.3, 1H), 4.36–4.30 (m, 2H), 4.20 (t, J = 7.4, 1H), 4.05 (h, J = 5.8, 1H), 3.60 (s, 4H), 3.11 (q, J = 7.3, 2H), 2.97 (s, 7H), 2.55 (t, J = 7.1, 2H), 2.52–2.48 (m,

3H), 1.75 (dt, J = 16.9, 6.6, 1H), 1.69 (dtd, J = 10.2, 6.7, 3.4, 1H), 1.66–1.46 (m, 8H), 1.33 (dtd, J = 17.7, 10.8, 4.6, 4H), 1.06–0.98 (m, 1H), 0.92–0.84 (m, 7H), 0.83 (d, J = 6.5, 4H), 0.81–0.73 (m, 11H).

^{13}C NMR (201 MHz, DMSO-d_6): δ = 201.8, 174.2, 173.8, 172.1, 172.0, 171.2, 170.4, 157.9, 157.3, 137.8, 134.4, 129.8, 128.7, 126.9, 118.4, 116.9, 57.9, 57.2, 54.4, 52.5, 52.3, 52.2, 52.0, 42.0, 40.9, 38.0, 37.4, 33.9, 29.2, 27.5, 25.6, 24.5, 24.5, 24.4, 23.7, 23.5, 23.4, 22.2, 21.3, 15.6, 11.6.

Mass spec: HRMS (ESI-TOF) *m/z*: $[M + H]^+$ Calcd for $C_{44}H_{70}N_{11}O_{10}S$ 944.5022; Found 944.5183.

UPLC Trace Obtained using mobile phases of H_2O + 0.1% formic acid (A) and acetonitrile + 0.1% formic acid (B). Samples were eluted using a gradient mode with mobile phase B ranging from 0% to 70% over 4 min at a flow rate of 0.5 mL/min. The column was equilibrated with 0% mobile phase B for 1 min before and 2 min after the gradient. The peptide purity was determined to be 99%.

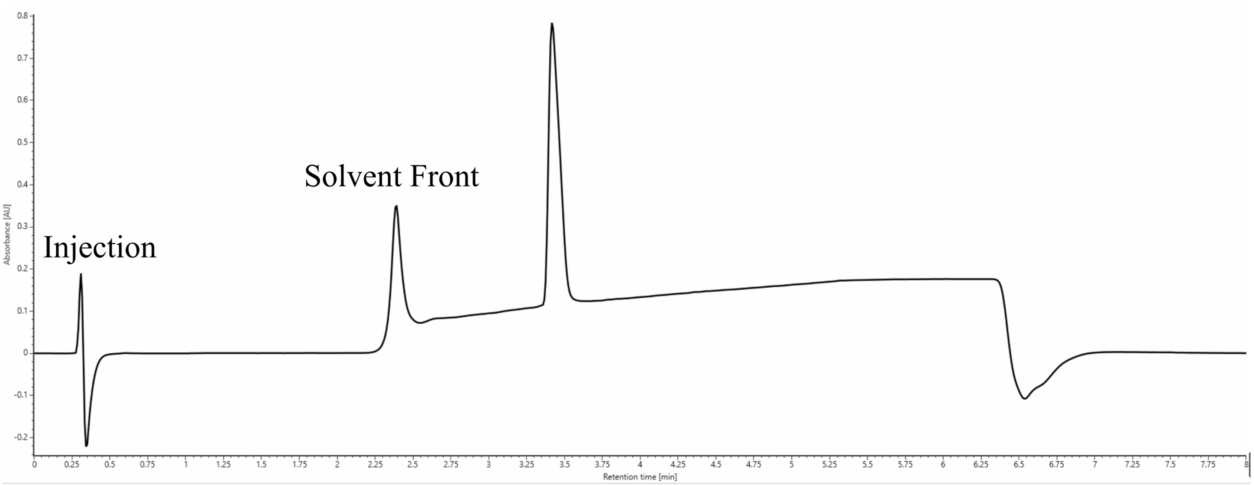

Synthesis of 8

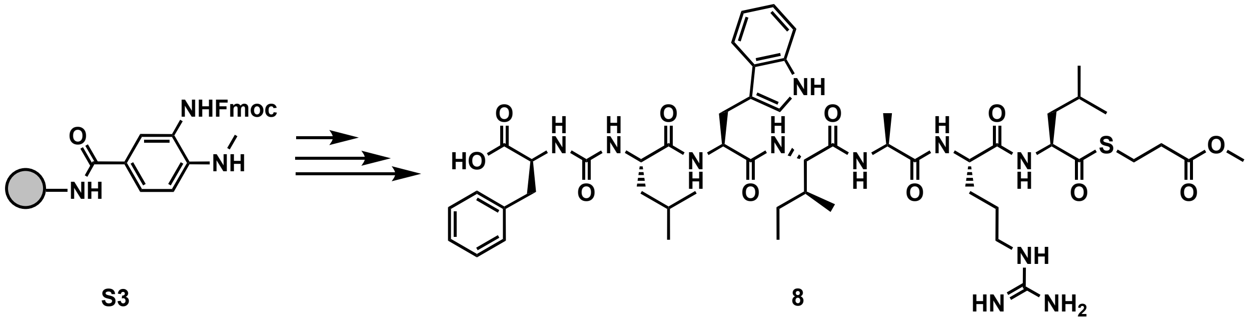

Peptide **8** was synthesized using the NDbz "safety-catch" resin. The synthesis was initiated from Fmoc-MeDbz-OH **S3** loaded resin (0.10 mmol), which was then coupled with the following amino acids:

Fmoc-L-Leu-OH, Fmoc-L-Arg(Pbf)-OH, Fmoc-L-Ala-OH, Fmoc-L-Ile-OH, Fmoc-L-Trp(Boc)-OH, and **5**. Methyl 3-mercaptopropionate was used as the cleaving thiol. The crude peptide was purified via reverse-phase semi-preparative HPLC using mobile phases of acetonitrile + 0.05% TFA (B) and H_2O + 0.05% TFA (A). Samples were eluted using a gradient mode with mobile phase B ranging from 0% to 70% over 22 min at a flow rate of 20 mL/min. The column was equilibrated with 0% mobile phase B for 5 min after and 1 min before the gradient to yield the TFA salt of **8** as an off-white solid (22 mg, 18% yield).

^{1}H NMR (800 MHz, DMSO-d_6): δ = 10.77 (s, 1H), 8.48 (d, J = 7.8, 1H), 8.09–7.84 (m, 3H), 7.81–7.58 (m, 2H), 7.52 (d, J = 7.9, 1H), 7.30 (d, J = 8.1, 1H), 7.25 (q, J = 8.4, 2H), 7.18 (p, J = 8.6, 4H), 7.09 (s, 1H), 7.04 (t, J = 7.5, 2H), 6.94 (t, J = 7.4, 2H), 6.37 (d, J = 8.0, 1H), 6.21 (d, J = 8.2, 1H), 4.58 (q, J = 7.2, 1H), 4.32 (m, 4H), 4.19–4.01 (m, 2H), 3.59 (d, J = 8.8, 3H), 3.37 (s, 4H), 3.17–3.03 (m, 3H), 3.01–2.76 (m, 6H), 1.83–0.93 (m, 17H), 0.93–0.61 (m, 20H).

^{13}C NMR (201 MHz, DMSO-d_6): δ = 201.8, 174.1, 173.4, 172.4, 172.1, 172.0, 171.5, 171.0, 157.5, 157.2, 137.6, 136.4, 129.7, 128.6, 127.8, 126.8, 123.8, 121.2, 118.7, 118.6, 111.6, 110.3, 57.9, 57.2, 54.3, 53.6, 52.3, 52.1, 52.0, 48.7, 42.4, 40.8, 37.9, 37.1, 33.8, 29.2, 27.4, 25.4, 24.6, 24.4, 23.6, 23.5, 23.2, 22.2, 21.4, 18.3, 15.6, 11.4.

Mass spec: HRMS (ESI-TOF) *m/z*: $[M + H]^+$ Calcd for $C_{52}H_{78}N_{11}O_{11}S$ 1064.5597; Found 1064.5587.

UPLC Trace Obtained using mobile phases of H_2O + 0.1% formic acid (A) and acetonitrile + 0.1% formic acid (B). Samples were eluted using a gradient mode with mobile phase B ranging from 0% to 70% over 4 min at a flow rate of 0.5 mL/min. The column was equilibrated with 0% mobile phase B for 1 min before and 2 min after the gradient. The peptide purity was determined to be 97%.

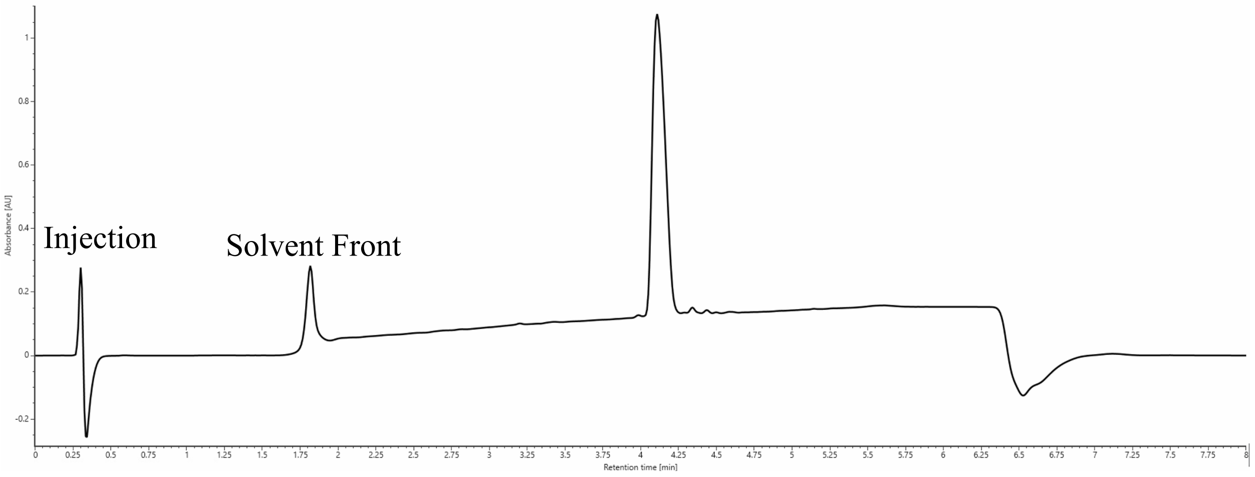

Synthesis of 9

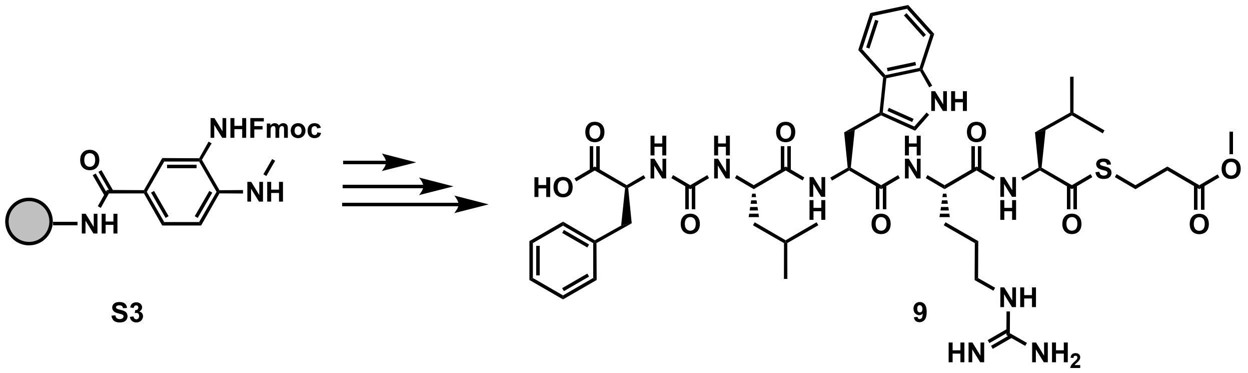

S3 9

Peptide **9** was synthesized using the NDbz "safety-catch" resin. The synthesis was initiated from Fmoc-MeDbz-OH **S3** loaded resin (0.10 mmol), which was coupled with the following amino acids: Fmoc-L-Leu-OH, Fmoc-L-Arg(Pbf)-OH, Fmoc-L-Trp(Boc)-OH, and **5**. Methyl 3-mercaptopropionate was used as the cleaving thiol. The crude peptide was purified via reverse-phase semi-preparative HPLC using mobile phases of acetonitrile + 0.05% TFA (B) and H_2O + 0.05% TFA (A). Samples were eluted using a gradient mode with mobile phase B ranging from 0% to 70% over 22 min at a flow rate of 20 mL/min. The column was equilibrated with 0% mobile phase B for 5 min after and 1 min before the gradient to yield the TFA salt of **9** as an off-white solid (16 mg, 16% yield).

^{1}H NMR (800 MHz, DMSO-d_6): δ = 10.80 (s, 1H), 8.46 (s, 1H), 8.11 (s, 1H), 7.91 (s, 1H), 7.64 (s, 1H), 7.53 (s, 1H), 7.31 (s, 1H), 7.26 (s, 2H), 7.18 (s, 3H), 7.11 (s, 1H), 7.04 (s, 1H), 6.94 (s, 2H), 6.35 (s, 1H), 6.24 (s, 1H), 4.55 (s, 1H), 4.35 (m, 3H), 4.04 (s, 1H), 3.59 (s, 3H), 3.17–3.05 (m, 3H), 2.98 (s, 4H), 2.89 (dd, *J* = 13.8, 7.2, 1H), 2.56 (s, 2H), 1.77 (s, 1H), 1.66 (s, 1H), 1.61–1.45 (m, 6H), 1.33 (s, 1H), 1.22 (s, 1H), 0.89 (s, 3H), 0.81 (m, 9H).

^{13}C NMR (201 MHz, DMSO-d_6): δ = 201.8, 174.0, 173.4, 172.1, 172.0, 171.7, 157.6, 157.2, 137.6, 136.4, 129.7, 128.6, 127.8, 126.8, 123.9, 121.2, 118.7, 118.5, 111.7, 110.2, 57.9, 54.3, 53.6, 52.3, 52.2, 51.9, 42.1, 40.2, 39.6, 37.9, 33.8, 29.2, 27.8, 25.3, 24.4, 24.4, 23.6, 23.5, 23.3, 22.1, 21.4.

Mass spec: HRMS (ESI-TOF) *m/z*: $[M + H]^+$ Calcd for $C_{43}H_{62}N_9O_9S$ 880.4386; Found 880.4364.

UPLC Trace Obtained using mobile phases of H_2O + 0.1% formic acid (A) and acetonitrile + 0.1% formic acid (B). Samples were eluted using a gradient mode with mobile phase B ranging from 0% to 70% over 4 min at a flow rate of 0.5 mL/min. The column was equilibrated with 0% mobile phase B for 1 min before and 2 min after the gradient. The peptide purity was determined to be 99%.

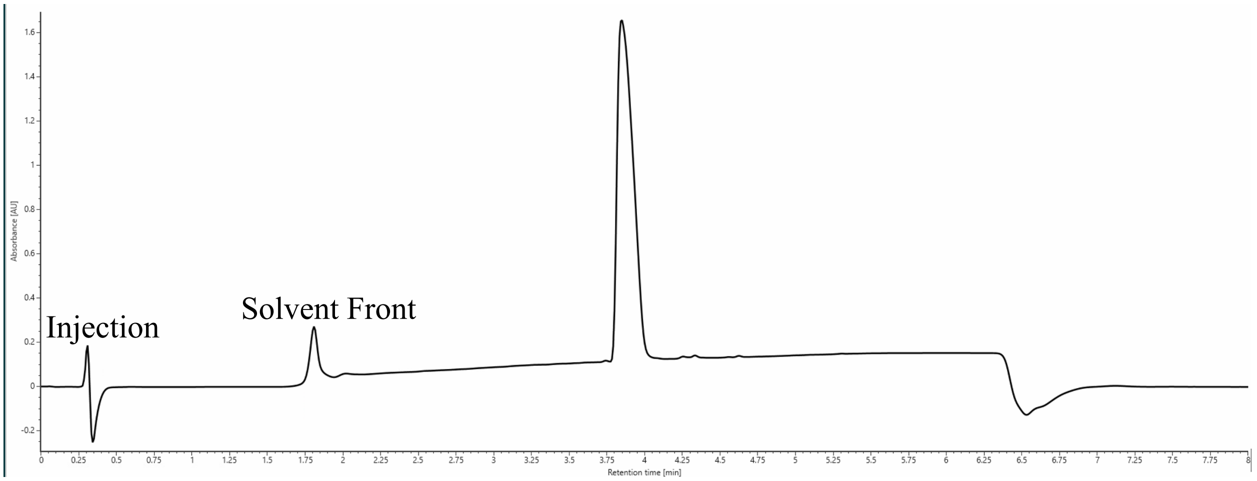

Synthesis of 10

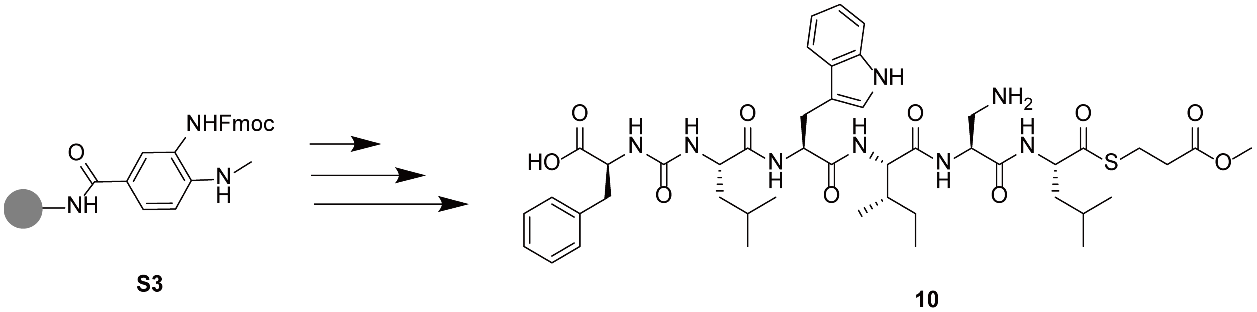

S3 **10**

Peptide **10** was synthesized using the NDbz "safety-catch" resin. The synthesis was initiated from Fmoc-MeDbz-OH **S3** loaded resin (0.10 mmol), which was then coupled with the following amino acids: Fmoc-L-Leu-OH, Fmoc-L-Dap(Boc)-OH, Fmoc-L-Ile-OH, Fmoc-L-Trp(Boc)-OH, and **5**. Methyl 3-mercaptopropionate was used as the cleaving thiol. The crude peptide was purified via reverse-phase semi-preparative HPLC using mobile phases of acetonitrile + 0.05% TFA (B) and H_2O + 0.05% TFA (A). Samples were eluted using a gradient mode with mobile phase B ranging from 0% to 70% over 22 min at a flow rate of 20 mL/min. The column was equilibrated with 0% mobile phase B for 5 min after and 1 min before the gradient to yield the TFA salt of **10** as an off-white solid (5 mg, 5% yield).

^{1}H NMR (800 MHz, DMSO-d_6): δ = 10.91–10.72 (m, 1H), 8.69 (d, J = 7.8, 1H), 8.35 (s, 1H), 8.16 (d, J = 7.5, 1H), 7.69 (d, J = 7.8, 1H), 7.54 (d, J = 7.9, 1H), 7.37–6.88 (m, 9H), 6.43 (d, J = 7.4, 1H), 6.28 (d, J = 8.1, 1H), 4.70–4.51 (m, 2H), 4.44–4.19 (m, 3H), 4.08 (q, J = 7.5, 1H), 3.61 (s, 4H), 3.21 (m, 3H), 3.06–2.83 (m, 6H), 2.58 (td, J = 7.0, 2.2, 3H), 1.79 (dtt, J = 13.2, 10.0, 5.2, 1H), 1.69–1.46 (m, 4H), 1.44–1.21 (m, 3H), 1.10–1.01 (m, 1H), 0.95–0.70 (m, 19H).

^{13}C NMR (201 MHz, DMSO-d_6): δ = 201.3, 174.3, 173.9, 172.1, 172.0, 171.7 169.5, 157.8, 137.8, 136.5, 129.8, 128.6, 127.8, 126.9, 123.9, 121.3, 118.7, 118.6, 111.8, 110.6, 58.2, 57.6, 54.5, 53.8, 52.4, 52.0, 51.0, 42.4, 38.0, 37.2, 33.8, 27.2, 24.5, 24.4, 23.8, 23.5, 23.3, 22.3, 21.4, 15.6, 11.7.

Mass spec: HRMS (ESI-TOF) *m/z*: $[M + H]^+$ Calcd for $C_{46}H_{67}N_8O_{10}S$ 923.4695; Found 923.3919.

UPLC Trace Obtained using mobile phases of H_2O + 0.1% formic acid (A) and acetonitrile + 0.1% formic acid (B). Samples were eluted using a gradient mode with mobile phase B ranging from 0% to 70% over 4 min at a flow rate of 0.5 mL/min. The column was equilibrated with 0% mobile phase B for 1 min before and 2 min after the gradient. The peptide purity was determined to be 98%.

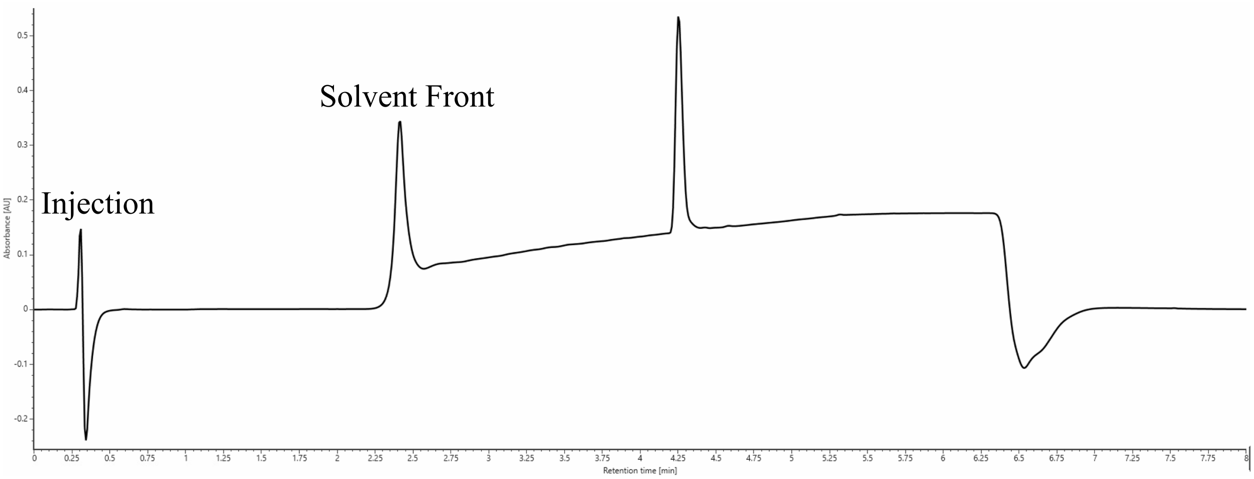

Synthesis of 11

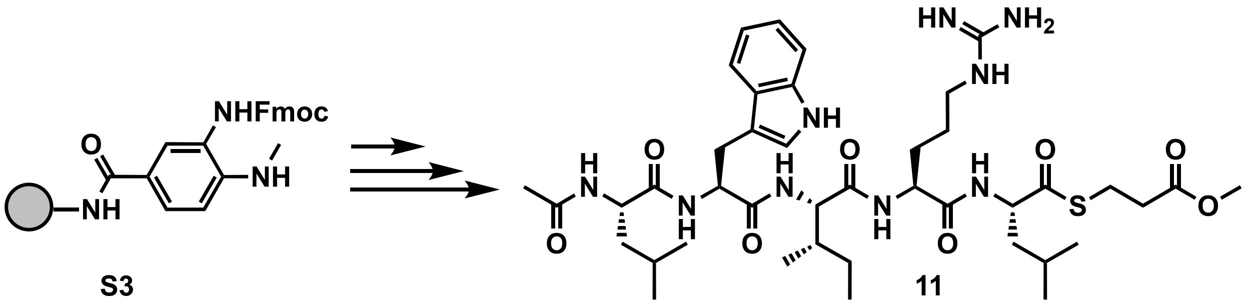

Peptide **11** was synthesized using the NDbz "safety-catch" resin. The synthesis was initiated from Fmoc-MeDbz-OH **11** loaded resin (0.10 mmol), which was coupled with the following amino acids: Fmoc-L-Leu-OH, Fmoc-L-Arg(Pbf)-OH, Fmoc-L-Ile-OH, Fmoc-L-Trp(Boc)-OH, and Fmoc-L-Leu-OH. The N-terminal Fmoc protecting group was removed with 20% piperidine-DMF, and acetylated with 50 equivalents (5 mmol) acetic anhydride and 50 equivalents of pyridine (5 mmol) in DMF for 1 h. Methyl 3-

mercaptopropionate was used as the cleaving thiol. The crude peptide was purified via reverse-phase semi-preparative HPLC using mobile phases of acetonitrile + 0.05% TFA (B) and H_2O + 0.05% TFA (A). Samples were eluted using a gradient mode with mobile phase B ranging from 0% to 70% over 22 min at a flow rate of 20 mL/min. The column was equilibrated with 0% mobile phase B for 5 min after and 1 min before the gradient to yield the TFA salt of **11** as an off-white solid (45 mg, 47% yield).

^{1}H NMR (800 MHz, DMSO-d_6): δ = 10.85–10.78 (m, 1H), 8.52 (d, J = 7.8, 1H), 8.17–7.95 (m, 3H), 7.66 (d, J = 8.6, 2H), 7.53 (d, J = 7.9, 1H), 7.31 (d, J = 8.1, 2H), 7.13–6.89 (m, 4H), 4.54 (td, J = 8.5, 4.5, 1H), 4.47–4.35 (m, 2H), 4.34–4.19 (m, 2H), 3.60 (d, J = 2.1, 3H), 3.35 (t, J = 3.6, 5H), 3.12 (tt, J = 13.3, 6.0, 3H), 3.06–2.91 (m, 3H), 1.80 (s, 4H), 1.71–1.60 (m, 2H), 1.53 (m, 6H), 1.44–1.30 (m, 3H), 1.04 (ddd, J = 13.2, 9.2, 6.9, 1H), 1.00–0.68 (m, 19H).

^{13}C NMR (201 MHz, DMSO-d_6): δ = 201.8, 172.7, 172.1, 172.0, 171.5, 171.0, 169.7, 157.2, 136.4, 127.7, 123.8, 121.2, 118.7, 118.6, 111.6, 110.4, 57.8, 57.1, 53.5, 52.4, 52.0, 51.4, 41.0, 40.8, 37.4, 33.8, 29.2, 27.3, 25.5, 24.5, 24.5, 24.4, 23.7, 23.3, 22.8, 22.0, 21.2, 15.5, 11.5.

Mass spec: HRMS (ESI-TOF) *m/z*: $[M + H]^+$ Calcd for $C_{41}H_{66}N_9O_8S$ 844.4750; Found 844.4798.

UPLC Trace Obtained using mobile phases of H_2O + 0.1% formic acid (A) and acetonitrile + 0.1% formic acid (B). Samples were eluted using a gradient mode with mobile phase B ranging from 0% to 70% over 11 min at a flow rate of 0.5 mL/min. The column was equilibrated with 0% mobile phase B for 1 min before and 2 min after the gradient. The peptide purity was determined to be 98%.

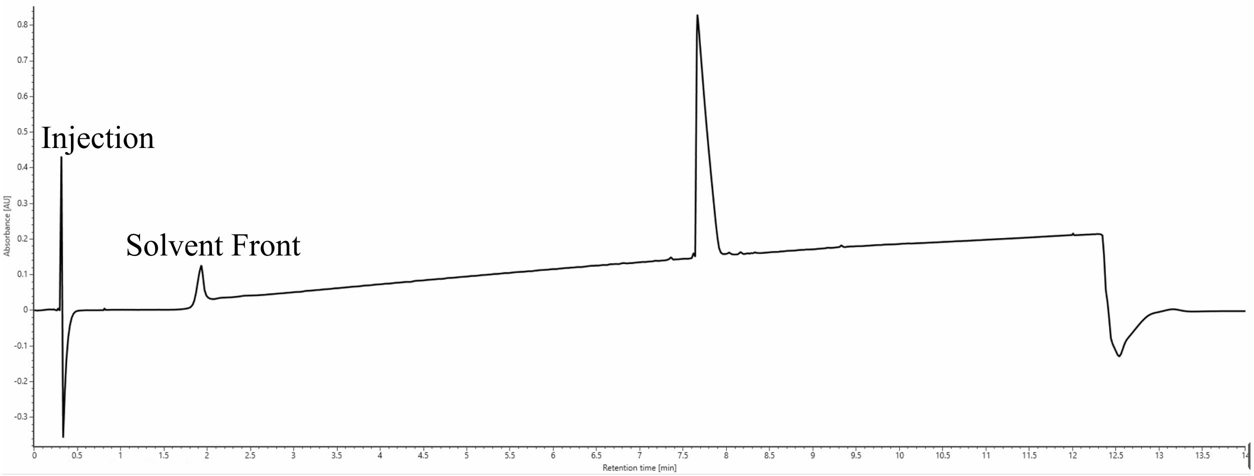

Synthesis of 12

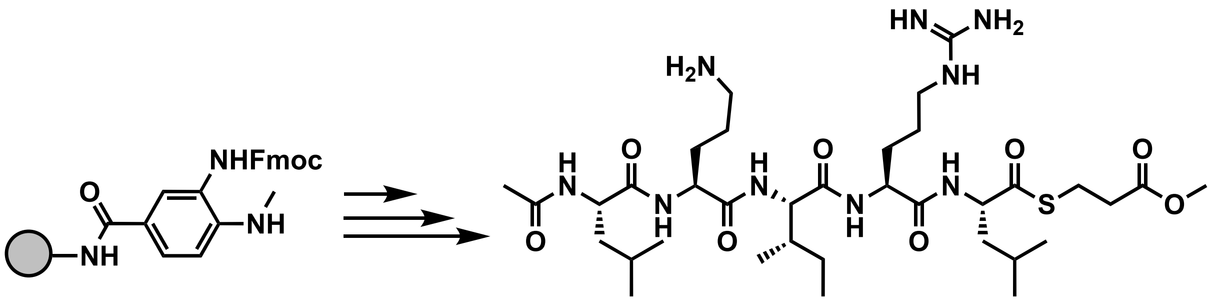

S3 **12**

Peptide **12** was synthesized using the NDbz "safety-catch" resin. The synthesis was initiated from Fmoc-MeDbz-OH **12** loaded resin (0.20 mmol), which was coupled with the following amino acids: Fmoc-L-Leu-OH, Fmoc-L-Arg(Pbf)-OH, Fmoc-L-Ile-OH, Fmoc-L-Orn(Boc)-OH, and Fmoc-L-Leu-OH. The N-terminal Fmoc protecting group was removed with 20% piperidine-DMF, and acetylated with 50 equivalents (5 mmol) acetic anhydride and 50 equivalents of pyridine (5 mmol) in DMF for 1 h. Methyl 3-mercaptopropionate was used as the cleaving thiol. The crude peptide was purified via reverse-phase semi-preparative HPLC using mobile phases of acetonitrile + 0.05% TFA (B) and H_2O + 0.05% TFA (A). Samples were eluted using a gradient mode with mobile phase B ranging from 0% to 70% over 22 min at a flow rate of 20 mL/min. The column was equilibrated with 0% mobile phase B for 5 min after and 1 min before the gradient to yield the TFA salt of **12** as an off-white solid (32 mg, 16% yield).

^{1}H NMR (800 MHz, DMSO-d_6): δ = 8.56 (d, J = 7.8, 1H), 8.18 (d, J = 8.2, 1H), 8.06 (m, 2H), 7.77 (q, J = 5.8, 4H), 7.58 (d, J = 8.7, 1H), 4.44–4.20 (m, 5H), 3.60 (s, 3H), 3.17–3.07 (m, 2H), 2.98 (td, J = 7.0, 2.8, 2H), 2.78 (h, J = 5.9, 2H), 2.56 (t, J = 7.1, 5H), 1.81–1.36 (m, 18H), 0.93–0.74 (m, 20H).

^{13}C NMR (201 MHz, DMSO-d_6): δ = 201.8, 173.1, 172.2, 172.1, 171.4, 171.1, 169.8, 157.3, 57.9, 57.0, 52.4, 52.3, 52.0, 51.6, 41.3, 40.9, 38.9, 37.6, 33.9, 29.3, 28.7, 25.6, 24.7, 24.5, 24.2, 23.7, 23.5, 23.4, 22.9, 22.0, 21.3, 15.6, 11.6.

Mass spec: HRMS (ESI-TOF) *m/z*: $[M + H]^+$ Calcd for $C_{35}H_{66}N_9O_8S$ 772.4750; Found 772.4739.

HPLC Trace Obtained using mobile phases of H_2O + 0.1% formic acid (A) and acetonitrile + 0.1% formic acid (B). Samples were eluted using a gradient mode with mobile phase B ranging from 0% to 60% over 21 min at a flow rate of 1 mL/min. The column was equilibrated with 0% mobile phase B for 1 min before and 5 min after the gradient. The peptide purity was determined to be 95%.

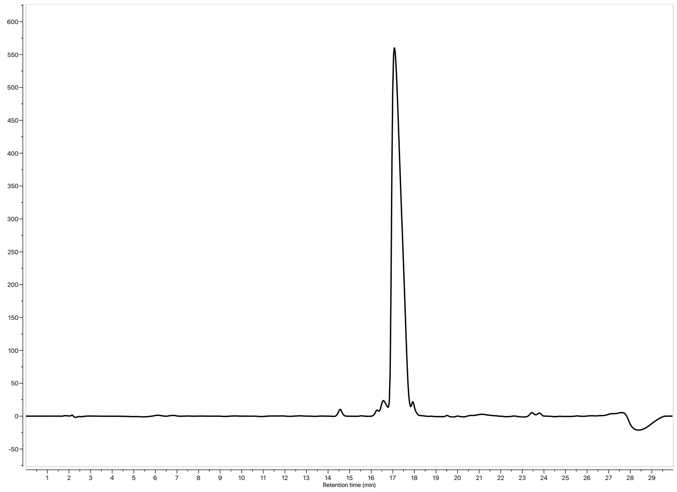

600
550
500
450
400
350
300
250
200
150
100
50
0
-50
1 2 3 4 5 6 7 8 9 10 11 12 13 14 15 16 17 18 19 20 21 22 23 24 25 26 27 28 29
Retention time (min)

SUPPLEMENTARY TABLES

Table S1: Enzymatic conversion yields for cyclic and hydrolysis products by the two BulbE-TE enzymes, C961S BulbE-TE, and no enzyme control

**BulbE-TE from *Microbulbifer* sp. MLAF003**		
Substrates	Cyclic product yields	Hydrolysis product yields
**1**	29%	0.5%
**2**	4%	7%
**6**	product not detected	18%
**7**	product not detected	45%
**8**	product not detected	1%
**9**	product not detected	5%
**10**	3%	47%
**11**	31%	7%
**12** (pH 6.0)	13%	4%
**12** (pH 7.5)	72%	26%
**12** (pH 9.0)	35%	63%
C961S mutant enzyme		
**12** (pH 6.0)	0.5%	10%
**12** (pH 7.5)	55%	43%
**12** (pH 9.0)	10%	89%
Without enzyme		
**12** (pH 6.0)	product not detected	0.1%
**12** (pH 7.5)	69%	29%
**12** (pH 9.0)	9%	89%
**BulbE-TE from *Microbulbifer* sp. VAAF005**		
**2**	15%	77%

SUPPLEMENTARY FIGURES

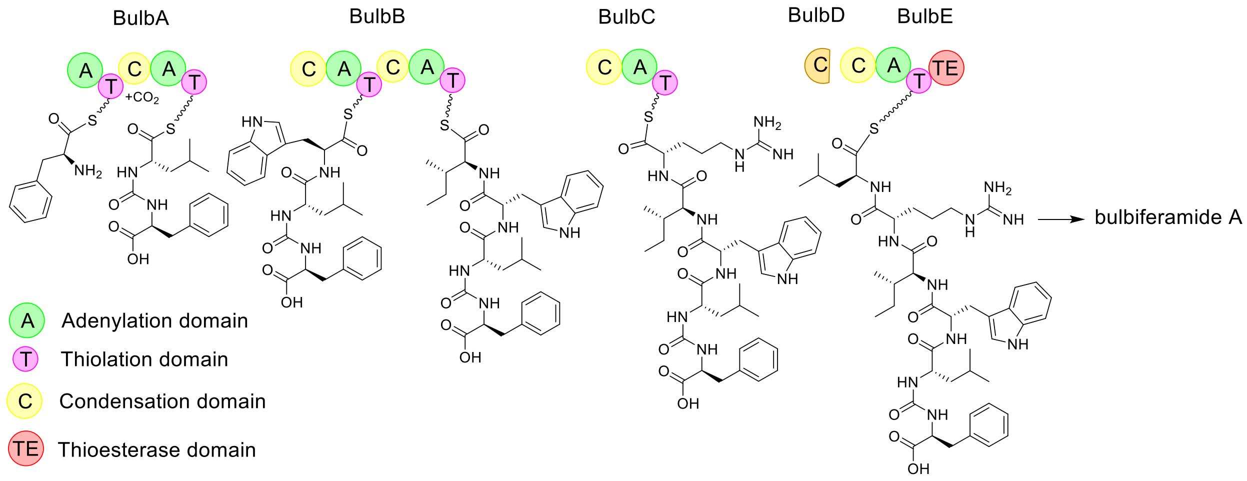

Figure S1: Proposed biosynthetic assembly line of bulbiferamide A. Note the presence of the TE domain at the end of the BulbE NRPS, which is referred to as the BulbE-TE in this report.

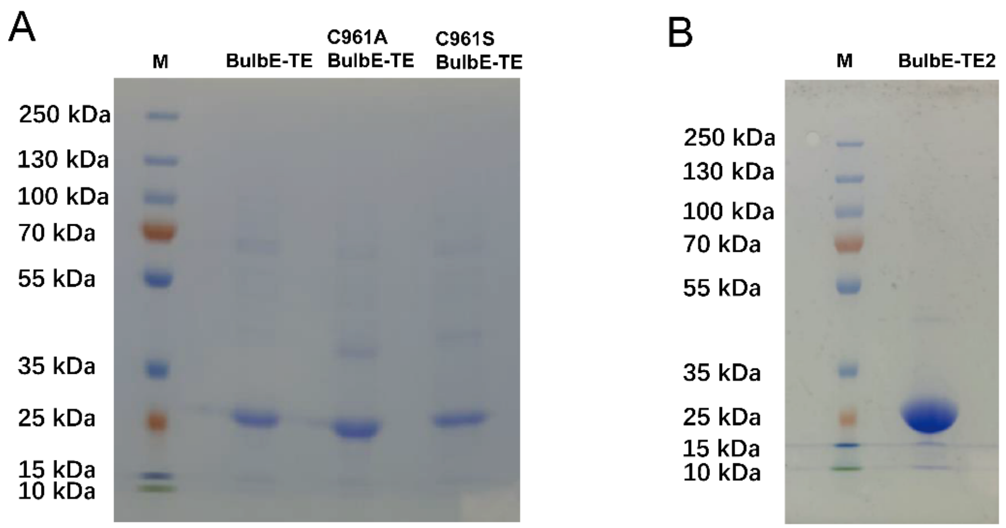

Figure S2: (**A**) SDS-PAGE of purified recombinant wild type and mutant forms of BulbE-TE from *Microbulbifer* sp. MLAF003. Expected molecular weight of protein is approximately 30.5 kDa. (**B**) SDS-PAGE of purified recombinant wild type BulbE-TE from *Microbulbifer* sp. VAAF005 (referred to as BulbE-TE2 in the figure). Expected molecular weight of protein is approximately 30.0 kDa.

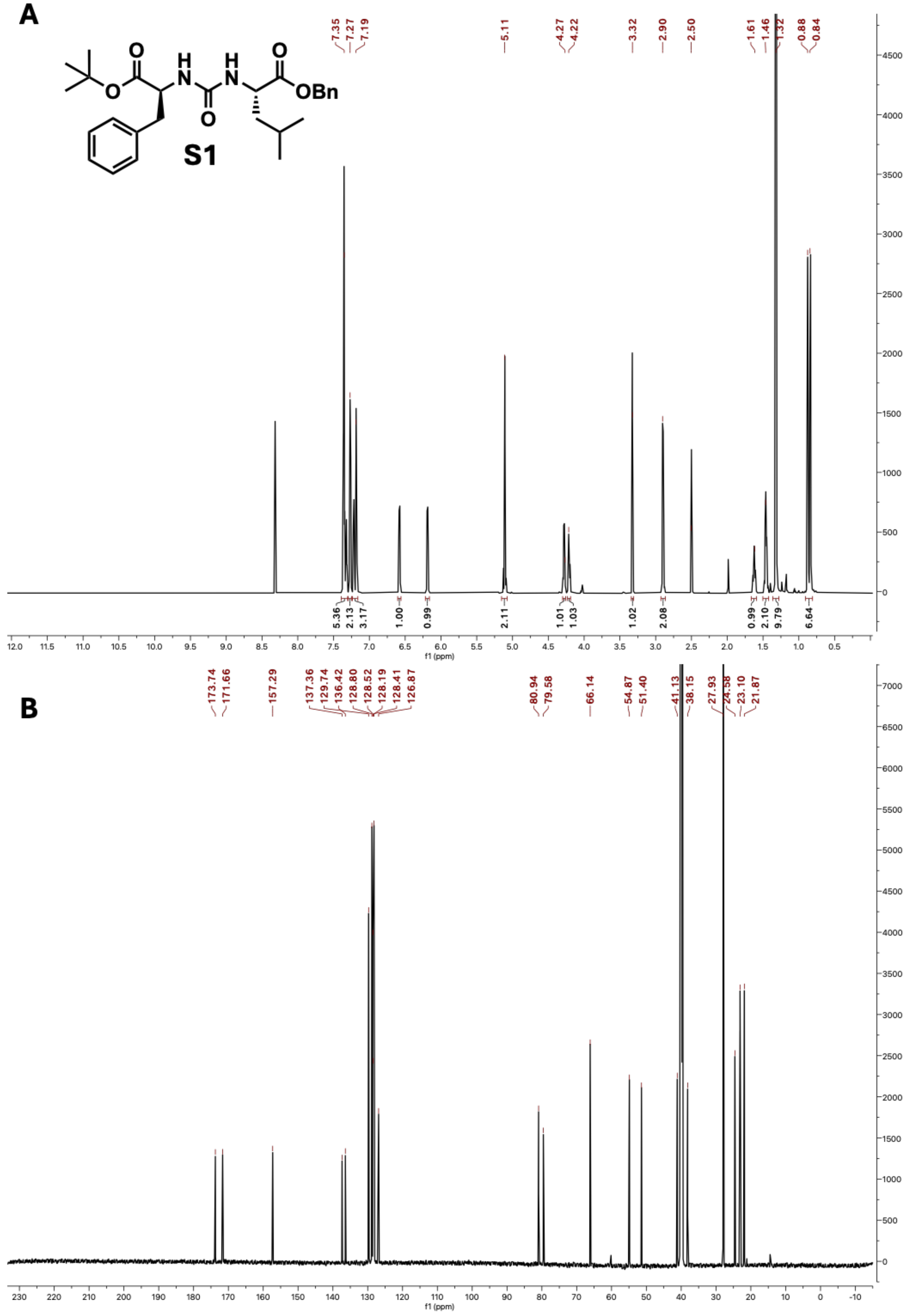

Figure S3: (**A**) ^{1}H (800 MHz) and (**B**) ^{13}C (201 MHz) NMR spectra of **S1** in DMSO-d_6.

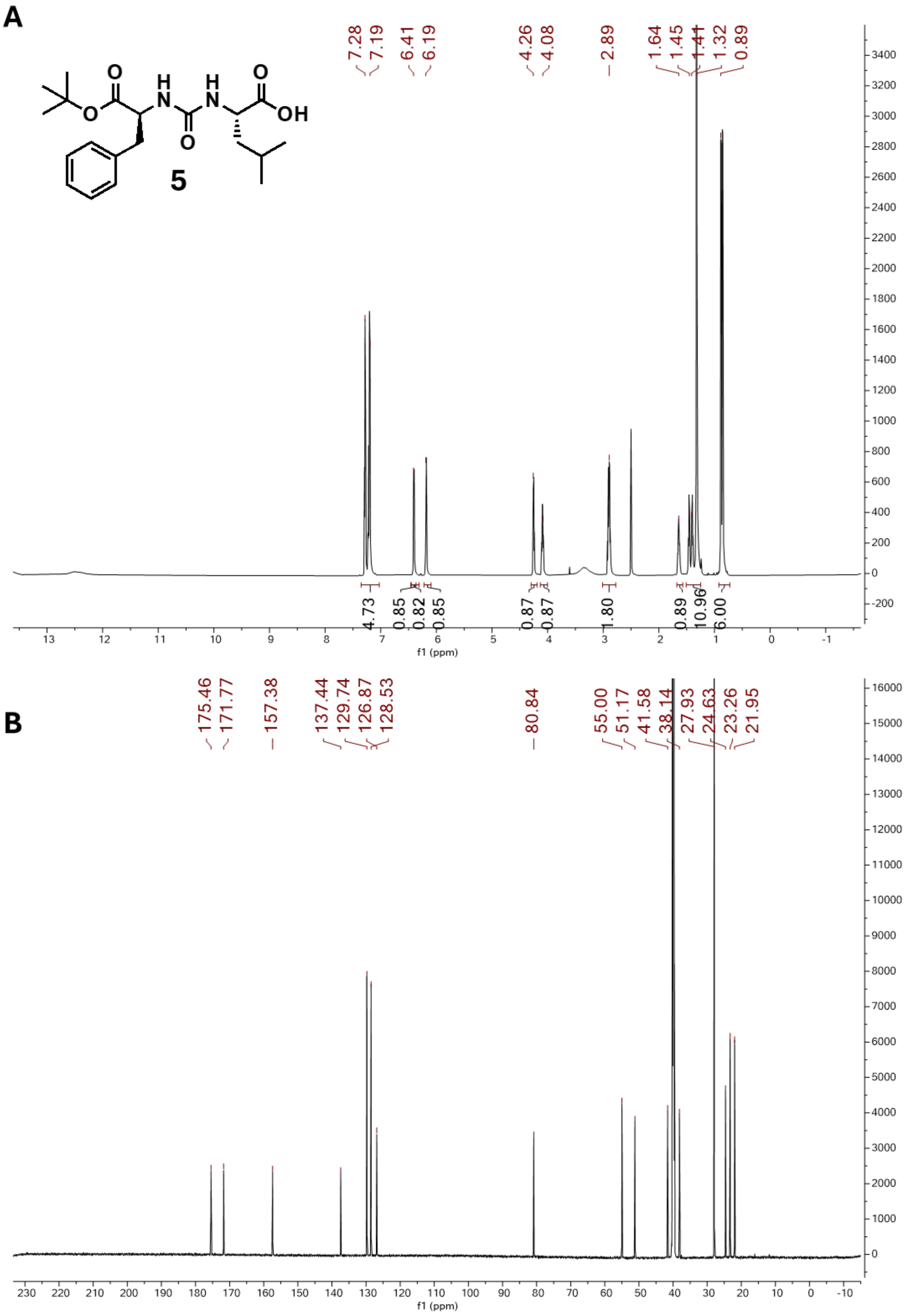

Figure S4: (**A**) ^{1}H (800 MHz) and (**B**) ^{13}C (201 MHz) NMR spectra of **5** in DMSO-d_6.

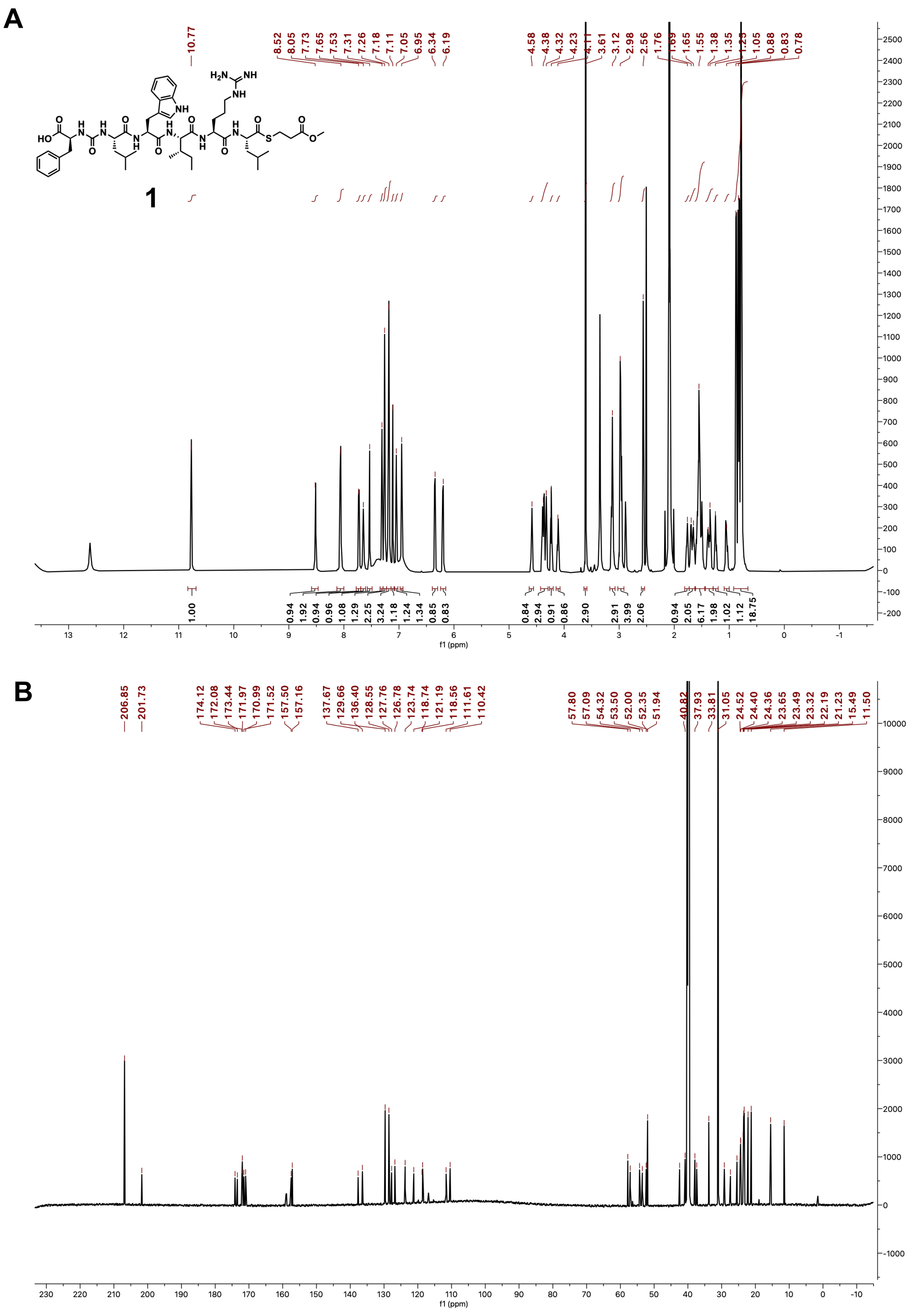

Figure S5: (**A**) ^{1}H (800 MHz) and (**B**) ^{13}C (201 MHz) NMR spectra of **1** in DMSO-d_6.

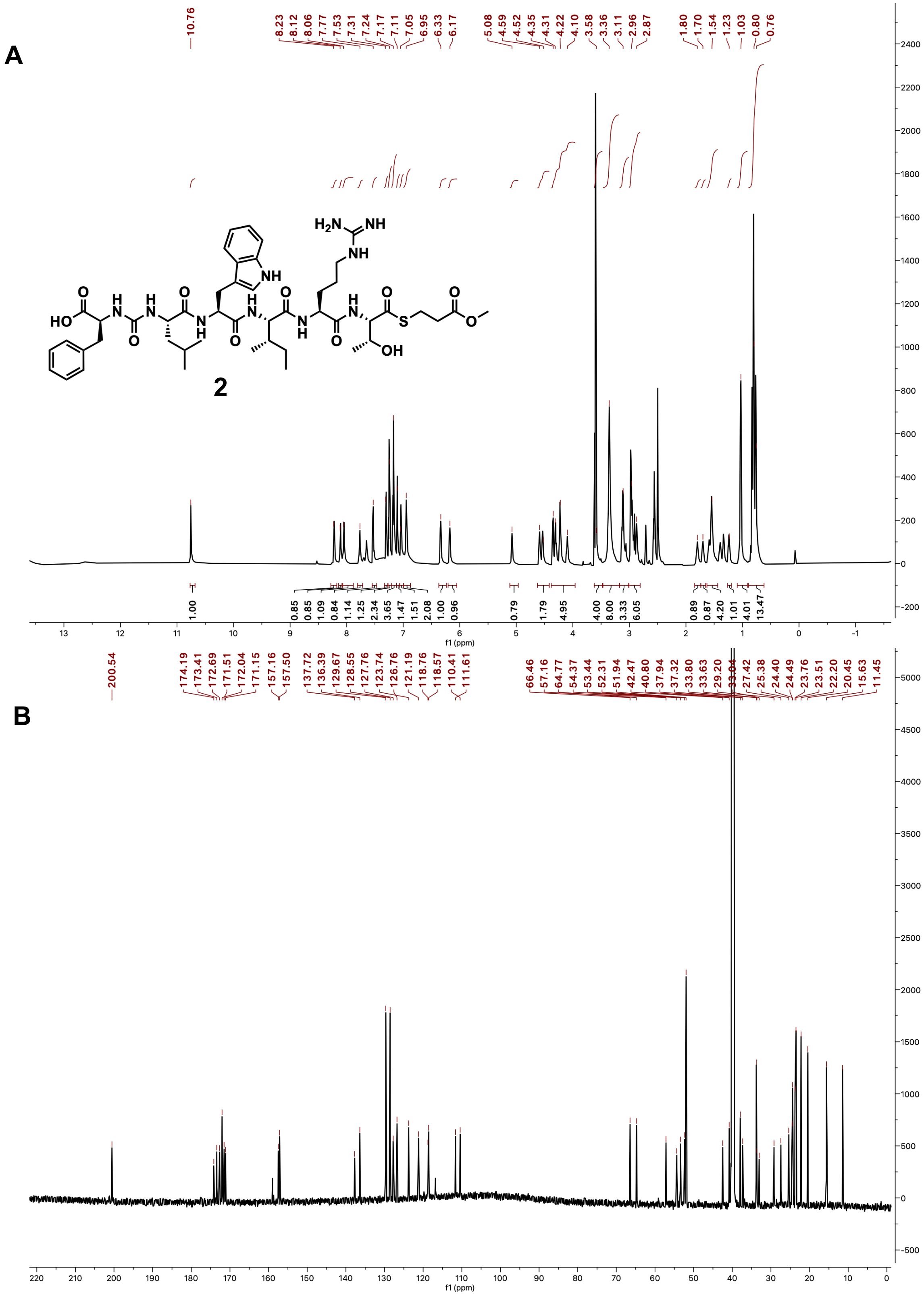

Figure S6: (**A**) ^{1}H (800 MHz) and (**B**) ^{13}C (201 MHz) NMR spectra of **2** in DMSO-d_6.

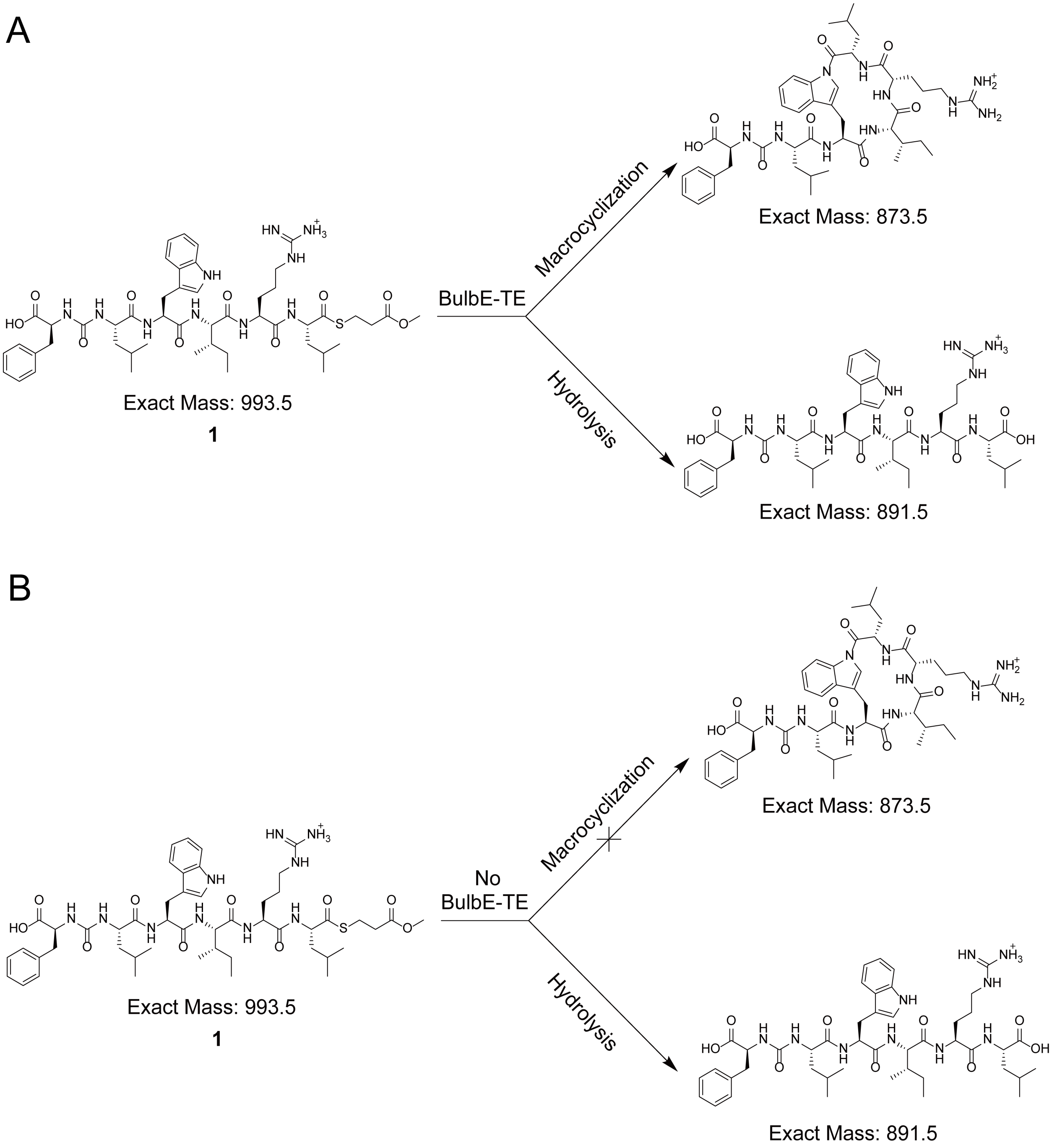

Figure S7: (**A**) Scheme for enzymatic reaction of substrate **1** with BulbE-TE to yield cyclic product bulbiferamide A and a thioester hydrolysis product. (**B**) Negative control of enzymatic reaction of substrate **1** without BulbE-TE did not yield cyclic product bulbiferamide A, only hydrolysis product.

A

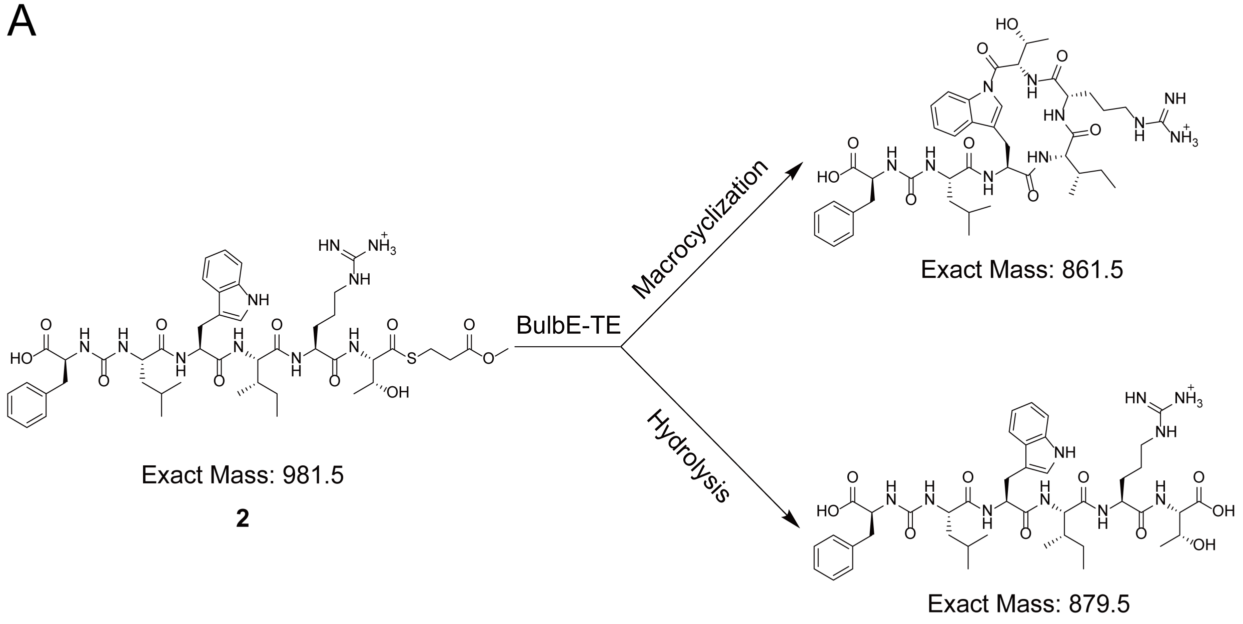

B

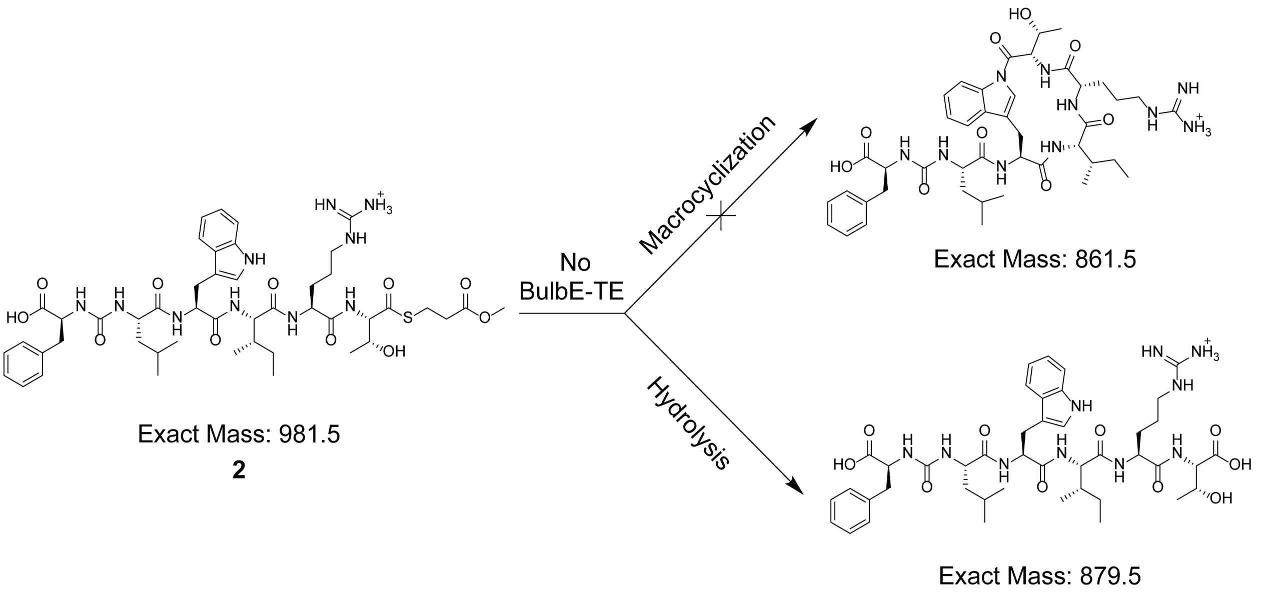

Figure S8: (**A**) Scheme for enzymatic reaction of substrate **2** with BulbE-TE to yield cyclic product bulbiferamide B and a thioester hydrolysis product. (**B**) Negative control of enzymatic reaction of substrate **2** without BulbE-TE did not yield cyclic product bulbiferamide B, only hydrolysis product.

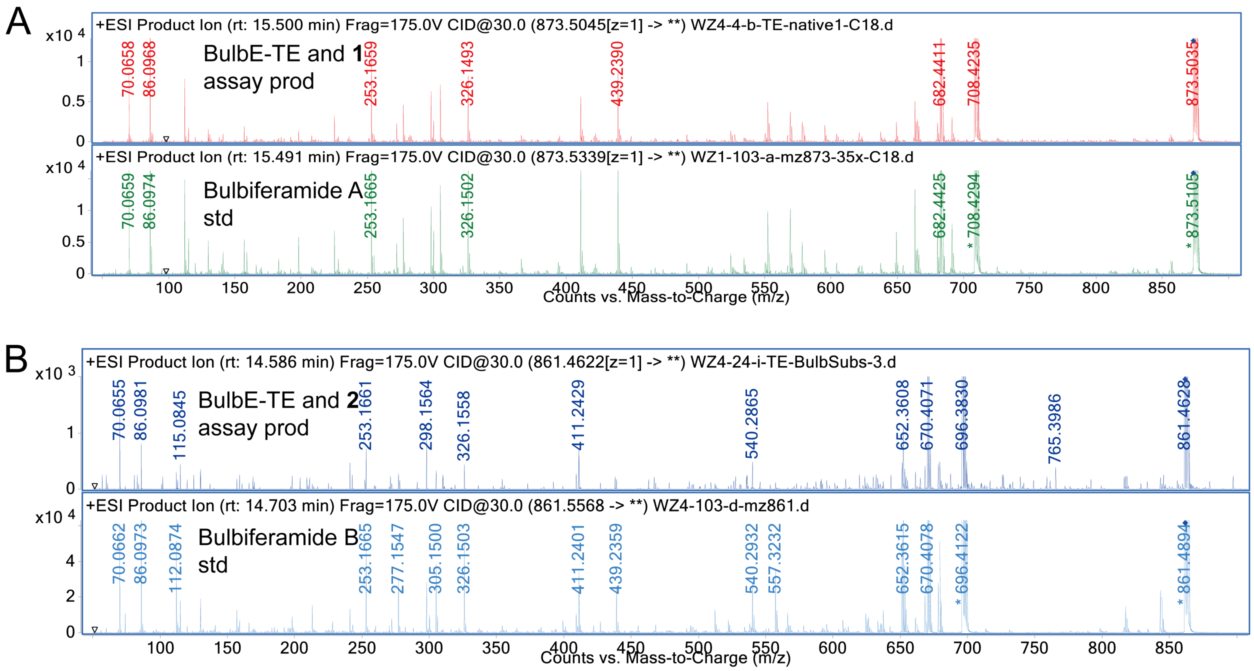

Figure S9: (**A**) MS^2 spectra for enzymatic product generated by BulbE-TE from **1** (top), and bulbiferamide A standard (bottom). (**B**) MS^2 spectra for enzymatic product generated by BulbE-TE starting from **2** (top), and bulbiferamide B standard (bottom). $[M+H]^{1+}$ precursor ions were chosen for fragmentation in each case with identical fragmentation energies.

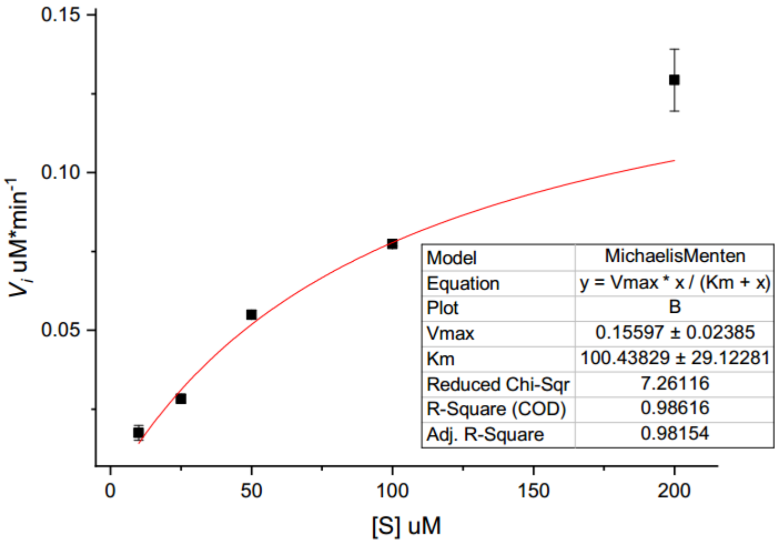

Figure S10. Michaels-Menten kinetics curve for macrocylization of **1** by BulbE-TE.

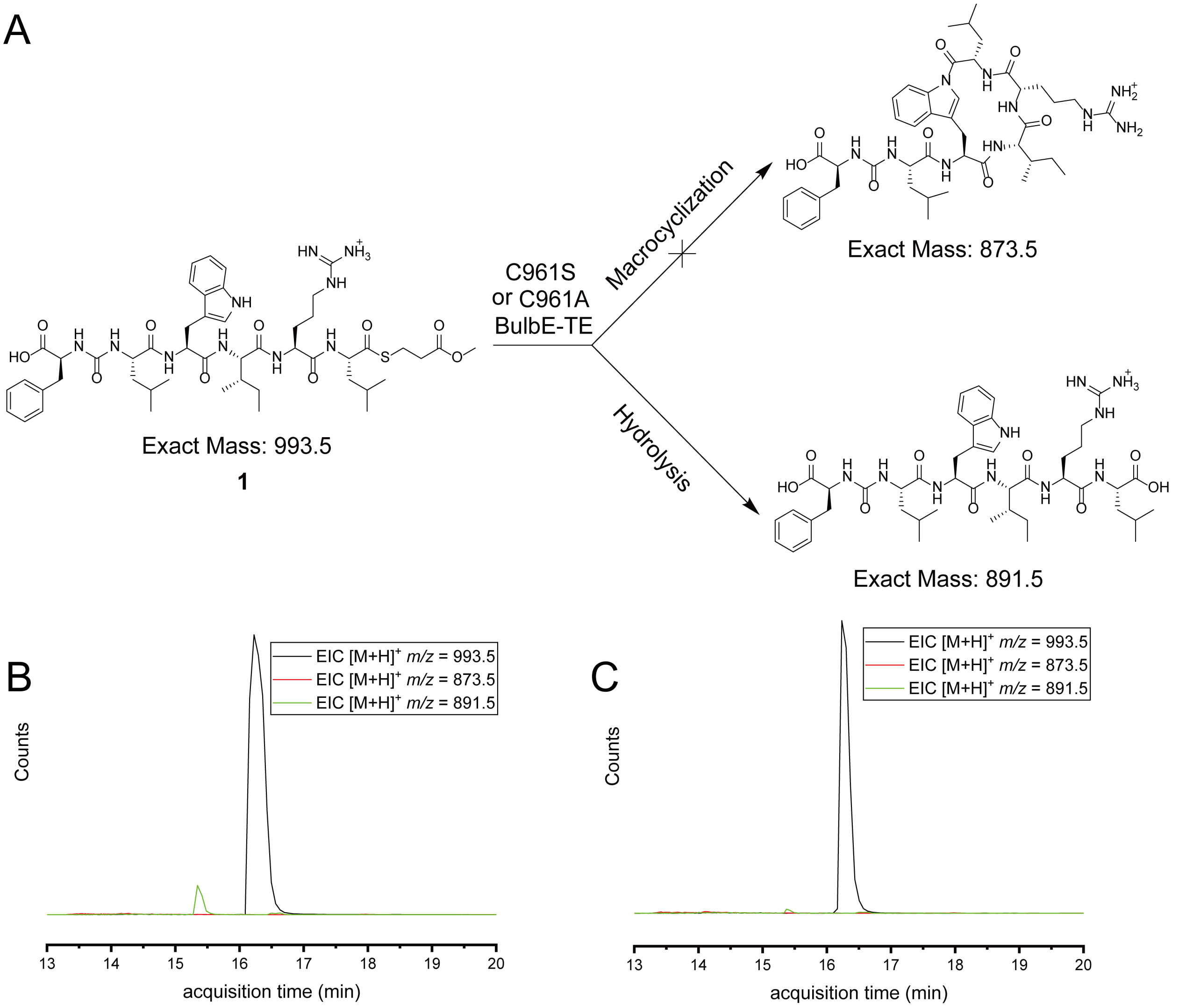

Figure S11: (**A**) Enzymatic reaction of substrate **1** with C961S or C961A mutated BulbE-TE did not yield cyclic product bulbiferamide A, only hydrolysis product. (**B**) LCMS analysis of C961S mutated BulbE-TE reaction with **1**. EICs showing the presence of the substrate **1** (black) and the thioester hydrolysis product (green). No cyclic product was observed (red). (**C**) LCMS analysis of the C961A mutated BulbE-TE reaction with **1**. EICs showing the presence of the substrate **1** (black) and the thioester hydrolysis product (green). No cyclic product was observed (red).

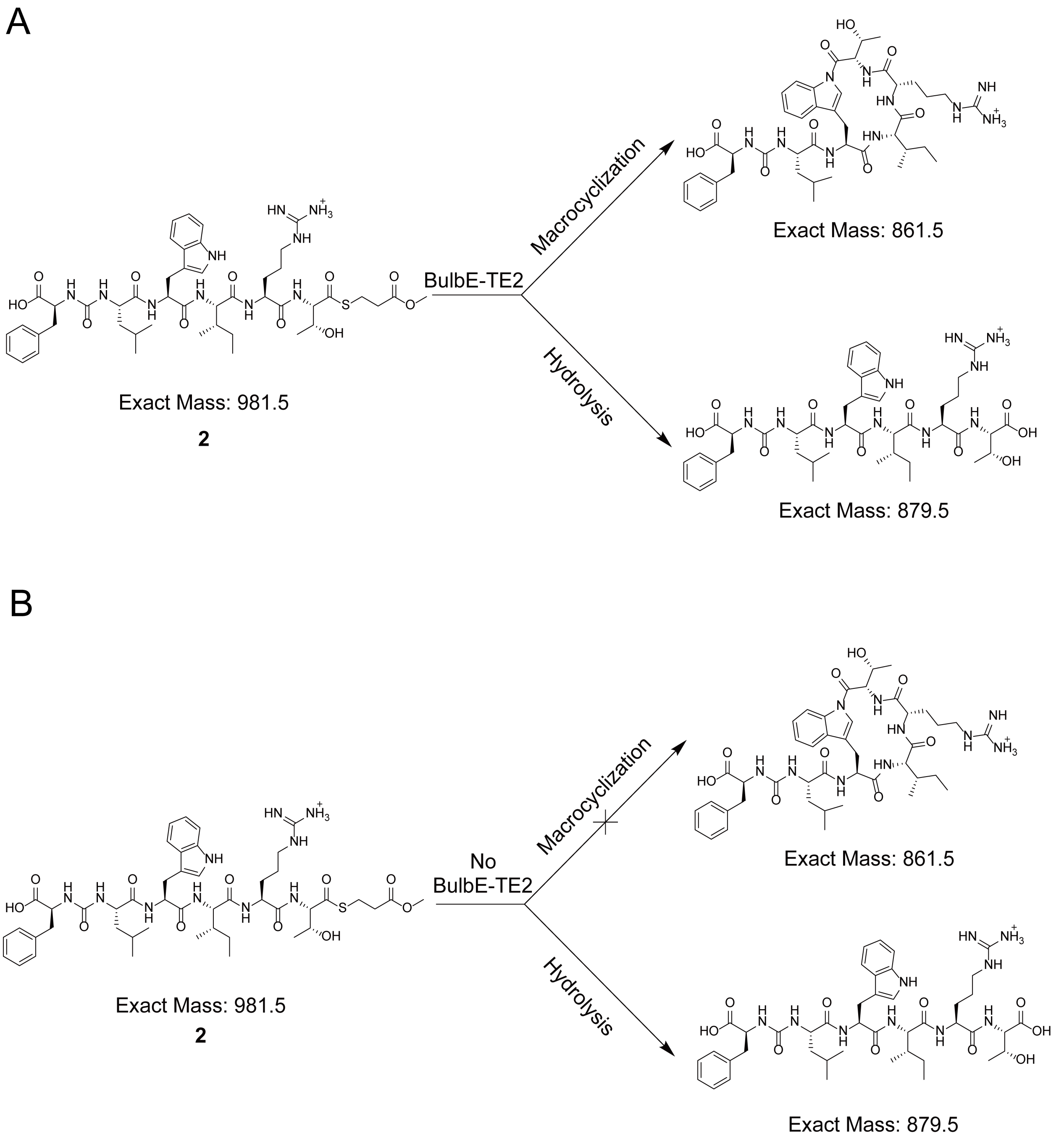

Figure S12: (**A**) Scheme for enzymatic reaction of substrate **2** with *Microbulbifer* sp. VAAF005-derived BulbE-TE (referred to as BulbE-TE2 in the figure) to yield cyclic product bulbiferamide B and a thioester hydrolysis product. (**B**) Negative control of enzymatic reaction of substrate **2** while omitting the enzyme did not yield cyclic product bulbiferamide B, only hydrolysis product was observed in this reaction.

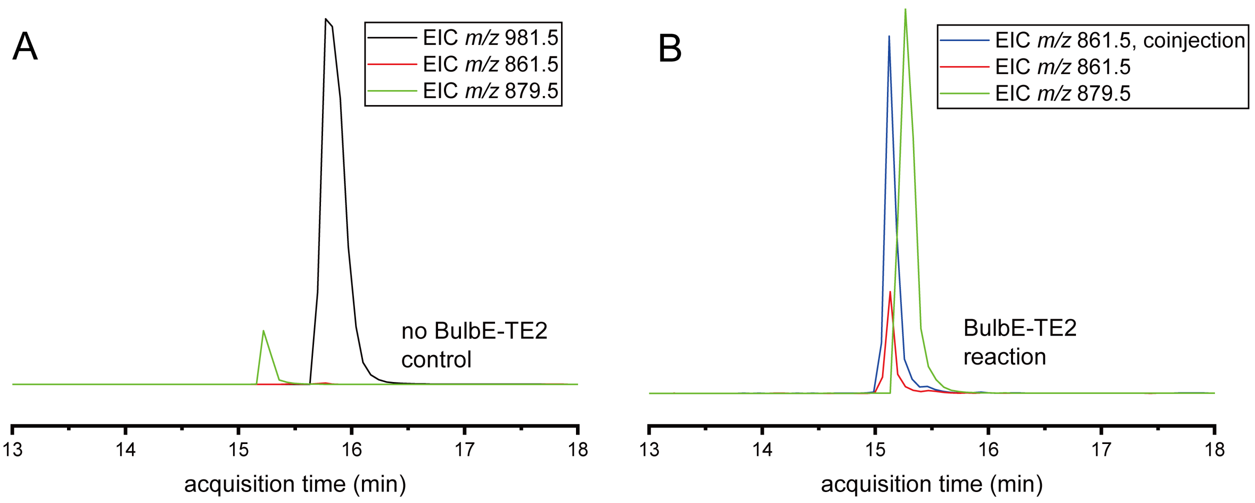

Figure S13: (**A**) EICs demonstrating the presence of **2**, hydrolyzed, and macrocyclized products in the reaction where the BulbE-TE from *Microbulbifer* sp. VAAF005 (also referred to as BulbE-TE2) was omitted. (**B**) EICs for hydrolyzed and macrocyclized products in the reaction in the presence of BulbE-TE2. The "coinjection" EIC refers to a spiking experiment in which bulbiferamide B was added to the quenched enzymatic reaction to confirm co-elution with the macrocyclized enzymatic product.

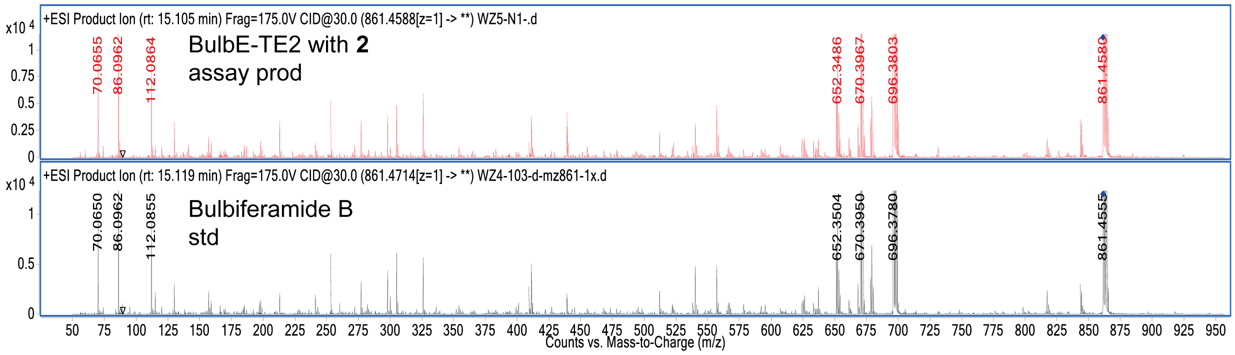

Figure S14: MS^2 spectra for enzymatic product generated by BulbE-TE2 starting from **2** (top), and bulbiferamide B standard (bottom). $[M+H]^{1+}$ precursor ions were chosen for fragmentation in each case with identical fragmentation energies.

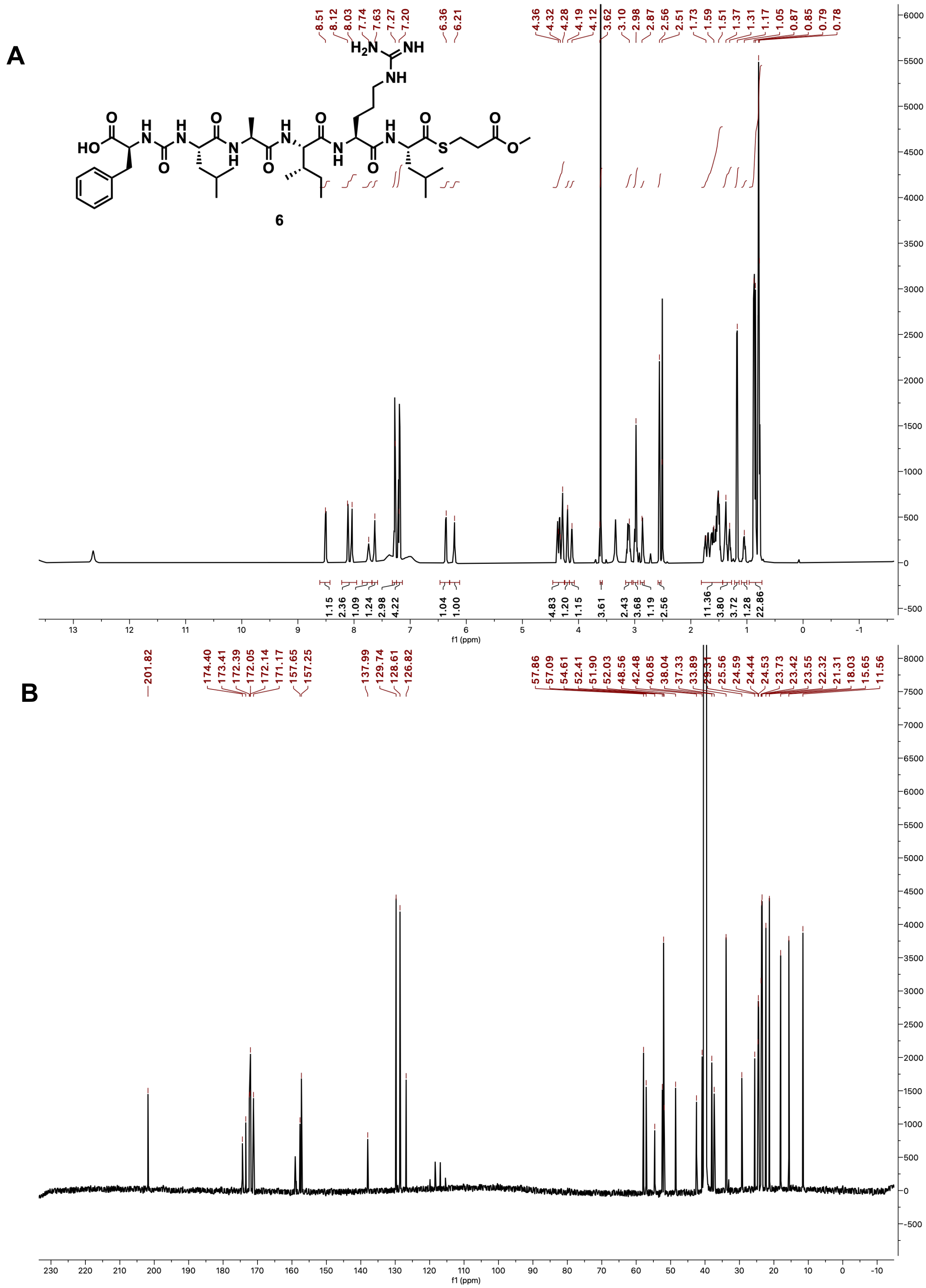

Figure S15: (**A**) ^{1}H (800 MHz) and (**B**) ^{13}C (201 MHz) NMR spectra of **6** in DMSO-d_6.

A

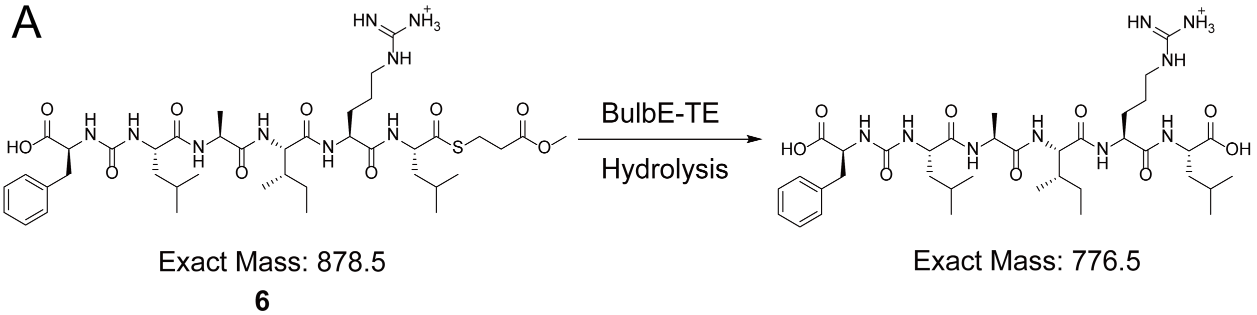

B

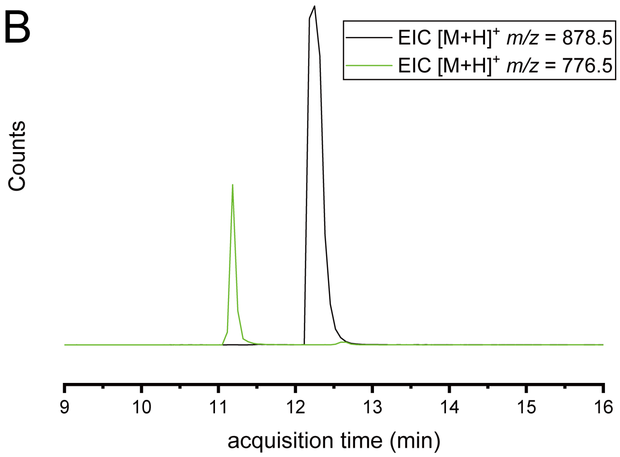

Figure S16: (**A**) Enzymatic reaction of Trp→Ala substrate **6** with BulbE-TE did not yield cyclic product, only hydrolysis product. (**B**) EICs showing the presence of the substrate **6** (black) and the thioester hydrolysis product (green).

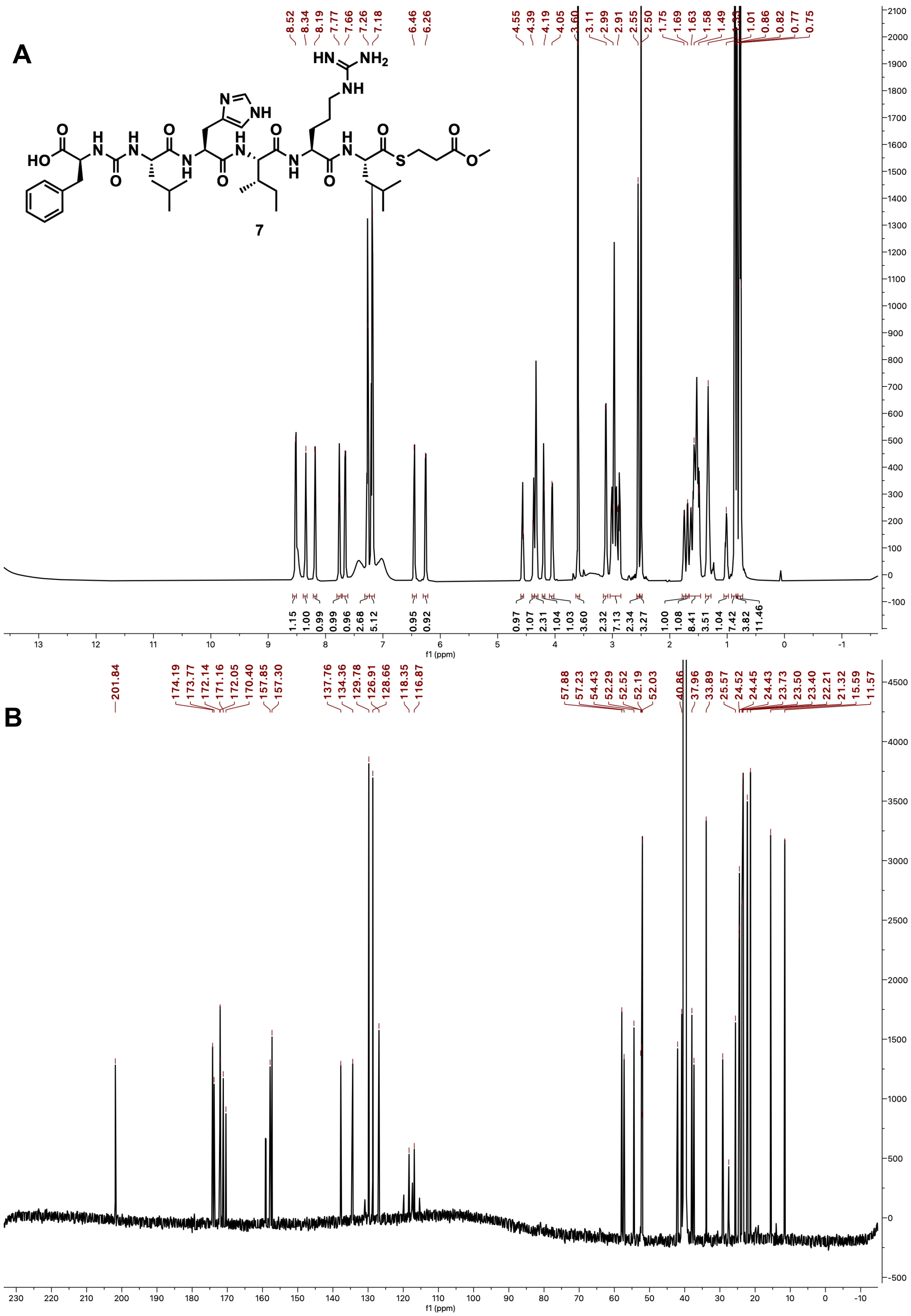

Figure S17: (**A**) ^{1}H (800 MHz) and (**B**) ^{13}C (201 MHz) NMR spectra of **7** in DMSO-d_6.

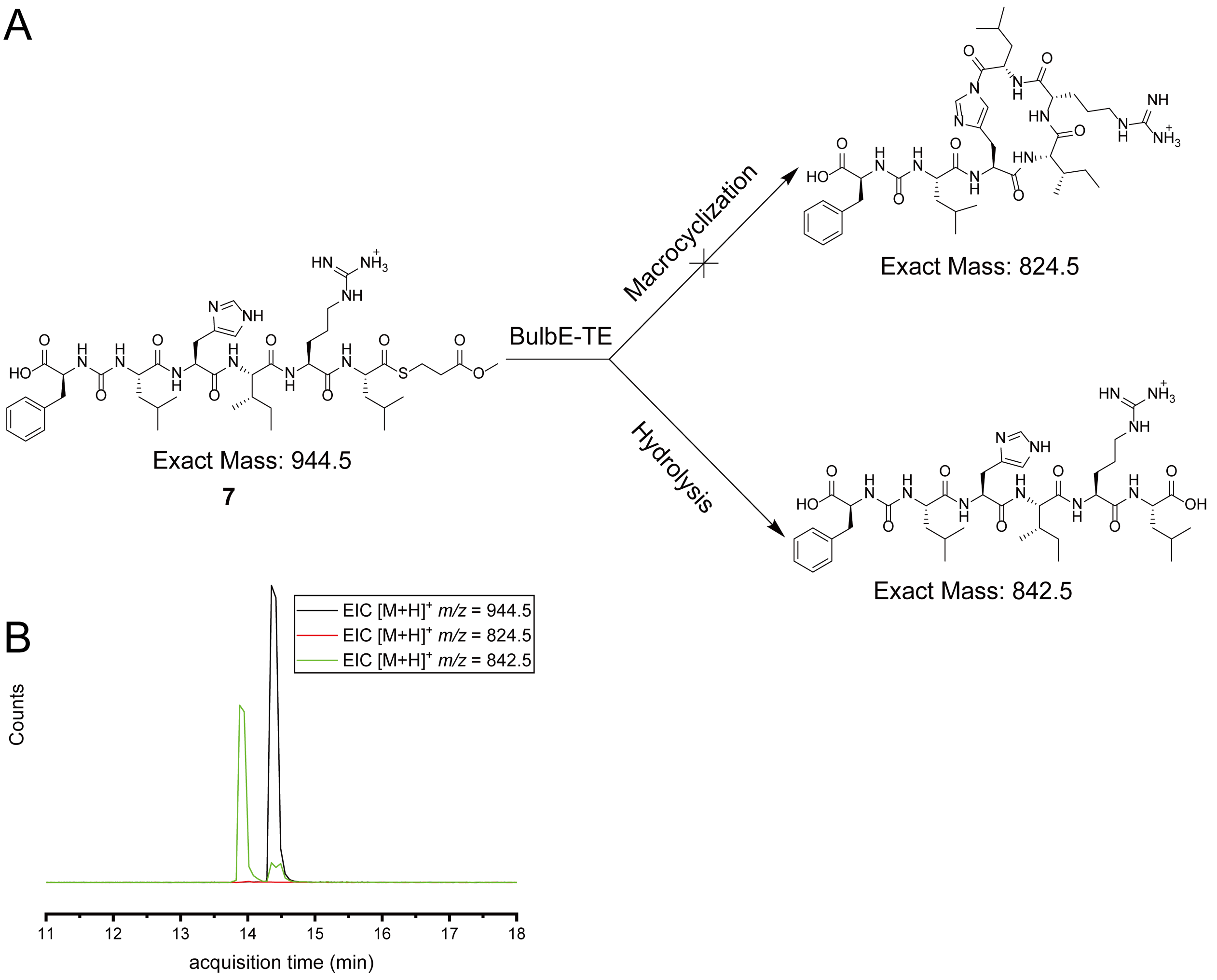

Figure S18: (**A**) Enzymatic reaction of Trp→His substrate **7** with BulbE-TE did not yield cyclic product, only hydrolysis product. (**B**) EICs showing the presence of the substrate **7** (black) and the thioester hydrolysis product (green). No cyclic product (red) was observed.

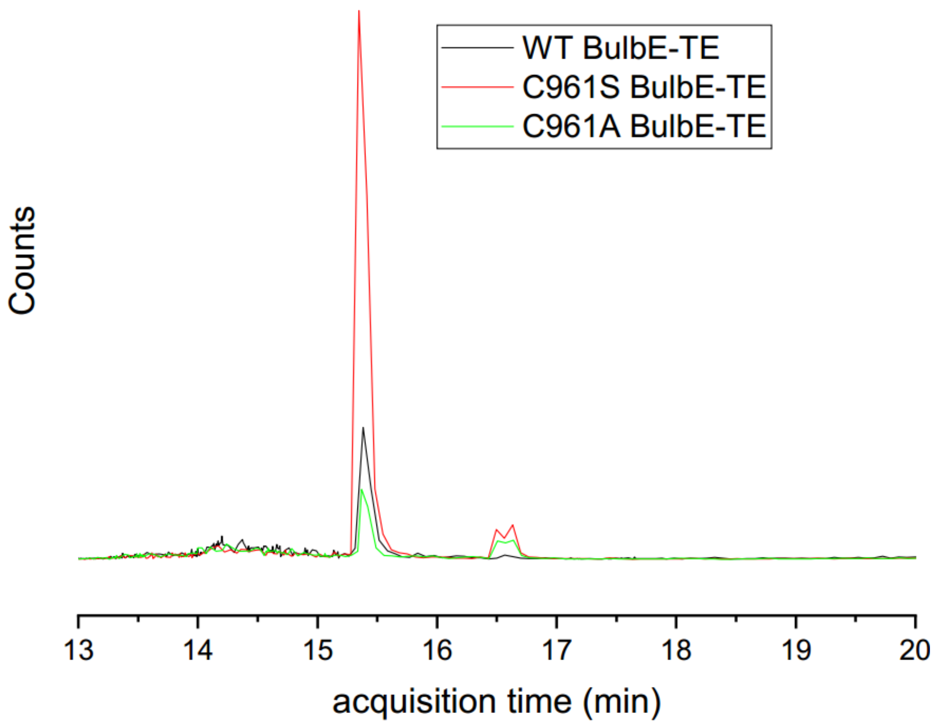

Figure S19: EICs demonstrating abundance of thioester hydrolysis product starting from substrate compound **1** using wild type and mutant forms of BulbE-TE.

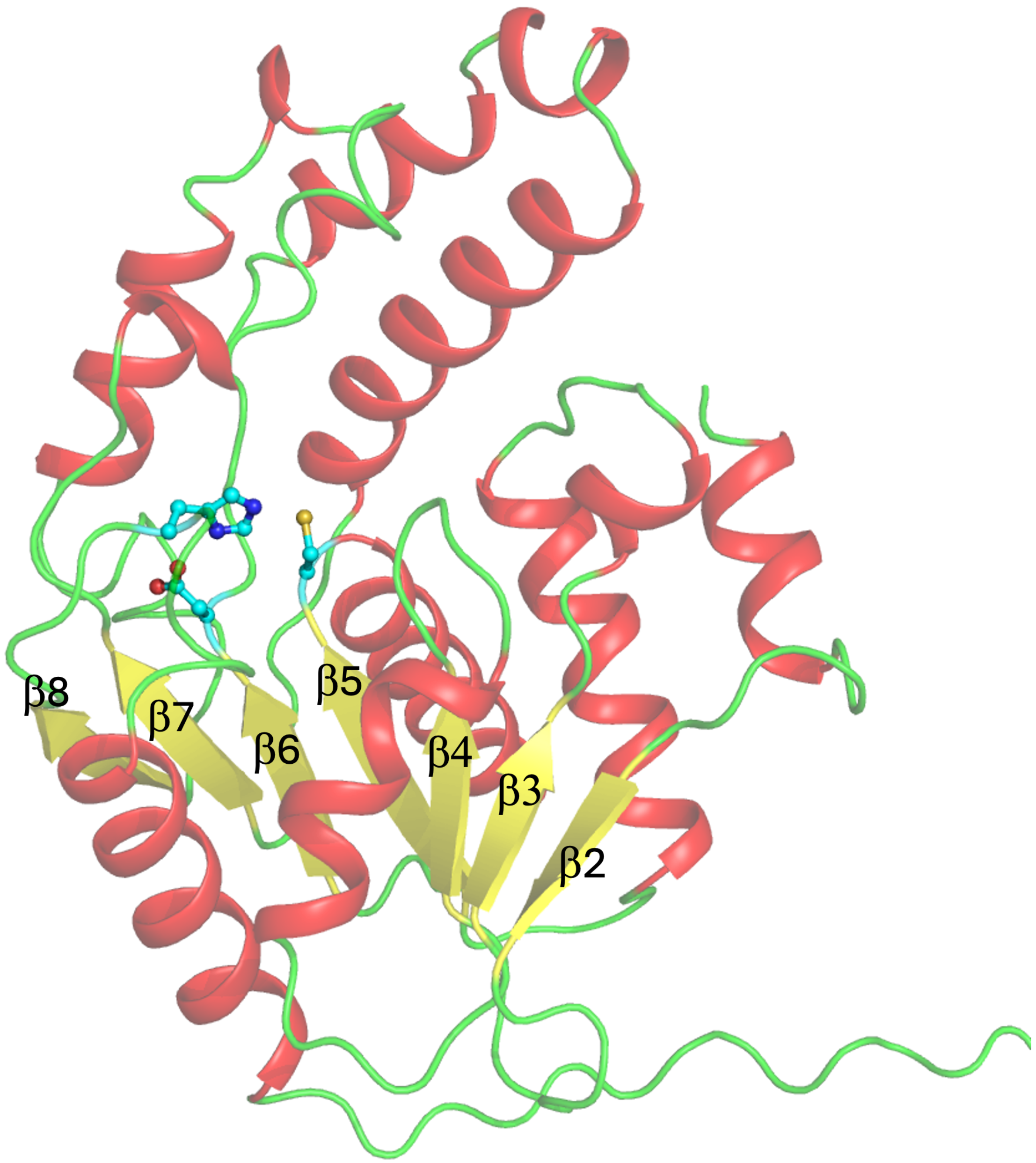

Figure S20: Cartoon representation of the AlphaFold3-generated model of the BulbE-TE with the alpha helices colored red, beta strands colored yellow, and the loop region shown in green. The catalytic triad residues are shown in stick-ball representation with carbon atoms colored cyan. Strands of the central beta-sheet are annotated.

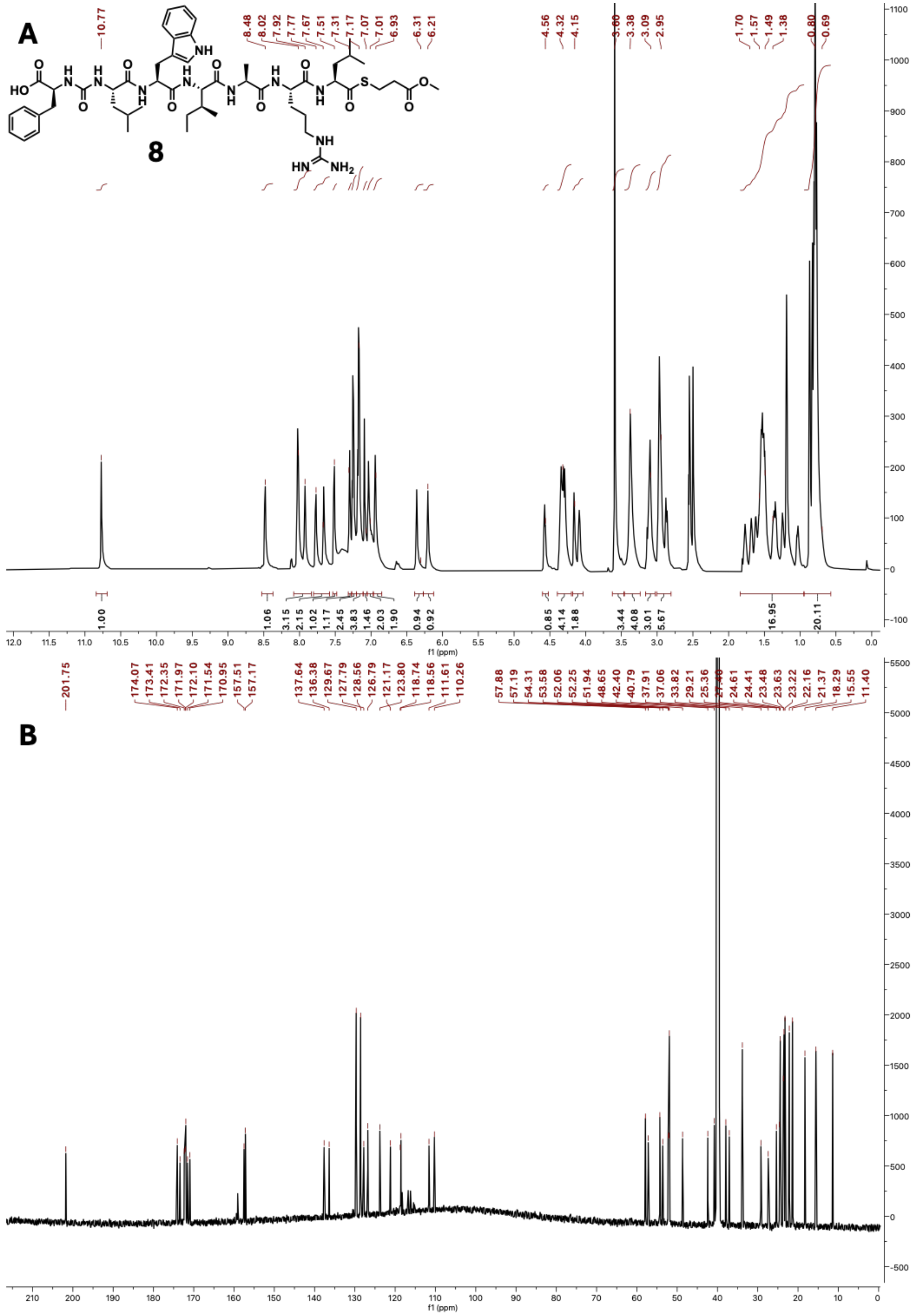

Figure S21: (**A**) ^{1}H (800 MHz) and (**B**) ^{13}C (201 MHz) NMR spectra of **8** in DMSO-d_6.

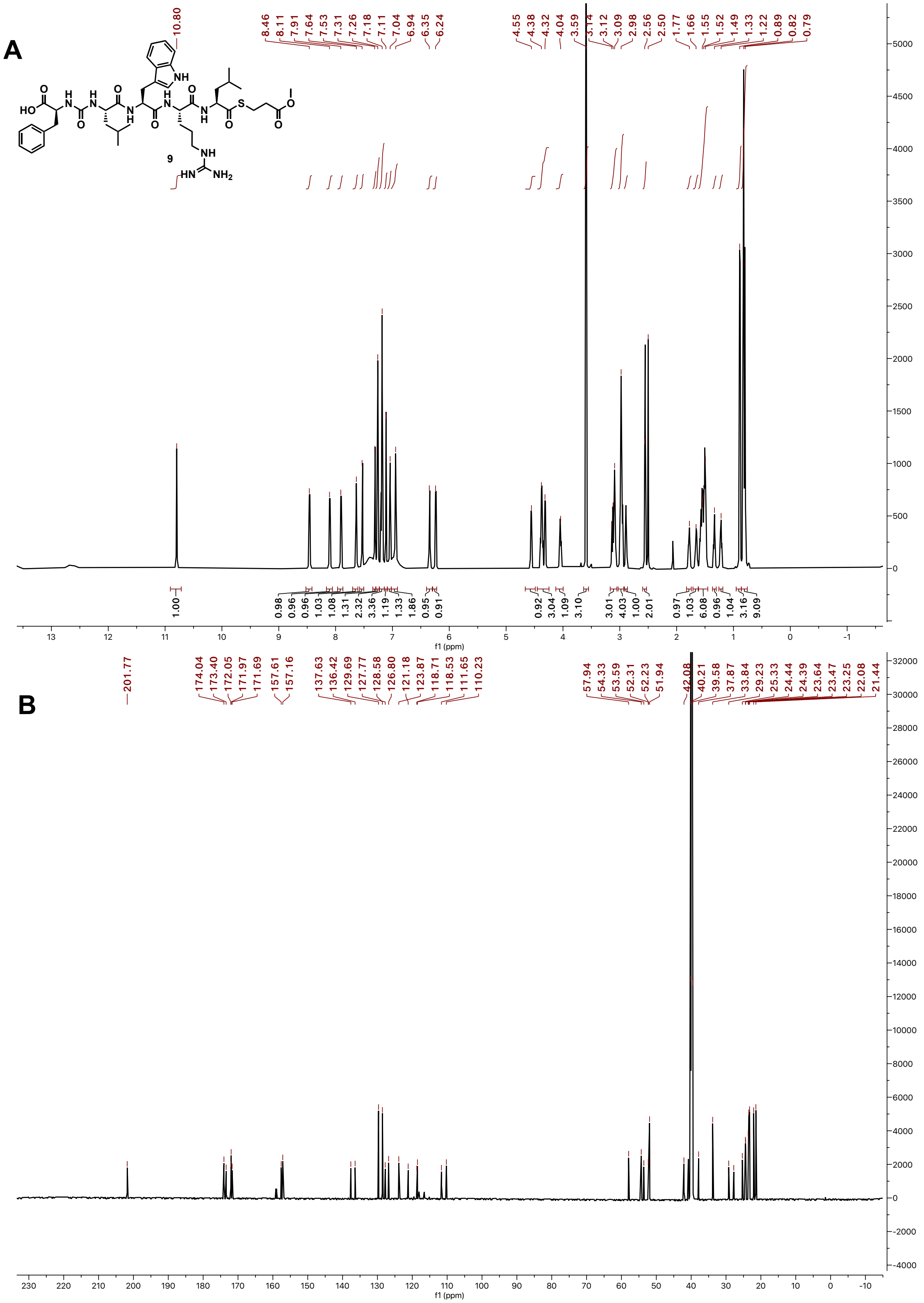

Figure S22: (**A**) ^{1}H (800 MHz) and (**B**) ^{13}C (201 MHz) NMR spectra of **9** in DMSO-d_6.

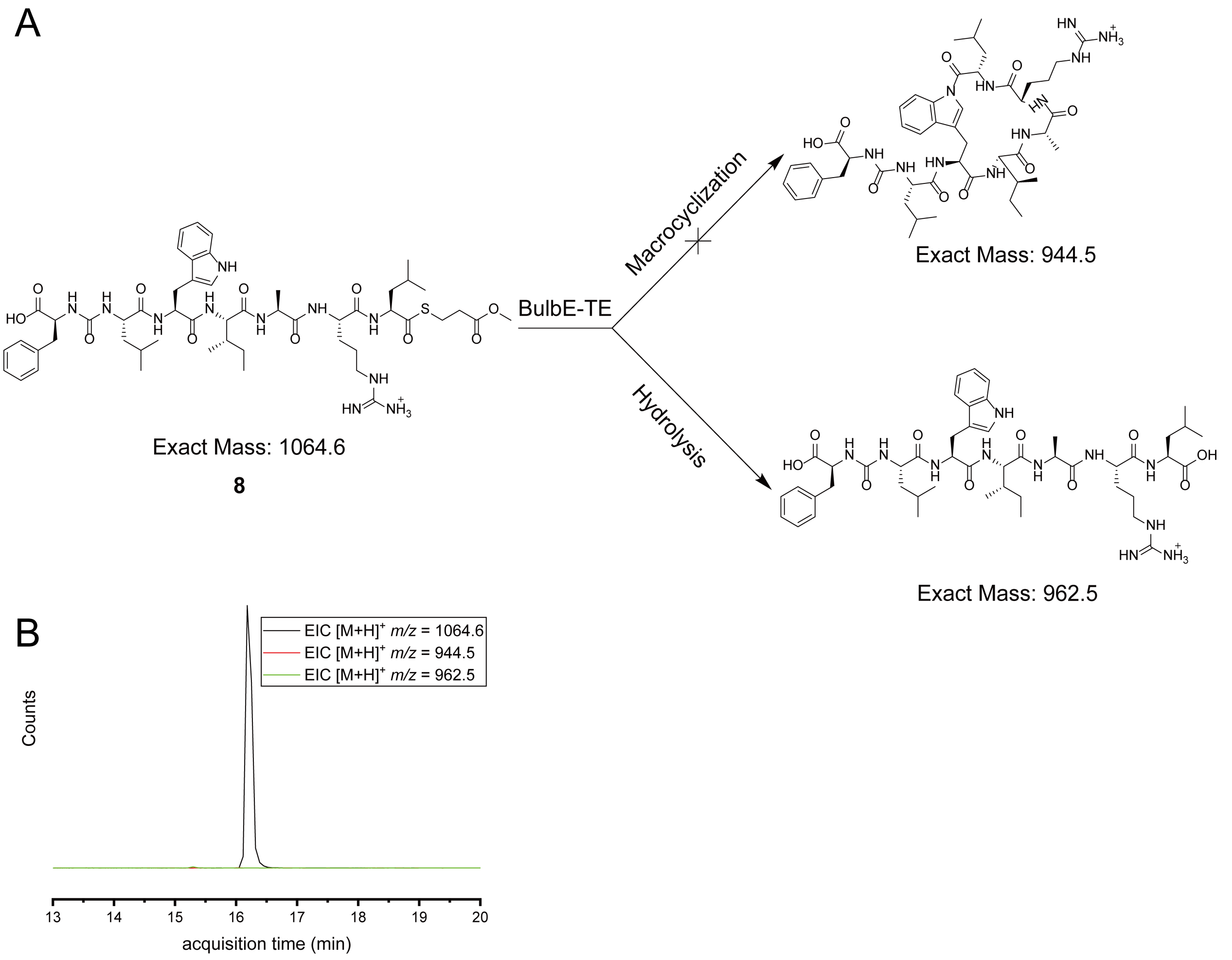

Figure S23: (**A**) Enzymatic reaction of expanded substrate **8** with BulbE-TE did not yield cyclic product, only hydrolysis product. (**B**) EICs showing the presence of the substrate **8** (black) and the thioester hydrolysis product (green). No cyclic product was observed (red). The scale of the EIC y-axis precludes observation of the hydrolysis product.

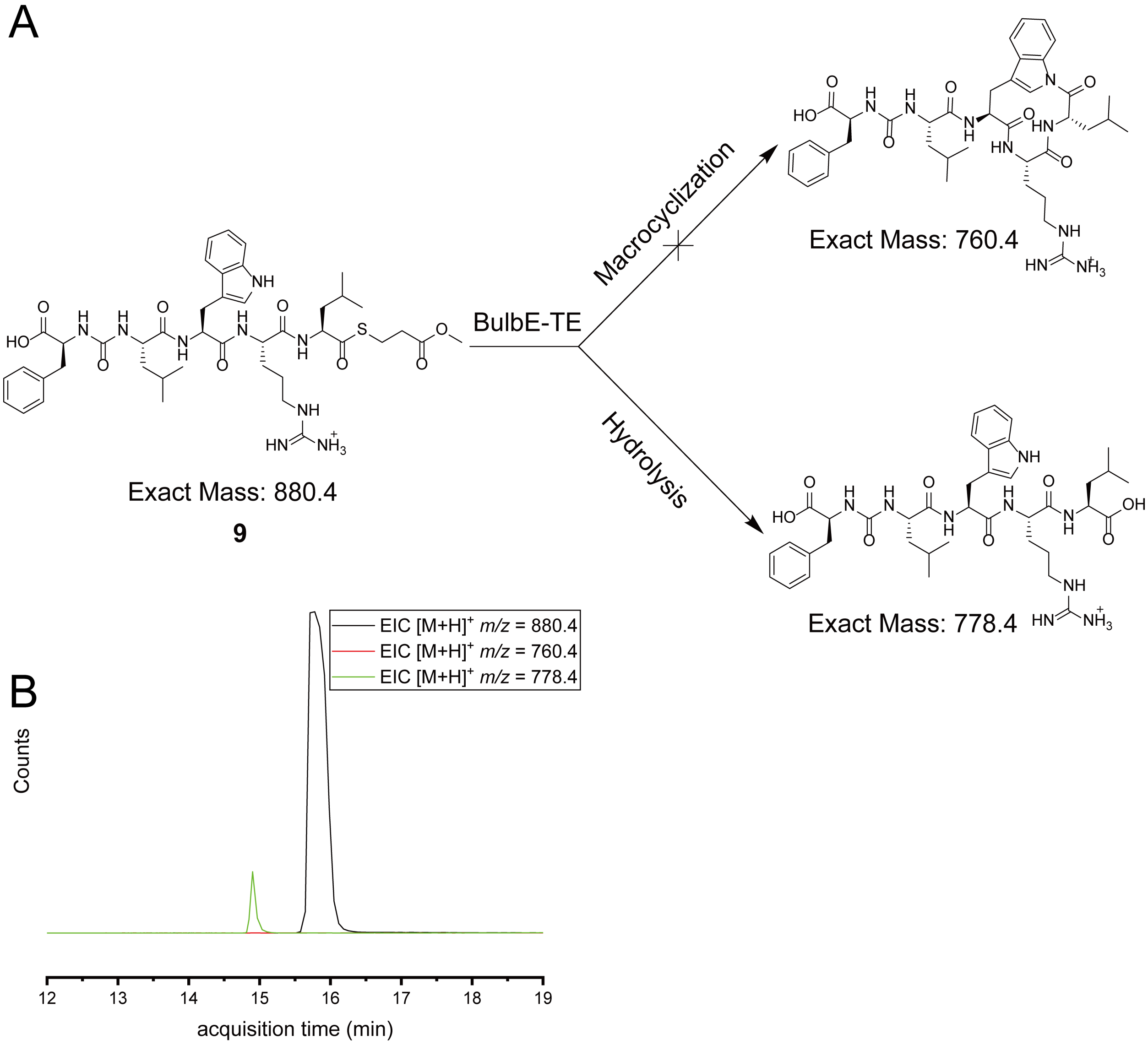

Figure S24: (**A**) Enzymatic reaction of contracted substrate **9** with BulbE-TE did not yield cyclic product, only hydrolysis product. (**B**) EICs showing the presence of the substrate **9** (black) and the thioester hydrolysis product (green). No cyclic product was observed (red).

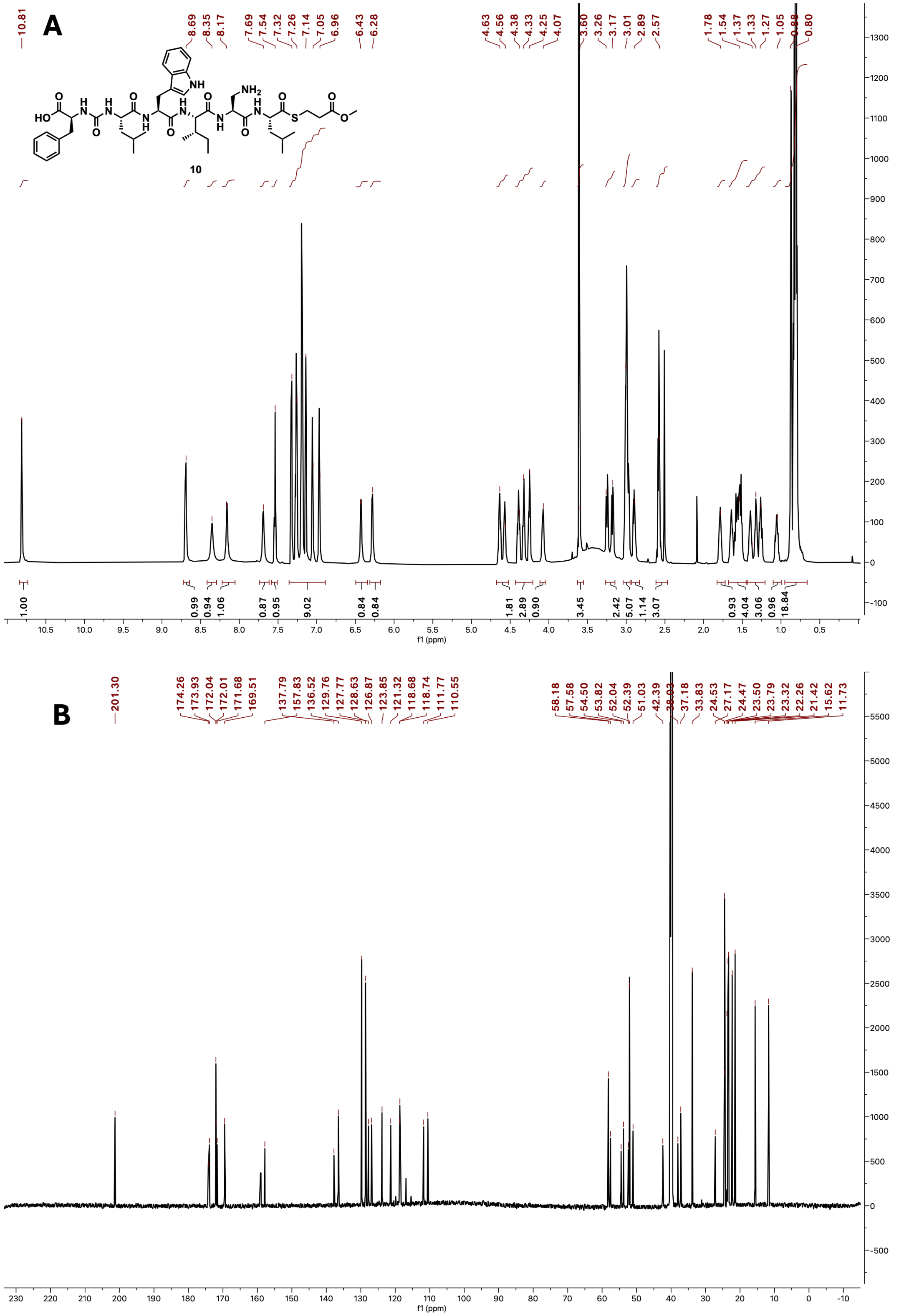

Figure S25: (**A**) ^{1}H (800 MHz) and (**B**) ^{13}C (201 MHz) NMR spectra of **10** in DMSO-d_6.

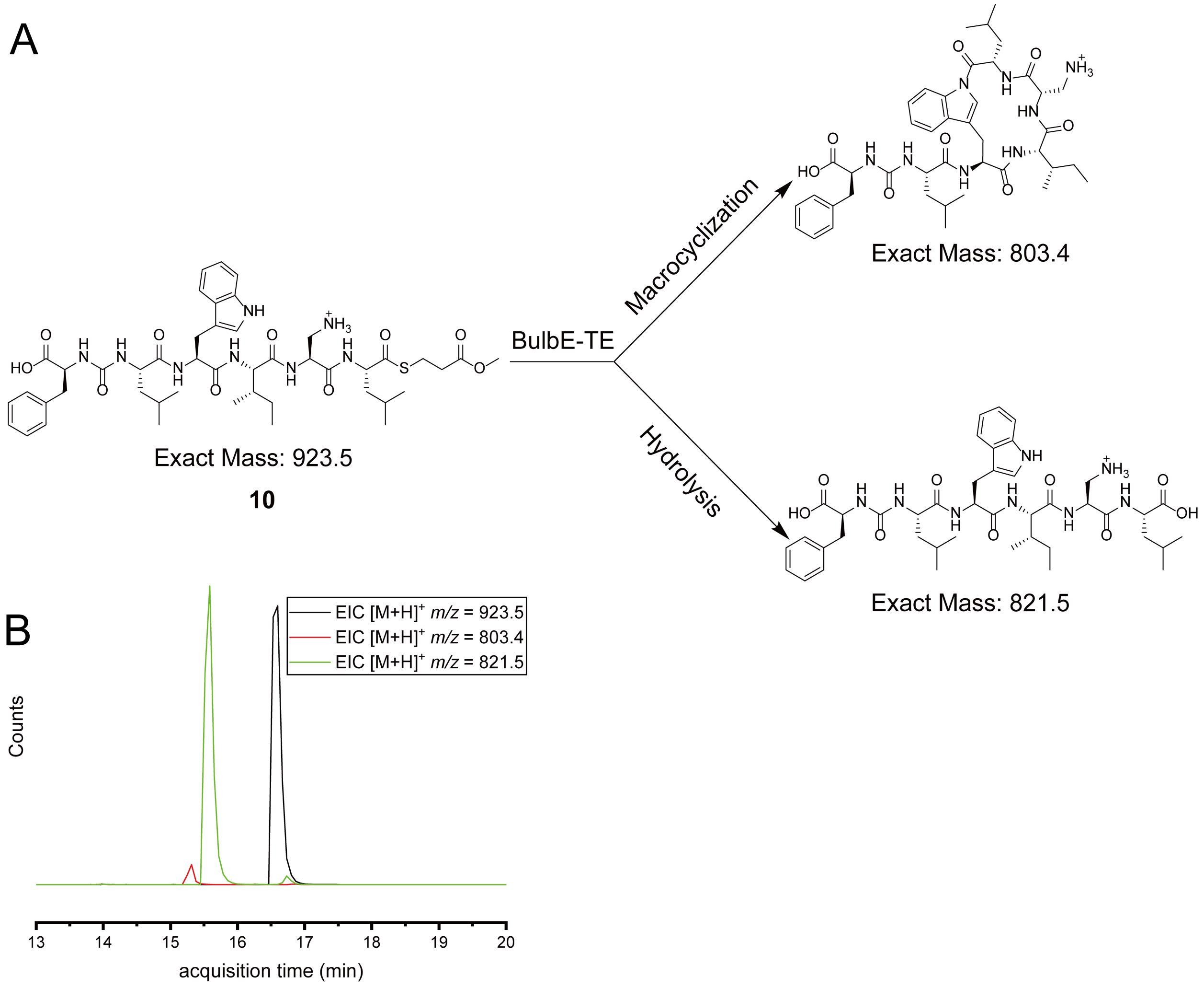

Figure S26: (**A**) Enzymatic reaction of Arg→Dap substrate **10** with BulbE-TE yielded cyclic and hydrolysis products. (**B**) EICs showing the presence of the substrate **10** (black), cyclic product (red), and the thioester hydrolysis product (green).

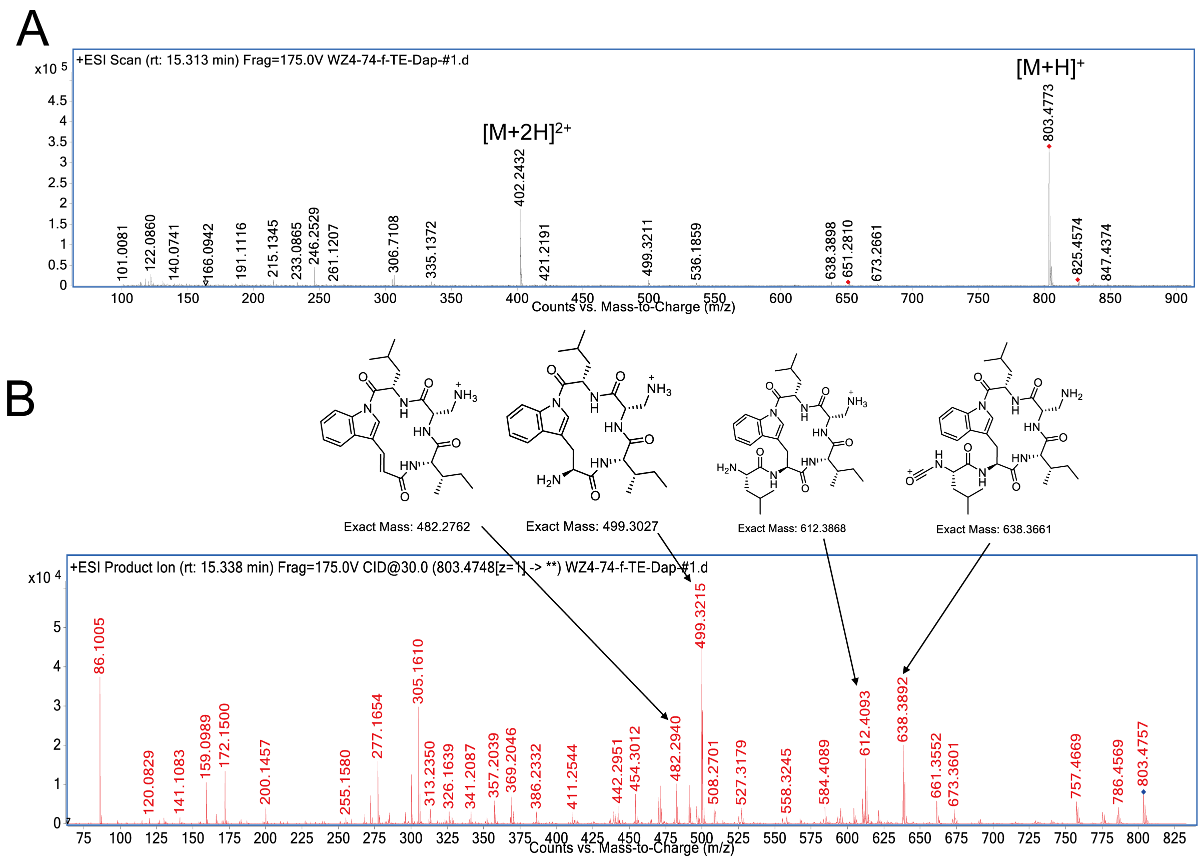

Figure S27: (**A**) MS[1] spectra for the expected macrocyclic product generated by BulbE-TE using **10** as the substrate. Both $[M+H]^{1+}$ and $[M+2H]^{2+}$ ions are observed, as is typical for bulbiferamides. (**B**) MS^2 spectra for the $[M+H]^{1+}$ parent ion with key daughter ions structurally annotated.

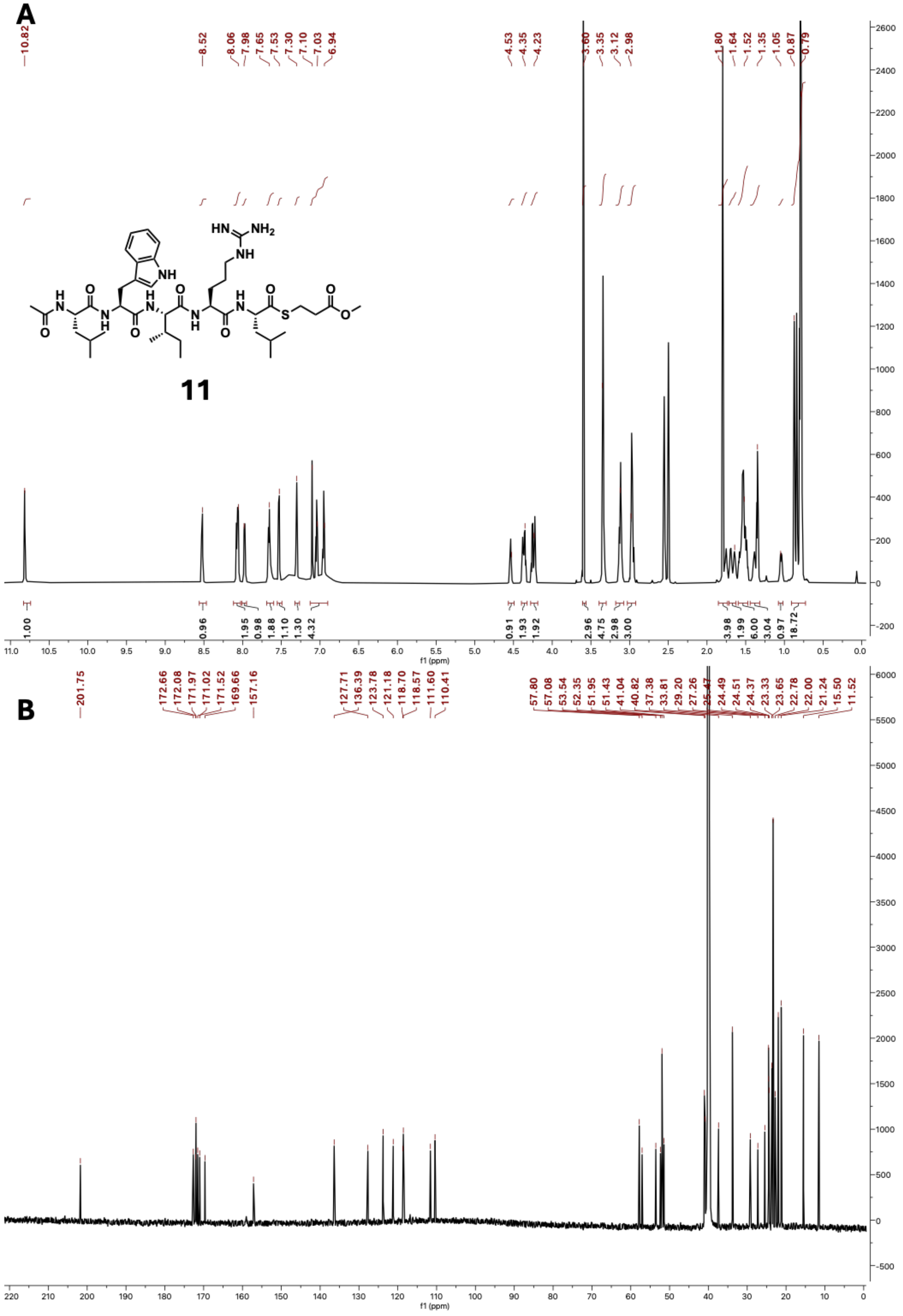

Figure S28: (**A**) ^{1}H (800 MHz) and (**B**) ^{13}C (201 MHz) NMR spectra of **11** in DMSO-d_6.

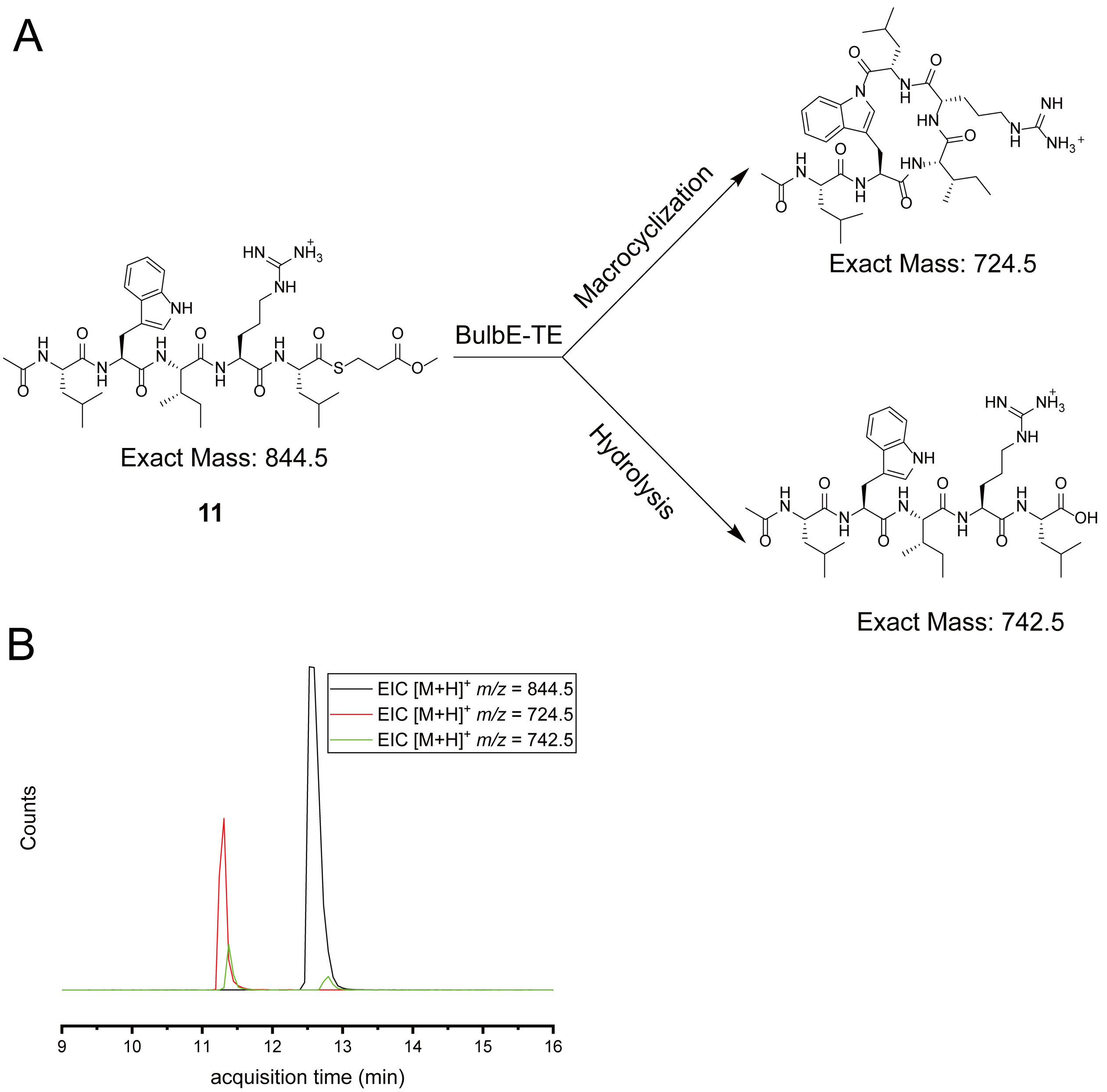

Figure S29: (**A**) Enzymatic reaction of substrate **11** with BulbE-TE yielded cyclic and hydrolysis products. (**B**) EICs showing the presence of the substrate **11** (black), cyclic product (red), and the thioester hydrolysis product (green).

A

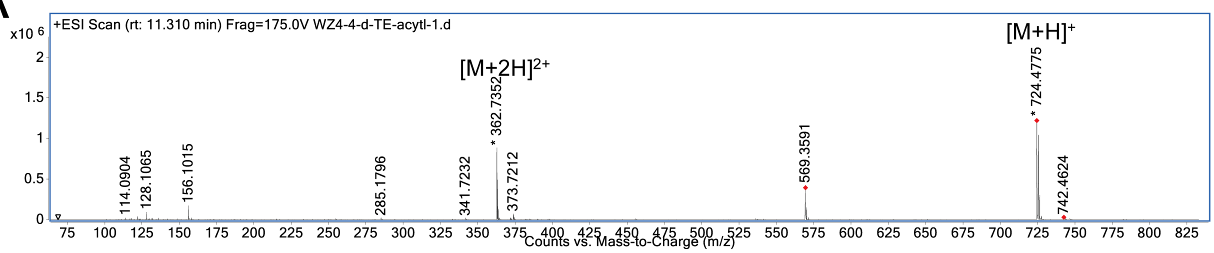

B

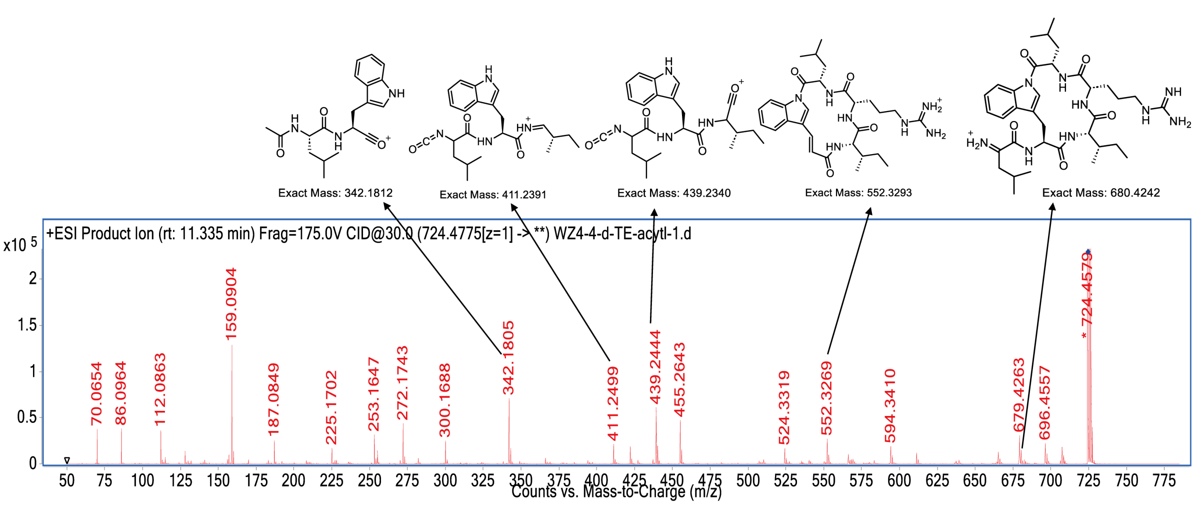

Figure S30: (**A**) MS[1] spectra for the expected macrocyclic product generated by BulbE-TE using **11** as the substrate. Both $[M+H]^{1+}$ and $[M+2H]^{2+}$ ions are observed, as is typical for bulbiferamides. (**B**) MS^2 spectra for the $[M+H]^{1+}$ parent ion with key daughter ions structurally annotated.

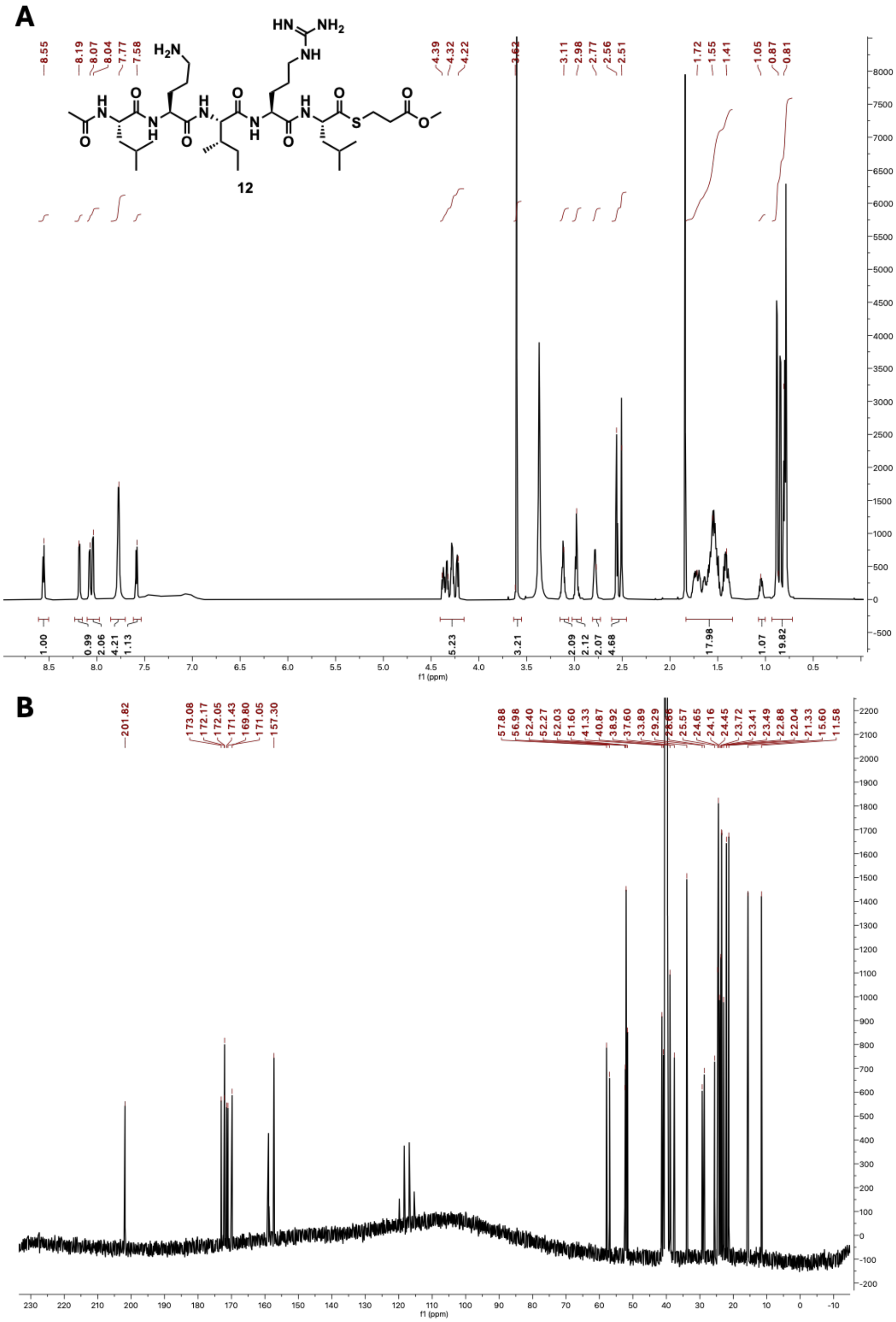

Figure S31: (**A**) ^{1}H (800 MHz) and (**B**) ^{13}C (201 MHz) NMR spectra of **12** in DMSO-d_6.

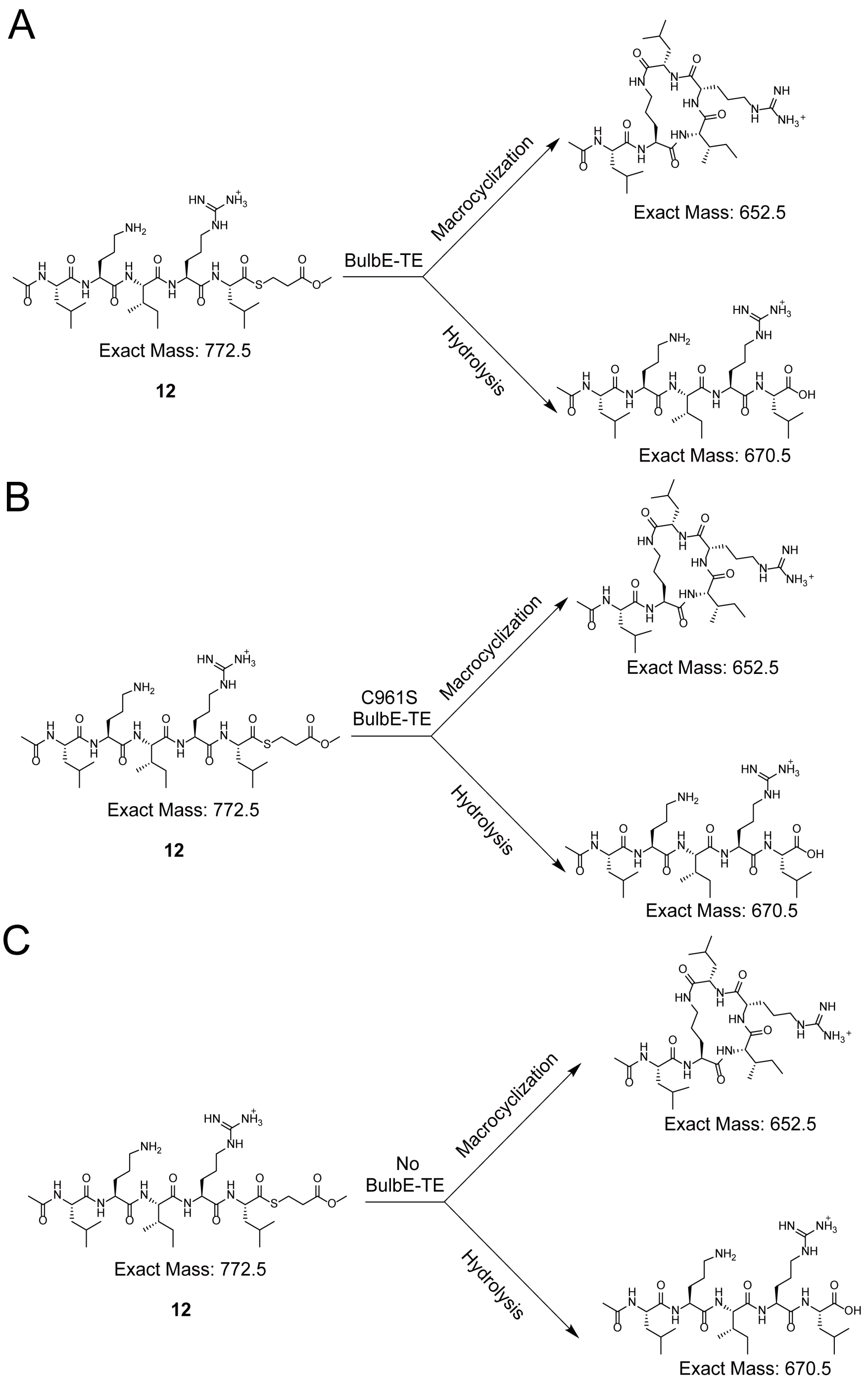

Figure S32: (**A**) Scheme for enzymatic reaction of substrate **12** with BulbE-TE to yield cyclic product and a thioester hydrolysis product. (**B**) Scheme for enzymatic reaction of substrate **12** with C961S BulbE-TE to yield cyclic product and a thioester hydrolysis product. (**C**) Scheme for a negative control reaction in which the enzyme is omitted.

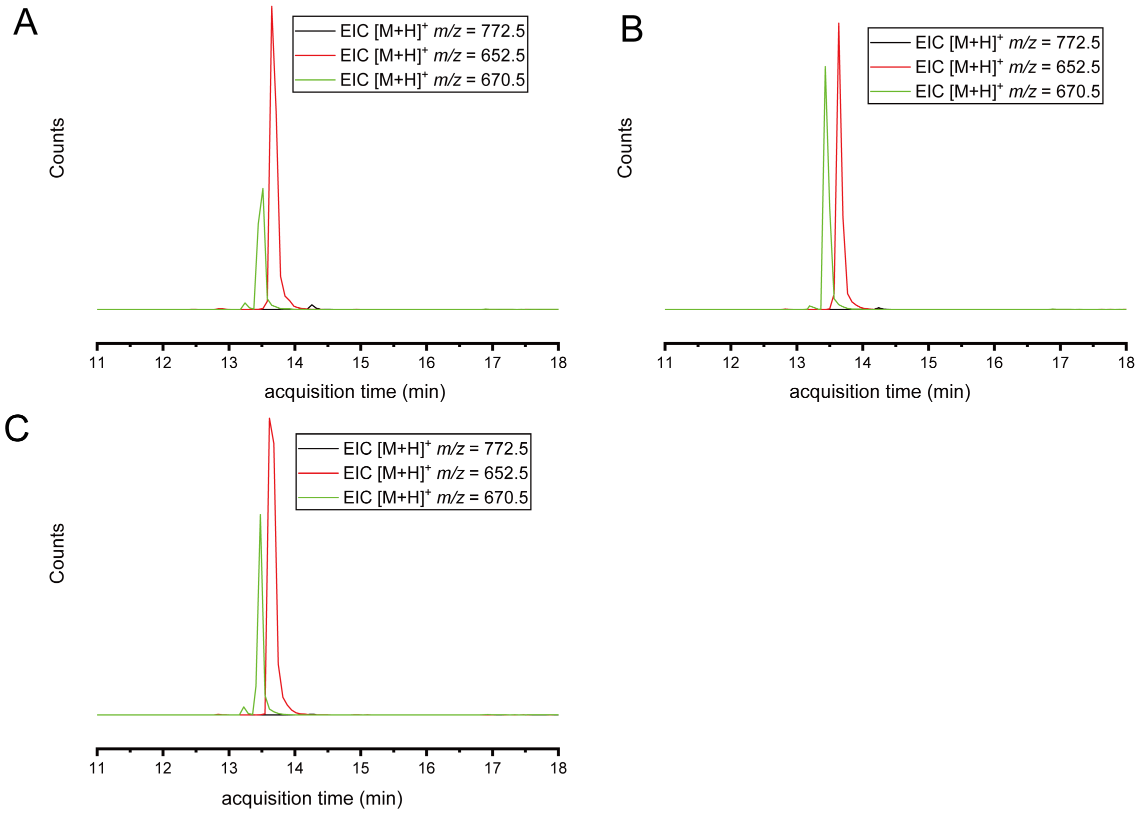

Figure S33: (**A**) EICs showing the presence of the substrate **12** (black), cyclic product (red), and the thioester hydrolysis product (green) in enzymatic reaction substrate **12** with BulbE-TE at pH 7.5. (**B**) EICs showing the presence of the substrate **12** (black), cyclic product (red), and the thioester hydrolysis product (green) in enzymatic reaction substrate **12** with C961S BulbE-TE at pH 7.5. (**C**) EICs showing the presence of the substrate **12** (black), cyclic product (red), and the thioester hydrolysis product (green) in enzymatic reaction substrate **12** without enzyme at pH 7.5.

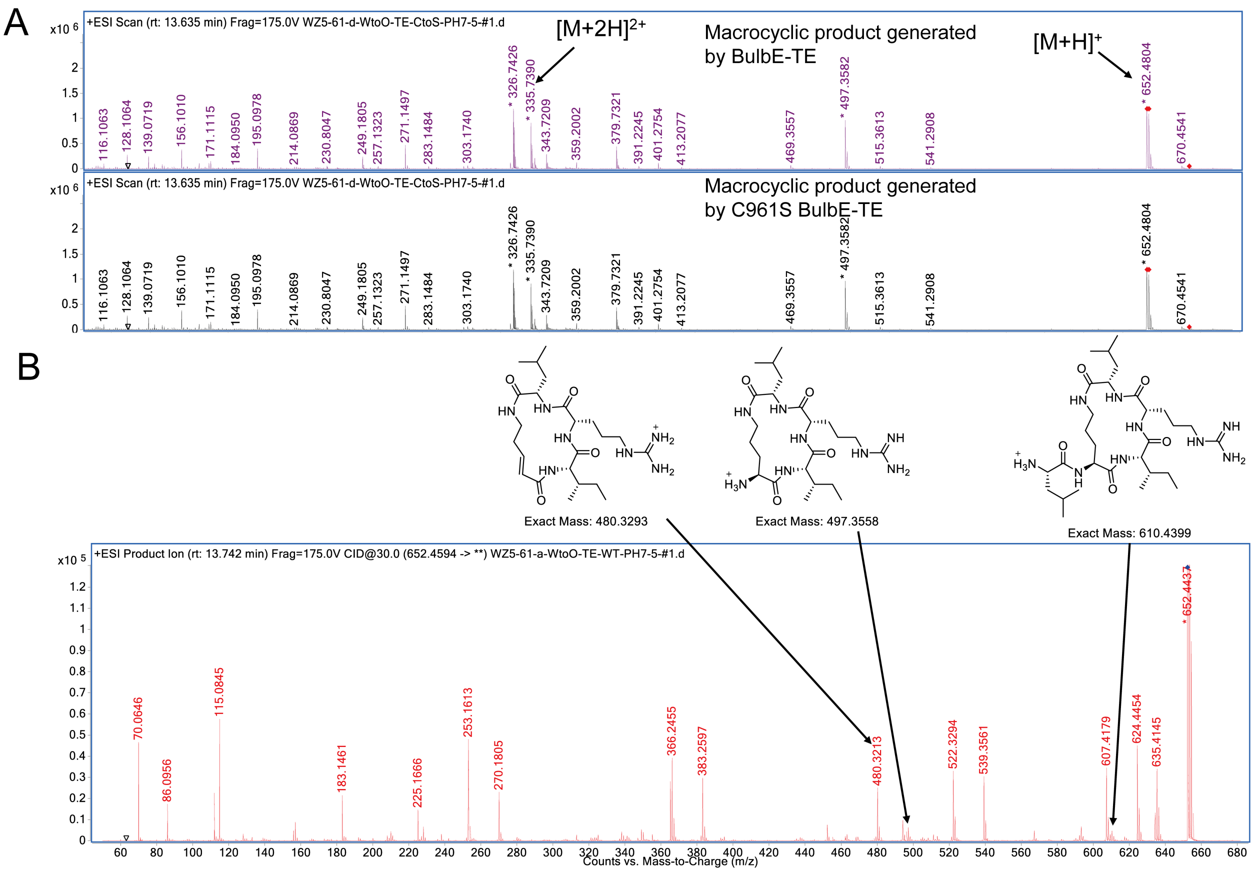

Figure S34: (**A**) MS[1] spectra for the expected macrocyclic product generated by BulbE-TE (top) and C961S BulbE-TE (bottom) using **12** as the substrate. Both $[M+H]^{1+}$ and $[M+2H]^{2+}$ ions are observed, as is typical for bulbiferamides. (**B**) MS[2] spectra for the $[M+H]^{1+}$ parent ion with key daughter ions structurally annotated for the macrocyclic product.

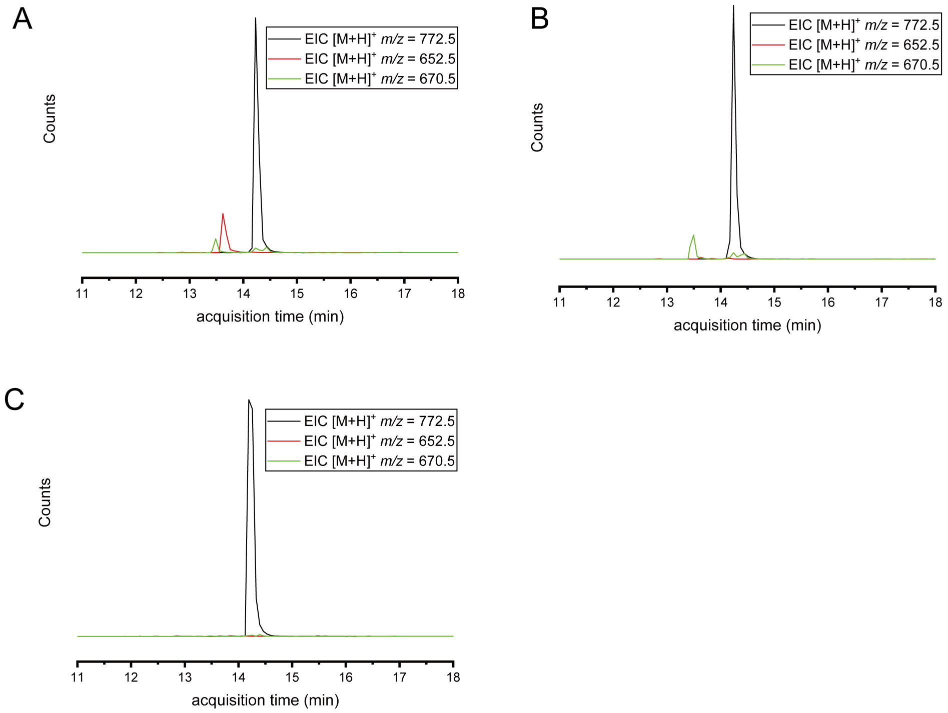

Figure S35: (**A**) EICs showing the presence of the substrate **12** (black), cyclic product (red), and the thioester hydrolysis product (green) in enzymatic reaction substrate **12** with BulbE-TE at pH 6.0. (**B**) EICs showing the presence of the substrate **12** (black), cyclic product (red), and the thioester hydrolysis product (green) in enzymatic reaction substrate **12** with C961S BulbE-TE at pH 6.0. (**C**) EICs showing the presence of the substrate **12** (black), cyclic product (red), and the thioester hydrolysis product (green) in enzymatic reaction substrate **12** with C961S without enzyme at pH 6.0.

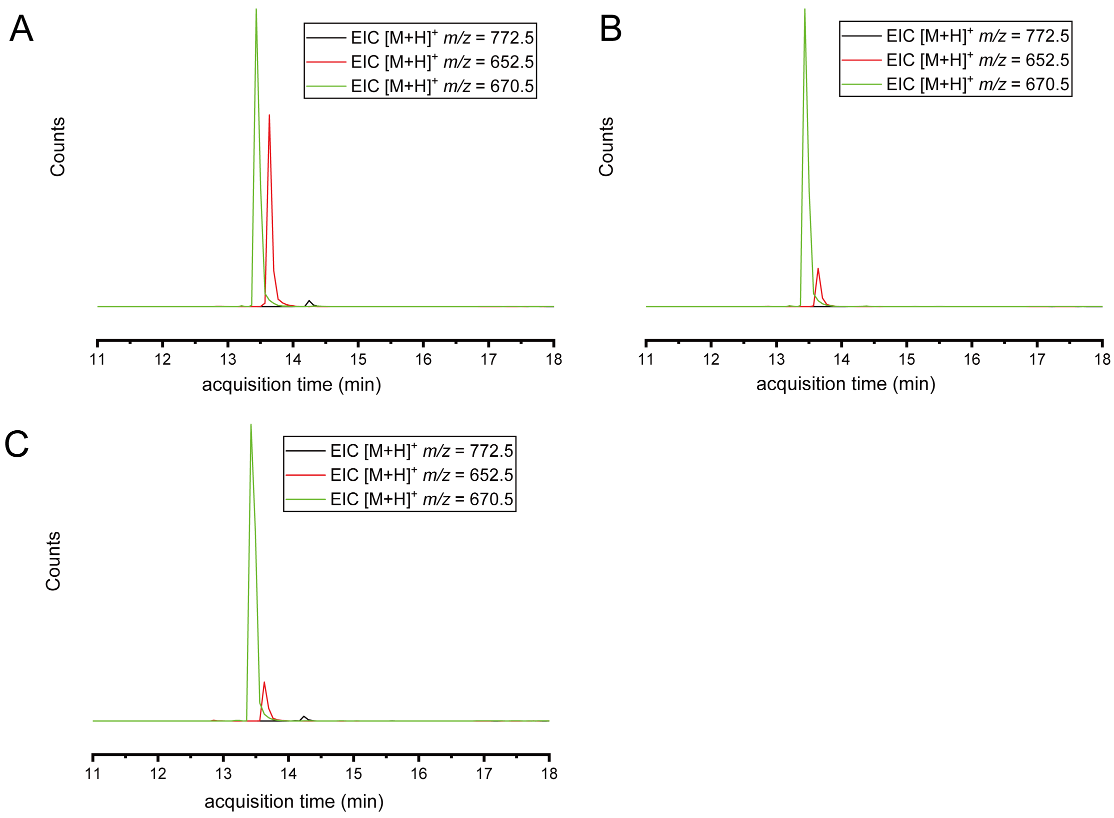

Figure S36: (**A**) EICs showing the presence of the substrate **12** (black), cyclic product (red), and the thioester hydrolysis product (green) in enzymatic reaction substrate **12** with BulbE-TE at pH 9.0. (**B**) EICs showing the presence of the substrate **12** (black), cyclic product (red), and the thioester hydrolysis product (green) in enzymatic reaction substrate **12** with C961S BulbE-TE at pH 9.0. (**C**) EICs showing the presence of the substrate **12** (black), cyclic product (red), and the thioester hydrolysis product (green) in enzymatic reaction substrate **12** without enzyme at pH 9.0.

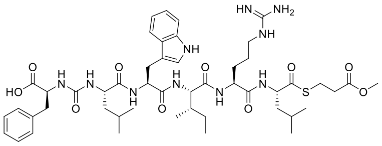

1

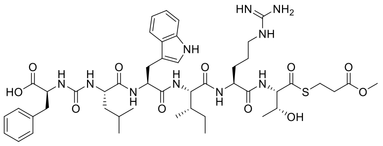

2

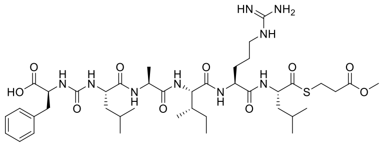

6

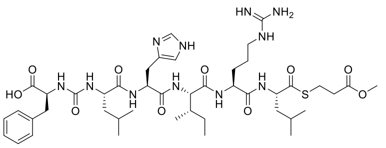

7

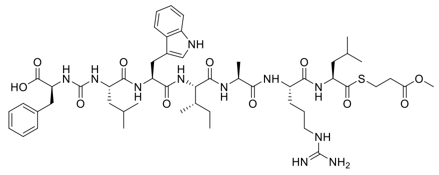

8

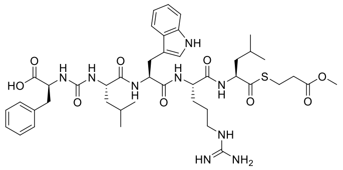

9

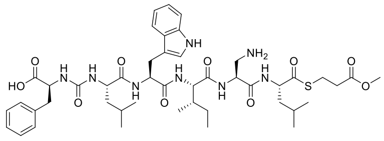

10

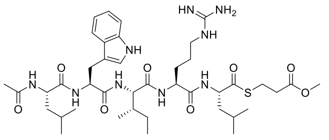

11

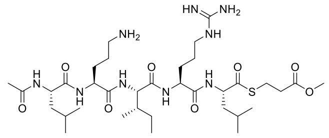

12

Figure S37: Chemical structures of substrates tested in this study.

Supplementary References

(1) Blanco-Canosa, J. B.; Nardone, B.; Albericio, F.; Dawson, P. E. Chemical Protein Synthesis Using a Second-generation N-acylurea Linker for the Preparation of Peptide-thioester Precursors. *J. Am. Chem. Soc.* **2015**, *137*, 7197–7209.
